# Supplementary material for: Geographical and sociodemographic differences in statin dispensation after acute myocardial infarction in Sweden: a register-based prospective cohort study applying analysis of individual heterogeneity and discriminatory accuracy (AIHDA) for basic comparisons of healthcare quality
Source: BMJ Open. 2023 Sep 28;13(9):e063117. doi: 10.1136/bmjopen-2022-063117 (PMC10546129; doi:10.1136/bmjopen-2022-063117)
Supplement: Supplementary data [file bmjopen-2022-063117supp001.pdf]

SUPPLEMENTARY MATERIAL (S1)

S1: Stata codes

Main analysis

```
. use cohort_selected.dta, clear

. generate time = 1
. stset time, failure(statins) id(lpnr)

* Model 1a
. stcox ib11.region, vce(robust)
. predict p1a

* Model 1b
. stcox ib12.inter, vce(robust)
. predict p1b

* Model 2
. stcox ib11.region ib12.inter, vce(robust)
. predict p2

. roccomp statins p1a p1b p2
```

Prevalences

```
. use cohort_selected.dta, clear
. by region, sort: egen cases = sum(statins)
. by region: gen total = _N
. keep cases total region
. duplicates drop

. use cohort_selected.dta, clear
. by inter, sort: egen cases = sum(statins)
. by inter: generate total = _N
. keep cases total inter
. duplicates drop

. use cohort_selected.dta, clear
. egen cases = sum(statins)
. display _N
. tabulate cases
```


Merlo J, *et al. BMJ Open* 2023; 13:e063117. doi: 10.1136/bmjopen-2022-063117

Supplementary analysis

```
. use cohort_selected.dta, clear

. generate time = 1
. stset time, failure(statins) id(lpnr)

* Model 1b_age
. stcox ib1.age_cat, vce(robust)
. predict p2a

* Model 1b_sex
. stcox i.male, vce(robust)
. predict p2b

* Model 1b_income
. stcox i.cum, vce(robust)
. predict p2c

* Model 1b_country
. stcox i.Sw, vce(robust)
. predict p2d

* Model 1b_all
. stcox ib1.age_cat i.male i.cum i.Sw, vce(robust)
. predict p2e

. roccomp statins p2*
```


Merlo J, *et al. BMJ Open* 2023; 13:e063117. doi: 10.1136/bmjopen-2022-063117
