# Supplementary material for: Geographical and sociodemographic differences in statin dispensation after acute myocardial infarction in Sweden: a register-based prospective cohort study applying analysis of individual heterogeneity and discriminatory accuracy (AIHDA) for basic comparisons of healthcare quality
Source: BMJ Open. 2023 Sep 28;13(9):e063117. doi: 10.1136/bmjopen-2022-063117 (PMC10546129; doi:10.1136/bmjopen-2022-063117)
Supplement: Supplementary data [file bmjopen-2022-063117supp002.pdf]

SUPPLEMENTARY MATERIAL (S2)

S2: Supplementary analyses

Prevalence Ratios (PR) and (95% confidence intervals) for use of statins 12-18 months after hospital discharge with an acute myocardial infarction (AMI) diagnosis in patients aged, 40-80 years old from 24 sociodemographic strata in the period 2011-2013

	Age	Sex	Income	Country of birth	All	Multicategorical *
Age						
40-64	1.03 (1.02-1.04)				1.02 (1.01-1.04)	
65-80	Reference					
Sex						
Women		Reference				
Men		1.14 (1.12-1.15)			1.13 (1.12-1.14)	
Income						
Low			Reference			
Middle			1.03 (1.01-1.04)		1.03 (1.01-1.04)	
High			1.07 (1.06-1.09)		1.06 (1.05-1.08)	
Country of birth						
Immigrant				Reference		
Native				1.03 (1.01-1.04)	1.01 (1.00-1.03)	
AUC	0.512	0.579	0.546	0.511	0.608	0.609

*Model 1B in table 2. AUC: area under the ROC curve
