# Supplementary material for: Geographical and sociodemographic differences in statin dispensation after acute myocardial infarction in Sweden: a register-based prospective cohort study applying analysis of individual heterogeneity and discriminatory accuracy (AIHDA) for basic comparisons of healthcare quality
Source: BMJ Open. 2023 Sep 28;13(9):e063117. doi: 10.1136/bmjopen-2022-063117 (PMC10546129; doi:10.1136/bmjopen-2022-063117)
Supplement: Author's manuscript [file bmjopen-2022-063117.draft_revisions.pdf]

BMJ Open is committed to open peer review. As part of this commitment we make the peer review history of every article we publish publicly available.

When an article is published we post the peer reviewers' comments and the authors' responses online. We also post the versions of the paper that were used during peer review. These are the versions that the peer review comments apply to.

The versions of the paper that follow are the versions that were submitted during the peer review process. They are not the versions of record or the final published versions. They should not be cited or distributed as the published version of this manuscript.

BMJ Open is an open access journal and the full, final, typeset and author-corrected version of record of the manuscript is available on our site with no access controls, subscription charges or pay-per-view fees (http://bmjopen.bmj.com).

If you have any questions on BMJ Open's open peer review process please email info.bmjopen@bmj.com

BMJ Open

A simple analysis of individual heterogeneity and discriminatory accuracy (AIHDA) for basic comparisons of healthcare performance: The case of geographic and sociodemographic differences in statin dispensation after acute myocardial infarction

Journal:	*BMJ Open*
Manuscript ID	bmjopen-2022-063117
Article Type:	Original research
Date Submitted by the Author:	23-Mar-2022

Complete List of Authors:	Merlo, Juan; Lunds University Faculty of Medicine, Social Epidemiology<br>Öberg, Johan; Lund University Clinical Sciences, Unit for Social Epidemiology<br>Khalaf, Kani; Lund University, Department of Clinical Sciences<br>Perez-Vicente, Raquel; Lund University, Faculty of Medicine, Social Epidemiology<br>Leckie, George; University of Bristol, Multilevel Modelling

Keywords:	EPIDEMIOLOGY, Quality in health care < HEALTH SERVICES ADMINISTRATION & MANAGEMENT, Myocardial infarction < CARDIOLOGY

SCHOLARONE™ Manuscripts

I, the Submitting Author has the right to grant and does grant on behalf of all authors of the Work (as defined in the below author licence), an exclusive licence and/or a non-exclusive licence for contributions from authors who are: i) UK Crown employees; ii) where BMJ has agreed a CC-BY licence shall apply, and/or iii) in accordance with the terms applicable for US Federal Government officers or employees acting as part of their official duties; on a worldwide, perpetual, irrevocable, royalty-free basis to BMJ Publishing Group Ltd ("BMJ") its licensees and where the relevant Journal is co-owned by BMJ to the co-owners of the Journal, to publish the Work in this journal and any other BMJ products and to exploit all rights, as set out in our licence.

The Submitting Author accepts and understands that any supply made under these terms is made by BMJ to the Submitting Author unless you are acting as an employee on behalf of your employer or a postgraduate student of an affiliated institution which is paying any applicable article publishing charge ("APC") for Open Access articles. Where the Submitting Author wishes to make the Work available on an Open Access basis (and intends to pay the relevant APC), the terms of reuse of such Open Access shall be governed by a Creative Commons licence – details of these licences and which Creative Commons licence will apply to this Work are set out in our licence referred to above.

Other than as permitted in any relevant BMJ Author's Self Archiving Policies, I confirm this Work has not been accepted for publication elsewhere, is not being considered for publication elsewhere and does not duplicate material already published. I confirm all authors consent to publication of this Work and authorise the granting of this licence.

For peer review only

A simple analysis of individual heterogeneity and discriminatory accuracy (AIHDA) for basic comparisons of healthcare performance: The case of geographic and sociodemographic differences in statin dispensation after acute myocardial infarction

Juan Merlo[1, 2] ORCID: 0000-0001-8379-9708

Johan Öberg[1, 3] ORCID: 0000-0002-0701-5155

Kani Khalaf[1, 3] ORCID: 0000-0002-6749-7037

Raquel Perez Vicente[1] ORCID: 0000-0002-6273-1656

George Leckie[4] ORCID: 0000-0003-1486-745X

[1] Unit for Social Epidemiology, Faculty of Medicine, Lund University, Malmö, Sweden

[2] Centre for Primary Health Care Research, Region Skåne, Malmö, Sweden

[3] Department of Health and Medical Care Management, Region Skåne, Malmö, Sweden

[4] Centre for Multilevel Modelling, University of Bristol, UK

Corresponding author: Johan Öberg[1, 3] ORCID: 0000-0002-0701-5155

johan.oberg@skane.se; johan.oberg@med.lu.se

For peer review only - http://bmjopen.bmj.com/site/about/guidelines.xhtml

For peer review only

For peer review only - http://bmjopen.bmj.com/site/about/guidelines.xhtml

Abstract

Objectives: In Sweden as in many other countries, official monitoring of healthcare performance is mostly focused on geographical disparities in relation to a desirable benchmark. However, current evaluations could be improved by considering (i) the intersection of other relevant axes of inequity like age, sex, income, and migration status and also (ii) the existence of individual heterogeneity around geographical averages. Therefore, we aimed to evaluate both geographical and sociodemographic inequalities and illustrate how the Analysis of Individual Heterogeneity and Discriminatory Accuracy (AIHDA) enhances such evaluations.

Population and methods: We applied AIHDA and calculated the area under the ROC curve (AUC) of regional and sociodemographic differences in the statin dispensations of 35,044 patients from 21 Swedish regions who were discharged from the hospital with an AMI diagnosis between January 2011 and December 2013. Following the Swedish National Board of Health and Welfare, we used a benchmark value of 90%.

Results: Dispensation of stains after AMI in Sweden did not reach the desired target of 90%. Regional differences were absent/very small (AUC = 0.537) while sociodemographic differences were small (AUC = 0.618). Women, especially those with immigrant background and older than 65 years have the lowest proportions of statin dispensations after AMI.

For peer review only - http://bmjopen.bmj.com/site/about/guidelines.xhtml

Conclusions: As the AUC statistics are small, interventions trying to achieve the benchmark value should be universal. However, a special emphasis should nevertheless be directed towards older women with immigrant backgrounds. The AIHDA framework is easy to implement and improves the evaluation of geographic and sociodemographic inequalities in healthcare performance.

Strengths and limitations of this study:

- The applied Analysis of Individual Heterogeneity and Discriminatory Accuracy (AIHDA) provides an improved mapping and quantification of geographical and sociodemographic inequalities in healthcare quality
- The quality of the data in our study is high as it is based on national registers with valid and standardized information on diagnoses, medication use and sociodemographic variables
- We can directly compare our results to those presented by the Swedish National Boars of Health and Welfare since the definition of the quality indicator is the same.
- The definition and categorization of the sociodemographic variables is coarse. However, more fine categorizations would excessively increase the number of strata

For peer review only - http://bmjopen.bmj.com/site/about/guidelines.xhtml

- The AIHDA approach can be implemented using both traditional regression or more advanced multilevel models which provide several advantages. However, traditional regression is a valid and accessible alternative

For peer review only

For peer review only - http://bmjopen.bmj.com/site/about/guidelines.xhtml

Introduction

In Sweden as in many other countries, policies and laws emanating from patient needs regulate and define standards of healthcare[1]. Good healthcare is defined as safe, accessible, delivered in time, health promoting and knowledge- and evidence-based. In addition, to be fully efficient, good healthcare must be equitable. That is, on equal terms and according to patients' needs. On this background, The Swedish National Board of Health and Welfare (NBHW) and the Swedish Association of Local Authorities and Regions (SALAR) define quality indicators[2] that can be compared against specific benchmarks. These quality indicators are regularly monitored for performance assessment by means of simplified hospital and regional comparisons online (https://vardenisiffror.se/), and in reports such as *Open comparisons of healthcare performance*[3]. The benefits of monitoring for improving healthcare quality are possibly many[4]. However, current comparisons could be improved for at least two reasons we specify below.

First, in spite of the current evidence on the existence of healthcare inequities conditioned by sociodemographic factors (e.g., age, sex, income, country of birth), today's official monitoring is mostly focused on geographical disparities, disregarding other relevant axes of possible inequity. Monitoring both geographic and sociodemographic inequalities seem necessary in order to improve the evaluation of healthcare performance[5] .

For peer review only - http://bmjopen.bmj.com/site/about/guidelines.xhtml

Second, comparisons are often based on the analysis and interpretation of differences between group averages, disregarding individual heterogeneity around such averages. Furthermore, the criteria for quantifying the size of the group differences in these comparisons are not clearly stated. For instance, when can we say that there are large regional differences in a quality indicator? The criterion of "statistical significance" is not sufficient as very small and meaningless differences between group averages may nonetheless prove statistically significant if the sample is large enough. Also, measurable differences between group averages may exist together with considerable overlap of individual heterogeneity between groups, which may render irrelevant the average differences (See elsewhere for an extended explanation of this concept[6 7]).

To fill these deficiencies, in the current study we illustrate an innovative analytical framework that is being implemented in public health and health care epidemiology[5 6 8-18]. In the Analysis of Individual Heterogeneity and Discriminatory Accuracy (AIHDA)[8] we do not consider differences between group averages and differences between patients as if they were two separate and unrelated phenomena of interest. Rather, we adopt a multilevel perspective that simultaneously considers both types of differences. From this standpoint, we evaluate sociodemographic and geographical group differences by quantifying the share of the total individual variation in patient outcomes that operates at the group level. In the case of binary patient outcomes (like most quality indicators), we can analogously evaluate the accuracy of the group level for discriminating individuals

For peer review only - http://bmjopen.bmj.com/site/about/guidelines.xhtml
For peer review only

with the outcome from those without the outcome of interest. Applying AIHDA, we can evaluate the relevance of specific group differences (e.g., regional differences), and even compare the relative relevance of different grouping criteria (e.g., sociodemographic strata versus geographical regions). AIHDA can be applied using traditional regression[13 14] or more advanced multilevel models[5 6 8 11]. While we primarily recommended the use of multilevel models, for simplified routine monitoring and online presentations, traditional regression analysis provides a valid alternative.

Motivated by the above background, we aim to illustrate how the AIHDA approach can be applied in official online comparisons and reports. For doing so we analyze statin dispensation after acute myocardial infarction (AMI), which is a well-known process indicator specifically adopted by the Swedish NBHW[19]. In addition to geographical differences[4], research has shown sociodemographic inequalities in statin treatment among AMI patients, with less dispensations in women[20], immigrants [21], elderly people [22] and in patients with low socioeconomic position[23].

In summary, the aim of this study is to illustrate the use of AIHDA for public comparisons of sociodemographic and geographical differences in statin treatment after AMI hospitalization. For this purpose, we analyze the statins dispensations of 31,027 patients discharged from the hospital with an AMI diagnosis between January 2011 and December 2013.

For peer review only - http://bmjopen.bmj.com/site/about/guidelines.xhtml

Population and methods

Databases

Our investigation is based on a record-linkage database of several registers with national coverage, including the Swedish Population Register (TPR) and the Longitudinal Integration Database for Health Insurance and Labour Market Studies (LISA), administrated by Statistics Sweden (SCB), as well as the National Patient Register (NPR), the Cause of Death Register, and the Swedish Prescribed Drug Register (SPDR) administrated by the NBHW. The NPR records both outpatient clinic and inpatient discharges from hospital with diagnoses coded according to the International Classification of Diseases, 10th edition (ICD-10). However, it does not cover information on diagnoses in primary health care. The SPDR contains information on all prescription drug dispensations from Swedish pharmacies. The registers were linked using an arbitrary serial number assigned to each individual by statistics Sweden instead of the Swedish unique personal identification number to maintain confidentiality.

The Regional Ethics Review Board in Southern Sweden (ID 2014/856) and the data safety committees from the NBHW (ID 9542/2015) and from Statistics Sweden (ID 231424/878144-5) approved the construction of the database.

For peer review only - http://bmjopen.bmj.com/site/about/guidelines.xhtml

Study population

We defined the study population using the same criteria as applied by the Swedish NBHW for evaluating the quality indicator on statins dispensation after hospital discharge for AMI.

The initial population included all 40,468 patients aged 40-80 years, who were residing in Sweden by 31 December 2010, and were discharged from hospital with an *AMI diagnosis* (ICD-10: I21 or I22) between 1 January 2011 and 31 December 2013. We followed each patient between the 13th and 18th month after discharge from the index AMI hospitalization regarding their statin dispensation from the pharmacies. We excluded 4,925 patients who died between discharge from hospital and end of follow-up,149 patients who emigrated, and 350 patients with missing information on country of birth. The final study population included 35,044 patients. See figure 1 for details.

Assessment of variables

We defined the *quality indicator* according to the Swedish authorities as at least 90% of patients aged 40-80 years with a hospital discharge diagnosis of AMI and as having had at least one statin dispensation between the 13th and 18th month period after the hospital discharge[19]. We define *statin use* as any recorded dispensation of statin or a statin-combination drug (ATC codes C10AA or C10BA) in the SPDR during the follow-up period.

For peer review only - http://bmjopen.bmj.com/site/about/guidelines.xhtml

We classified the patients into the 21 *administrative regions* of Sweden according to their legal residence address and used Region Skåne as the reference region in the comparisons. Administrative regions in Sweden form autonomous political units and act as principals for healthcare.

We categorized *age* into two groups: 40-64 years and 65-80. *Sex* was defined as male or female according to the registered information. We categorized the patients according to their *country of birth* into native (i.e., born in Sweden) or not (i.e., immigrant).

We obtained information on *individualised disposable family income* for the years 2000, 2005 and 2010 to compute a cumulative income measure that considers the size of the household and the consumption weight of the individuals according to Statistics Sweden. For each of the three years, income levels were categorised into 25 quantiles using the complete Swedish population. These groups from the respective three years were summed up, so that each individual received a value between 3 (always in the lowest income group) and 75 (always in the highest income group). We categorised this cumulative income measure into three groups by tertiles (low, medium, or high income). Individuals with missing values on income during 2000 or 2005 were assigned the tertile values for the year 2010. No individuals had missing income data for 2010. By calculating a cumulative income measure, we obtain a more stable measurement of socioeconomic position.

For peer review only - http://bmjopen.bmj.com/site/about/guidelines.xhtml

Finally, we constructed a *sociodemographic multicategorical variable* consisting of all possible combinations of the categories of the four sociodemographic variables age, sex, income, and immigration status and thus resulting in 24 strata.

Patient and public involvement

Because of the nature of our study, patient and public involvement is necessary. We will communicate the results of our study to politicians and healthcare professionals, who represent the public's interest.

Statistical and epidemiological analysis

To examine the association between geographical and sociodemographic variables and the use of statins, we calculated the crude percentage of patients who were dispensed statins in each county and in each multicategorical sociodemographic strata.

Thereafter, we constructed three regressions models following the AIHDA approach. Since the prevalence of the outcome was not extreme, we measured the associations between use of statins and the explanatory variables by prevalence ratios (PRs) and their 95% confidence intervals (CI) rather than by odds ratios. For this purpose, we applied Cox proportional hazards regression models with a constant follow-up time equal to 1 and report robust confidence intervals [24].

For peer review only - http://bmjopen.bmj.com/site/about/guidelines.xhtml

Model 1A estimates geographical inequalities by including only the geographical variable with the 21 regions, specifying Region Skåne as reference in the comparisons.

Model 1B estimates sociodemographic inequalities by including the sociodemographic multicategorical variable only, specifying the strata of native 40-64 years old men as the reference category.

Model 2 enters the geographical variable and the sociodemographic multicategorical variable simultaneously. This model estimates the geographical and sociodemographic differences adjusted for each other

In order to evaluate the relevance of the sociodemographic versus geographical information for understanding use of statins after AMI, we estimated the Discriminatory accuracy (DA) for each model by calculating the area under the receiver operating characteristic curve (AUC) with 95% confidence intervals (CIs). The AUC is obtained by plotting the true-positive fraction against the false-positive fraction for different thresholds of the predicted probability of statin use. The value of the AUC ranges from .5 to 1, with .5 indicating no predictive accuracy and 1 representing perfect discrimination. Using the criteria proposed by Hosmer and Lemeshow [25], we classified DA as absent or very small (.5 ≤ AUC ≤ .6), small (.6 < AUC ≤ .7), large (.7 < AUC ≤ 0.8), or very large (AUC > .8). As explained in previous publications [6 7], measuring geographical and sociodemographic differences to evaluate healthcare quality is analogous to performing a

For peer review only - http://bmjopen.bmj.com/site/about/guidelines.xhtml

diagnostic test [26]. No decision-maker in healthcare would want to use such a test without first knowing its discriminatory accuracy (i.e., sensitivity, specificity, AUC). We do not want to unnecessarily treat false positive individuals or incorrectly deny treatment of false negative patients. In our case, we want to know the accuracy of public sociodemographic and geographical comparisons for discriminating individuals with a positive result of the quality indicator (i.e., use of statins) from those with a negative result (i.e., no statins).

Strategy for evaluating sociodemographic and geographical differences

Applying the AIHDA framework means that we focus on both the group averages (region and sociodemographic strata averages) and the individual heterogeneity around these averages. This framework includes four steps to achieve a complete analysis of inequalities. However, the approach may be modified in different research questions[6].

In the *first step (i)* we identify the quality indicator and the target or benchmark average value as we described above. In the *second step (ii)* we estimate and visualize the groups' differences using appropriate plots (e.g., caterpillar plot) and in the *third step (iii)* we quantify the size of the differences. That is, for a correct interpretation of the plots, the size of the groups' differences needs to be evaluated using a measure of discriminatory accuracy like the AUC. In traditional comparisons of group averages, the criteria for quantifying the size of the geographical and sociodemographic differences are not clearly stated. However, in the AIHDA framework we evaluate geographical and sociodemographic differences by quantifying the share of the total

For peer review only - http://bmjopen.bmj.com/site/about/guidelines.xhtml

variation in individual outcomes that operates at the group level (i.e., regions, sociodemographic strata). In the case of binary individual health outcomes, we can use the AUC as it provides information expressed in terms of discriminatory accuracy. Finally, in the *fourth step (iv)* we proceed to the interpretation of the results. Following the study referred earlier [6], we present a practical framework for evaluating geographical and sociodemographic differences. For this purpose, we need at least two types of information.

First, we need to know whether the average quality indicator (i.e., percentage of patients on statins after AMI) has reached the predetermined target value fully (i.e., ≥ 90% of all AMI patients are on statins), closely (i.e., 80-89%), or insufficiently (i.e., < 80%). The criteria we use to qualify the prevalence value as “fully” is based on the definition given by Swedish NBHW. However, the qualifications "closely" and “insufficiently” are subjective and may differ across quality indicators.

Second, we need information on the *size of the group differences* as assessed by the AUC, which expresses the accuracy of the differences between group averages for discriminating patients with the outcome from those without the outcome. These two types of information need to be combined for the evaluation. Box 1 illustrates these ideas by means of a simple framework with 12 scenarios that can be used to orient the interpretation of an analysis.

Box 1. Framework for evaluating geographical and sociodemographic differences in a specific

For peer review only - http://bmjopen.bmj.com/site/about/guidelines.xhtml

quality indicator. For this purpose, we need information about the *target indicator value*. That is, whether the average quality indicator (i.e., percentage of patients on statins after AMI) has "insufficiently", "closely" or "fully" reached a predetermined target *benchmark* level. We also need information on the area under receiver operating characteristics curve (AUC), which expresses the accuracy of the differences between group averages for discriminating patients with the outcome from those without the outcome. Combining this information, we obtain 12 different scenarios useful for the evaluation.

		**Target indicator value reached**		
**Size of the group differences**		Insufficiently	Closely	Fully
Absent/very small	.5 ≤ AUC ≤ .6	A	B	C
Small	.6 ≤ AUC < .7	D	E	F
Large	.7 ≤ AUC < .8	G	H	I
Very large	.8 ≤ AUC	J	K	L

In the ideal scenario (scenario C) the desired target level has been fully reached in the population, and the group differences are absent or very small. The conclusion would be that all groups have been treated appropriately and on equal terms. From this perspective, the quality of the healthcare is high.

In the worst scenario (scenario A) the desired target level has not been achieved in the population, and the groups differences are again absent. The conclusion would be that all groups have been treated on equal terms but inadequately. That is, the quality of healthcare in the whole population is low.

Observe that in both scenarios A and C interventions targeted to specific groups are not justifiable. Rather any intervention should be universal (i.e., directed to the whole

For peer review only - http://bmjopen.bmj.com/site/about/guidelines.xhtml

country). In scenario C, the reason for the intervention would be to maintain the desirable value of the quality indicator in all groups. In the scenario A, the reason would be to reach this desirable level in all the groups.

The interpretation of the scenarios in the lowest corners of the table (J and L) is conditioned by the very large size of the group differences. For example, in scenario L some groups may not have achieved the target level even if the population as a whole has done so. In contrast, in scenario J, some groups may have achieved the target level even if the population as a whole has not achieved it. In these scenarios (J and L) targeted group interventions are justified.

The framework we propose fits well with the concept of *proportionate universalism* for resource allocation in public health [27]. That is, health care actions must be universal, not targeted, but with a scale and intensity that is proportionate to the level of disadvantage. The AIHDA approach we apply in this study can be used to inform decisions regarding the appropriate scale and intensity for a given sociodemographic or geographic context.

Results

The overall prevalence of statin dispensations in AMI patients 12-18 months after discharge was 83.7% (29,231/35,044). Therefore, the benchmark value of 90% was *closely* but not fully met on a national level according to the framework in Box 1.

For peer review only - http://bmjopen.bmj.com/site/about/guidelines.xhtml

Figure 2 and Table 1 illustrate the prevalence of statin dispensations in each administrative region in Sweden. Västmanland has the highest prevalence of statin use at 87.4% while Gotland has the lowest prevalence at 76.4%. None of the regions reach the benchmark value of 90%. The relative differences when comparing with the region of Skåne (Model 1A) were tiny and Table 1 illustrates that only four regions (i.e., Gotland, Västernorrland, Stockholm and Västra Götaland) presented a slightly but conclusively lower PR than Skåne. No other region showed a conclusively higher PR than Skåne. The PR adjusted for the multicategorical sociodemographic variable (Model 2) were very similar to the unadjusted PR (Model 1A), indicating that the sociodemographic composition of the regions did not confound the geographical differences.

Table 1. Number of patients using statins (n), number of individuals (N), crude prevalence (P) and 95% confidence intervals (CI), unadjusted prevalence ratios (PR), and prevalence ratios adjusted for sociodemographic variables (PRa) for use of statins 12 -18 months after hospital discharge with an acute myocardial infarction (AMI) diagnosis in patients aged 40-80 years from the 21 Swedish geographical administrative regions in the period 2011-2013.

				Model 1A	Model 2
	n	N	P (95% CI) %	PR (95% CI)	Pra (95% CI)
Sweden	29321	35044	83.67 (83.28-84.06)	–	–
Administrative Regions					
Gotland	246	322	76.40 (71.76-81.04)	0.90 (0.85-0.96)	0.91 (0.85-0.97)
Västernorrland	922	1153	79.97 (77.65-82.28)	0.95 (0.92-0.98)	0.94 (0.92-0.97)
Örebro	886	1082	81.89 (79.59-84.18)	0.97 (0.94-1.00)	0.97 (0.94-1.00)
Blekinge	525	639	82.16 (79.19-85.13)	0.97 (0.93-1.01)	0.98 (0.94-1.02)
Stockholm	4477	5440	82.30 (81.28-83.31)	0.97 (0.96-0.99)	0.97 (0.95-0.98)
Västra Götaland	4391	5326	82.44 (81.42-83.47)	0.97 (0.96-0.99)	0.97 (0.96-0.99)
Uppsala	960	1164	82.47 (80.29-84.66)	0.97 (0.95-1.00)	0.97 (0.94-1.00)
Norrbotten	1215	1465	82.94 (81.01-84.86)	0.98 (0.95-1.01)	0.98 (0.95-1.00)
Östergötland	1400	1682	83.23 (81.45-85.02)	0.98 (0.96-1.01)	0.98 (0.96-1.01)

For peer review only - http://bmjopen.bmj.com/site/about/guidelines.xhtml

Dalarna	1212	1444	83.93 (82.04-85.83)	0.99 (0.97-1.02)	0.99 (0.97-1.02)
Jämtland	501	595	84.20 (81.27-87.13)	1.00 (0.96-1.03)	1.00 (0.96-1.03)
Västerbotten	893	1058	84.40 (82.22-86.59)	1.00 (0.97-1.03)	0.99 (0.97-1.02)
Halland	940	1112	84.53 (82.41-86.66)	1.00 (0.97-1.03)	1.00 (0.97-1.02)
Skåne	4085	4828	84.61 (83.59-85.63)	Reference	Reference
Kalmar	898	1054	85.20 (83.06-87.34)	1.01 (0.98-1.04)	1.01 (0.98-1.04)
Södermanland	973	1141	85.28 (83.22-87.33)	1.01 (0.98-1.04)	1.01 (0.98-1.04)
Gävleborg	1126	1318	85.43 (83.53-87.34)	1.01 (0.98-1.04)	1.01 (0.99-1.04)
Kronoberg	592	685	86.42 (83.86-88.99)	1.02 (0.99-1.05)	1.02 (0.99-1.05)
Värmland	1076	1240	86.77 (84.89-88.66)	1.03 (1.00-1.05)	1.03 (1.00-1.05)
Jönköping	1126	1292	87.15 (85.33-88.98)	1.03 (1.01-1.06)	1.03 (1.00-1.05)
Västmanland	877	1004	87.35 (85.29-89.41)	1.03 (1.01-1.06)	1.04 (1.01-1.06)
Area under the ROC curve (AUC)				0.537	0.618

Figure 3 and Table 2 illustrate the crude prevalence of statin dispensations for each sociodemographic stratum, with the highest prevalence (i.e., 89.5%) in the reference strata of 40–64-year-old native men with middle income, followed by the strata of 40–64-year-old native men with middle income (i.e., 88.8%). We found the lowest prevalence of statin use in the stratum of 65–80-year-old immigrant women with high income (i.e., 70.3%), closely followed by 65–80-year-old immigrant women with low income (i.e., 74.1%). Only the reference sociodemographic stratum almost grasped the target value of 90% (i.e., 89.5%). All the women strata reached the target insufficiently and all the men strata reached the target closely. Table 2 also presents the unadjusted PR (Model 1B), and the PR adjusted for the geographical variable (Model 2), with the strata of 40–64-year-old high-income native men as reference stratum. As many as 19 sociodemographic strata show conclusively smaller PRs than the reference. The

For peer review only - http://bmjopen.bmj.com/site/about/guidelines.xhtml

adjusted PR were very similar to the unadjusted PR indicating that geographical factors did not confound the sociodemographic differences.

Table 2. Number of patients using statins (n), number of individuals (N), crude prevalence (P) and 95% confidence intervals (CI), unadjusted prevalence ratios (PR), and prevalence ratios adjusted for administrative regions (PRa) for use of statins 12-18 months after hospital discharge with an acute myocardial infarction (AMI) diagnosis in patients aged, 40-80 years old from 24 sociodemographic strata in the period 2011-2013

				Model 1B	Model 2
	n	N	P (95% CI) %	PR (95% CI)	PRa (95% CI)
Sweden	29,321	35,044	83.67 (83.28-84.06)	–	–
Women					
65-80 , High income, Immigrant	180	256	70.31 (64.72-75.91)	0.79 (0.72-0.85)	0.79 (0.73-0.85)
65-80 , Low income, Immigrant	300	405	74.07 (69.81-78.34)	0.83 (0.78-0.88)	0.83 (0.78-0.88)
65-80 , Middle income, Native	1936	2575	75.18 (73.52-76.85)	0.84 (0.82-0.86)	0.84 (0.82-0.86)
40-64 , Low income, Native	479	637	75.20 (71.84-78.55)	0.84 (0.80-0.88)	0.84 (0.80-0.88)
65-80 , Low income, Native	852	1132	75.27 (72.75-77.78)	0.84 (0.81-0.87)	0.84 (0.81-0.87)
65-80 , Middle income, Immigrant	338	448	75.45 (71.46-79.43)	0.84 (0.80-0.89)	0.84 (0.80-0.89)
40-64 , Middle income, Immigrant	130	171	76.02 (69.62-82.42)	0.85 (0.78-0.92)	0.85 (0.78-0.93)
40-64 , Low income, Immigrant	197	257	76.65 (71.48-81.83)	0.86 (0.80-0.92)	0.86 (0.80-0.92)
40-64 , Middle income, Native	656	846	77.54 (74.73-80.35)	0.87 (0.83-0.90)	0.86 (0.83-0.90)
65-80 , High income, Native	1838	2369	77.59 (75.91-79.26)	0.87 (0.85-0.89)	0.87 (0.85-0.89)
40-64 , High income, Immigrant	92	118	77.97 (70.49-85.44)	0.87 (0.79-0.96)	0.87 (0.79-0.96)
40-64 , High income, Native	709	904	78.43 (75.75-81.11)	0.88 (0.85-0.91)	0.87 (0.84-0.91)
Men					
65-80 , Low income, Immigrant	548	679	80.71 (77.74-83.67)	0.90 (0.87-0.94)	0.90 (0.87-0.94)
65-80 , Low income, Native	1409	1735	81.21 (79.37-83.05)	0.91 (0.88-0.93)	0.91 (0.88-0.93)
65-80 , Middle income, Immigrant	565	686	82.36 (79.51-85.21)	0.92 (0.89-0.95)	0.92 (0.89-0.95)
40-64 , Low income, Native	1283	1540	83.31 (81.45-85.17)	0.93 (0.91-0.95)	0.93 (0.91-0.95)
40-64 , Middle income, Immigrant	397	472	84.11 (80.81-87.41)	0.94 (0.90-0.98)	0.94 (0.90-0.98)
65-80 , Middle income, Native	3791	4458	85.04 (85.04-85.04)	0.95 (0.93-0.97)	0.95 (0.93-0.96)
40-64 , Low income, Immigrant	1006	1171	85.91 (83.92-87.90)	0.96 (0.94-0.98)	0.96 (0.94-0.99)
65-80 , High income, Immigrant	570	645	88.37 (88.37-88.37)	0.99 (0.96-1.02)	0.99 (0.96-1.02)
40-64 , High income, Immigrant	337	381	88.45 (85.24-91.66)	0.99 (0.95-1.03)	0.99 (0.95-1.03)
65-80 , High income, Native	6303	7101	88.76 (88.76-88.76)	0.99 (0.98-1.01)	0.99 (0.98-1.00)
40-64 , Middle income, Native	2240	2522	88.82 (87.59-90.05)	0.99 (0.97-1.01)	0.99 (0.97-1.01)
40-64 , High income, Native	3165	3536	89.51 (88.50-90.52)	Reference	Reference

For peer review only - http://bmjopen.bmj.com/site/about/guidelines.xhtml

For peer review only

Area under the ROC curve (AUC)	0.609	0.618

Model 1A, which includes information about administrative regions, presented an AUC value of 0.537. This AUC value indicates that the geographical differences were *absent/very small,* and that region provides very limited information about statin dispensations. Model 1B, which includes information on the sociodemographic strata, showed an AUC of 0.609 which indicates that the sociodemographic differences were *small*. The sociodemographic AUC was 0.072 units higher than the AUC of the geographical model 1A. Model 2, which includes both geographical and sociodemographic information, presented an AUC value of 0.618, just 0.008 higher than that presented by model 1B. In the context of our analysis, these findings indicates that sociodemographic information is relatively more relevant than geographic information. In summary, the target indicator value was *closely* reached in Sweden as a whole. The geographical analysis indicated *absent/very small* differences, i.e., scenario B in the Box 1. The sociodemographic analysis indicated *small* differences, i.e., scenario E in the Box 1.

To understand the low AUC values of the geographical model, in Table 1 we can, e.g., estimate that of the total 5723 AMI patients without statin dispensations from the 21 regions, more than half (n= 3004) were in the 14 regions with the highest prevalence of statin dispensation. Similarly, in Table 2 we can estimate that the number of male AMI patients without statins (n = 3312) was much higher than the number of female AMI

For peer review only - http://bmjopen.bmj.com/site/about/guidelines.xhtml

patients without statins (n = 2411) even though women consistently have a lower prevalence and a lower PR of statin dispensation than men. This reflects that while the prevalence of using statins is higher among men, the fact that AMI patients are disproportionately male means that majority of non-statin users are nevertheless also men.

Discussion

We evaluated regional and sociodemographic inequalities in statin treatment after AMI. When doing so, we also aimed to demonstrate how AIHDA provides enhanced information for evaluating healthcare inequalities [6 28]. While traditional public comparisons focus on differences between group averages, AIHDA considers both differences between group averages and the individual heterogeneity around those averages. AIHDA informs on the share of the total individual variation in patient outcomes that operates at the group level. That is, to which degree the group-level information is able to discriminate with accuracy patients with, from those without the indicator under study[6].

The overall prevalence of statin treatment in our study population was 83.7% which is close to, but lower than the target benchmark value of 90%. We observed regional and sociodemographic differences in the prevalence of statin treatment after AMI. However, the low AUC values indicated that the differences were “absent/very small” (AUC =

For peer review only - http://bmjopen.bmj.com/site/about/guidelines.xhtml

0.537) for the administrative regions and "small" (AUC = 0.609) for the sociodemographic groups. Combining both regional and sociodemographic information did not substantially increase the AUC of the sociodemographic information alone (AUC = 0.618). That is, the sociodemographic differences seem to play a modest but more relevant role than the regional differences for understanding inequalities in statin treatment after AMI. Our analysis also shows that women in all strata have a lower prevalence of statin dispensations after AMI than men, which is in line with previous research indicating the existence of gender inequalities [20]. However, those inequalities need be interpreted in the light of the small DA of the sociodemographic strata.

According to the framework presented in box 1, the sociodemographic differences would be placed in scenario E. That is, the target value was closely but not fully reached and there were small differences between the sociodemographic strata. Accordingly, any strategy aimed to improve the outcome of the quality indicator should be universal but proportionately more intense among sociodemographic groups with increased risk not using statins. Analogously, the differences between administrative regions would be placed in the scenario B. In this case we should recommend universal interventions without targeting any specific regions.

Traditional studies exclusively based on differences between group averages implicate several disadvantages as compared to AIHDA. It is known that epidemiological studies comparing group averages may *stigmatize* the individuals belonging to groups with "bad"

For peer review only - http://bmjopen.bmj.com/site/about/guidelines.xhtml

average values and create *false expectations* for the individuals belonging to groups with "good" average values. This situation is true for both traditional and AIHDA studies and it is an inevitable side effect in Epidemiology. Nevertheless, the perils of stigmatization and false expectations are often ethically justifiable because the aim of the epidemiological analyses is to improve the health and heath care of the individuals. However, knowing the DA of the group comparisons allows us to decide whether pointing out specific population groups is justifiable or not. If the DA is high, pointing out specific population groups is justifiable. However, if the DA is low, the traditional emphasis on differences between group averages should be avoided. For instance, our results show that, because the AUC value is low, differences between administrative regions are not relevant for understanding individual statin dispensations. Therefore, simplistic interpretation based on visual analyses of figures like the caterpillar plot in our study (Figure 2) should be sidestepped.

By overlooking the DA of the group-averages, traditional studies may simultaneously lead to both under and overtreatment if a possible intervention is based on group information only. If the DA is low, many individuals taking statins but belonging to a group with a "bad" average (i.e., false positives cases) could be exposed to the intervention while many individuals not taking statins but belonging to a group with a "good" average (i.e., false negative cases) would not receive any intervention. For instance, using the information in Table 1, we can see that the region of Gotland has the worst average

For peer review only - http://bmjopen.bmj.com/site/about/guidelines.xhtml

value (i.e., 76.4%), meaning that 76 of the 322 patients were not dispensed statins. However, in the region of Västmanland with the best average value (i.e., 87.4 %), this figure was 127 of the 1004 patients since Västmanland has a larger population. That is, the “best” region has 1.7 (= 127/76) times more patients lacking statin treatment that the “worst” region. It is correct that the region of Västmanland has a higher proportion of treated patients but from a national perspective the burden of untreated patients is also higher in the region of Västmanland. Therefore, if the DA is low, any potential intervention to increase statin treatment after AMI needs to be universal (i.e., on the whole country) and directed to all AMI patients no matter the region they belong to.

Strengths and limitations

One strength of this study is the quality of the data since all information is based on national registers where the information is recorded in a highly standardized way for both ICD and ATC codes. The validity of the AMI diagnosis as recorded in the NPR is high [29] and the sociodemographic information is recorded by Statistics Sweden with a longstanding experience in the field [https://www.scb.se/en/About-us/main-activity/quality-work/]. Moreover, we can directly compare our results to those presented by the NBHW since the definition of the quality indicator is the same. Another methodological strength in this study is the incorporation of a measurement of DA, which allows assessing differences between geographical and sociodemographic categories.

For peer review only - http://bmjopen.bmj.com/site/about/guidelines.xhtml

One limitation of this study is that we excluded the 5,424 individuals who died, emigrated, or had missing information on country of birth which corresponds to approximately 13% of the final study population, and it cannot be ruled out that this exclusion introduces a selection bias. However, we do not think that this limitation alters our conclusions as the selection criteria are in concordance with the those used by the Swedish authorities. Another limitation in this study could be the use of household income as a proxy for socioeconomic position, which does not capture other financial assets, nor necessarily higher education.

The definition of country of birth within this study could be elaborated with more refined categories, other than native and immigrant. Immigrant is a broad concept which includes, among other factors, cultural, economic, and educational heritages from another countries. However, more categories of country of birth would result in a larger number of multicategorical strata with a smaller number of within strata observations. An analogous reasoning is applicable for the age categories. There is a possible underestimation of the impact of age since we only use two categories in our analyses.

The AIHDA approach adopts a multilevel perspective that conceptually partitions the individual differences into within groups (level 1) and between groups (level 2). Therefore, it would be appropriate to denominate the approach as Multilevel AIHDA or "MAIHDA". From the statistical point of view, the AIHDA approach can be implemented using both traditional regression analyses with the group as fixed effects [10 12 14 30] or more

For peer review only - http://bmjopen.bmj.com/site/about/guidelines.xhtml

advanced multilevel regression analyses (MLRA) with a random effect for the groups [5 6 8 9 11 13 15-17]. MLRA provide conceptual and methodological advantages as compared to traditional fixed effects analyses[31]. For instance, both fixed and random effects analyses considers the groups as contexts but in the traditional fixed effects analyses all information is modelled at the first level (individual) while in MLRA the groups are explicitly modeled as a second level. In MLRA the relative differences (e.g., PR) have the grand mean (i.e., the mean of the groups means) as reference while in traditional analyses the reference is a specific group (as in our present study). MLRA can better handle groups with few individuals via providing reliability weighted estimations. In addition to the AUC for assessing the importance of the groupeffects [32], MLRA also provides the variance partition coefficient (VPC) [28 33]. The VPC quantifies the proportion of outcome variation that lies between groups. Nevertheless, in order to most reach the widest possible audience, in the current study we aimed to demonstrate the use traditional fixed effects regression within the AIHDA framework. In doing so, we hope to maximally increase the understanding and the application of AIHDA in current epidemiological practice aimed to the quantitative evaluation of health care quality and public comparison in health care [3 7].

Implications and Conclusions

Dispensations of statins after AMI in Sweden are, according to the national quality indicator, closely but not fully met in relation to the benchmark value of 90%. However,

For peer review only - http://bmjopen.bmj.com/site/about/guidelines.xhtml

neither regional nor sociodemographic differences in group averages seem to be major determinants of dispensation of statins. Therefore, any intervention aiming to reach the benchmark value should be universal. Sociodemographic differences nevertheless appeared more relevant than geographic differences and our results suggest that a special emphasis should be directed to women, especially to those with immigrant background and older than 65 years.

A regular and public monitoring of geographic and sociodemographic inequalities in quality indicators is fundamental for the evaluation of health care quality. For this purpose, we need to apply state of the art statistical and epidemiological methods. The traditional mean-centric approach based on the analysis of differences between groups averages provides incomplete information that might mislead decision makers in healthcare. As illustrated in our study, AIHDA is not complicated to implement, and it provides more nuanced information than traditional public comparisons based only on group averages. We therefore propose the application of the AIHDA framework for the quantitative evaluation of healthcare quality when performing geographic and sociodemographic comparisons of quality indicators.

Footnotes

- **Contributors** JM had the initiative of the study and acquired the data. JM and JÖ wrote the original manuscript. RP-V and JÖ performed the analyses in coordination with JM, KK and GL. All the authors have contributed to the design of the study, JM and GL developed the methodology and directed the analyses. All

For peer review only - http://bmjopen.bmj.com/site/about/guidelines.xhtml

authors have contributed to the interpretation of the results and drafting of the manuscript. All authors have revised the last version of the manuscript.

- **Funding** The Swedish Research Council (Vetenskapsrådet) supported this work through the project 'Multilevel Analyses of Individual Heterogeneity: innovative concepts and methodological approaches in Public Health and Social Epidemiology': (#2017-01321, PI: Juan Merlo).
- **Competing interests** None declared.
- **Patient consent for publication** Not required.
- **Provenance and peer review** Not commissioned; externally peer reviewed.
- **Data availability statement** Data are available upon reasonable request. Original data are available from the Swedish National Board of Health and Welfare and Statistics Sweden, after approval of the research project by an Ethical Committee and by the data safety committees of Swedish Authorities
- **Ethics approval** Ethics approval for research involving human participants was given by Regional ethics committee Lund (ID 2014/856). Informed consent was not necessary according to the Regional ethics committee because of the nature of the register study. The database was approved by NBHW (ID 9542/2015) and by Statistics Sweden (ID 231424/878144-5).

For peer review only - http://bmjopen.bmj.com/site/about/guidelines.xhtml

References

1. Socialstyrelsen. God vård – om ledningssystem för kvalitet och patientsäkerhet i hälso- och sjukvården. In: Welfare SBoHa, ed., 2006:ISBN: 91-85482-05-6.
2. Heurgren M, Åberg A, Köster M, et al. Performance indicators in Swedish Health Care. *BMC Health Serv Res* 2007;7(A10) doi: ttps://doi.org/10.1186/1472-6963-7-S1-A10
3. Öppna jämförelser av hälso- och sjukvårdens kvalitet och effektivitet Jämförelser mellan landsting . *Sveriges Kommuner och Landsting wwwsklse/publikationer ISBN 978-91-7164-485-5* 2009
4. Sorman H. [Open comparisons of health care: "One of the most important contributions to modernization of health care"]. *Lakartidningen* 2010;107(32-33):1828-9. [published Online First: 2010/09/29]
5. Khalaf K, Axelsson Fisk S, Ekberg-Jansson A, et al. Geographical and sociodemographic differences in discontinuation of medication for Chronic Obstructive Pulmonary Disease - A Cross-Classified Multilevel Analysis of Individual Heterogeneity and Discriminatory Accuracy (MAIHDA). *Clin Epidemiol* 2020;12:783-96. doi: 10.2147/CLEP.S247368 [published Online First: 2020/08/09]
6. Merlo J, Wagner P, Leckie G. A simple multilevel approach for analysing geographical inequalities in public health reports: The case of municipality differences in obesity. *Health Place* 2019;58:102145. doi: 10.1016/j.healthplace.2019.102145 [published Online First: 2019/06/14]
7. Merlo J. Flernivåanalyser behövs i öppna jämförelser inom hälso- och sjukvård. *Läkartidningen 2019;116:FTEL* 2019
8. Merlo J. Multilevel analysis of individual heterogeneity and discriminatory accuracy (MAIHDA) within an intersectional framework. *Soc Sci Med* 2018;203:74-80. doi: 10.1016/j.socscimed.2017.12.026 [published Online First: 2018/01/07]
9. Evans CR, Erickson N. Intersectionality and depression in adolescence and early adulthood: A MAIHDA analysis of the national longitudinal study of adolescent to adult health, 1995-2008. *Soc Sci Med* 2019;220:1-11. doi: 10.1016/j.socscimed.2018.10.019
10. Mulinari S, Bredstrom A, Merlo J. Questioning the discriminatory accuracy of broad migrant categories in public health: self-rated health in Sweden. *Eur J Public Health* 2015;25(6):911-7. doi: 10.1093/eurpub/ckv099
11. Ljungman H, Wemrell M, Khalaf K, et al. Antidepressant use in Sweden: an intersectional multilevel analysis of individual heterogeneity and discriminatory accuracy (MAIHDA). *Scand J Public Health* 2021:1403494821993723. doi: 10.1177/1403494821993723 [published Online First: 2021/02/24]
12. Axelsson Fisk S, Lindstrom M, Perez-Vicente R, et al. Understanding the complexity of socioeconomic disparities in smoking prevalence in Sweden: a cross-sectional study applying intersectionality theory. *BMJ Open* 2021;11(2):e042323. doi: 10.1136/bmjopen-2020-042323 [published Online First: 2021/02/13]
13. Axelsson Fisk S, Mulinari S, Wemrell M, et al. Chronic Obstructive Pulmonary Disease in Sweden: An intersectional multilevel analysis of individual heterogeneity and discriminatory accuracy. *SSM Popul Health* 2018;4:334-46. doi: 10.1016/j.ssmph.2018.03.005
14. Wemrell M, Bennet L, Merlo J. Understanding the complexity of socioeconomic disparities in type 2 diabetes risk: a study of 4.3 million people in Sweden. *BMJ Open Diabetes Res Care* 2019;7(1):e000749. doi: 10.1136/bmjdrc-2019-000749 [published Online First: 2019/12/05]
15. Hernandez-Yumar A, Wemrell M, Abasolo Alesson I, et al. Socioeconomic differences in body mass index in Spain: An intersectional multilevel analysis of individual heterogeneity and discriminatory accuracy. *PLoS One* 2018;13(12):e0208624. doi: 10.1371/journal.pone.0208624
16. Persmark A, Wemrell M, Zettermark S, et al. Precision public health: Mapping socioeconomic disparities in opioid dispensations at Swedish pharmacies by Multilevel Analysis of Individual

For peer review only - http://bmjopen.bmj.com/site/about/guidelines.xhtml

Heterogeneity and Discriminatory Accuracy (MAIHDA). *PLoS One* 2019;14(8):e0220322. doi: 10.1371/journal.pone.0220322 [published Online First: 2019/08/28]

17. Rodriguez-Lopez M, Merlo J, Perez-Vicente R, et al. Cross-classified Multilevel Analysis of Individual Heterogeneity and Discriminatory Accuracy (MAIHDA) to evaluate hospital performance: the case of hospital differences in patient survival after acute myocardial infarction. *BMJ Open* 2020;10(10):e036130. doi: 10.1136/bmjopen-2019-036130 [published Online First: 2020/10/26]
18. Ghith N, Frolich A, Merlo J. The role of the clinical departments for understanding patient heterogeneity in one-year mortality after a diagnosis of heart failure: A multilevel analysis of individual heterogeneity for profiling provider outcomes. *PLoS One* 2017;12(12):e0189050. doi: 10.1371/journal.pone.0189050
19. Socialstyrelsen. Nationella riktlinjer för hjärtsjukvård Stöd för styrning och ledning. In: 2018-6-28| A, ed. Stockholm, Sweden, 2018.
20. Ruiz-Cantero MT, Blasco-Blasco M, Chilet-Rosell E, et al. Gender bias in therapeutic effort: from research to health care. *Farm Hosp* 2020;44(3):109-13. doi: 10.7399/fh.11394 [published Online First: 2020/05/27]
21. Chang AY, Abou-Arraj NE, Rodriguez F. Interventions to Reduce Ethnic and Racial Disparities in Dyslipidemia Management. *Current treatment options in cardiovascular medicine* 2019;21(5):24. doi: 10.1007/s11936-019-0725-4 [published Online First: 2019/05/09]
22. Wylie LE, Waterbrook AL, Dalen JE. Are Statins Indicated in Senior Citizens? A Review of Statin Therapy in the Elderly. *Am J Med* 2021 doi: 10.1016/j.amjmed.2021.11.009 [published Online First: 2021/12/03]
23. Hyun KK, Brieger D, Woodward M, et al. The effect of socioeconomic disadvantage on prescription of guideline-recommended medications for patients with acute coronary syndrome: systematic review and meta-analysis. *Int J Equity Health* 2017;16(1):162. doi: 10.1186/s12939-017-0658-z [published Online First: 2017/09/02]
24. Barros AJ, Hirakata VN. Alternatives for logistic regression in cross-sectional studies: an empirical comparison of models that directly estimate the prevalence ratio. *BMC Med Res Methodol* 2003;3:21. doi: 10.1186/1471-2288-3-21
25. Hosmer DW, Lemeshow S. Applied logistic regression. 2nd ed. New York: Wiley 2000.
26. Pepe M. S., Janes H., Longton G., et al. Limitations of the odds ratio in gauging the performance of a diagnostic, prognostic, or screening marker. *Am J Epidemiol* 2004;159(9):882-90. [published Online First: 2004/04/24]
27. Marmot M, Bell R. Fair society, healthy lives. *Public Health* 2012;126 Suppl 1:S4-S10. doi: 10.1016/j.puhe.2012.05.014 [published Online First: 2012/07/13]
28. Merlo J, Wagner P, Ghith N, et al. An Original Stepwise Multilevel Logistic Regression Analysis of Discriminatory Accuracy: The Case of Neighbourhoods and Health. *PLoS One* 2016;11(4):e0153778. https://doi.org/10.1371/journal.pone.78. doi: https://doi.org/10.1371/journal.pone.0153778
29. Ludvigsson JF, Andersson E, Ekbom A, et al. External review and validation of the Swedish national inpatient register. *BMC Public Health* 2011;11:450. doi: 10.1186/1471-2458-11-450
30. Wemrell M, Karlsson N, Perez Vicente R, et al. An intersectional analysis providing more precise information on inequities in self-rated health. *Int J Equity Health* 2021;20(1):54. doi: 10.1186/s12939-020-01368-0 [published Online First: 2021/02/05]
31. Evans CR, Leckie G, Merlo J. Multilevel versus single-level regression for the analysis of multilevel information: The case of quantitative intersectional analysis. *Soc Sci Med* 2020;245:112499. doi: 10.1016/j.socscimed.2019.112499 [published Online First: 2019/09/23]
32. Wagner P, Merlo J. Discriminatory accuracy of a random effect in multilevel logistic regression. *International Journal of Epidemiology* 2014;44:i49-i50.
33. Merlo J, Wagner P, Austin PC, et al. General and specific contextual effects in multilevel regression analyses and their paradoxical relationship: A conceptual tutorial. *SSM Popul Health* 2018;5:33-37. doi: 10.1016/j.ssmph.2018.05.006

For peer review only

For peer review only - http://bmjopen.bmj.com/site/about/guidelines.xhtml

For peer review only

For peer review only - http://bmjopen.bmj.com/site/about/guidelines.xhtml

Figure 1: Study population flow-chart

Figure 2: Crude prevalence (P) and 95% confidence intervals (CI) for use of statins 12 -18 months after hospital discharge with an acute myocardial infarction (AMI) diagnosis in patients aged 40-80 years in the period 2011-2013, across the 21 administrative regions of Sweden.

Figure 3: Crude prevalence (P) and 95% confidence intervals (CI) for use of statins 12 -18 months after hospital discharge with an acute myocardial infarction (AMI) diagnosis in patients aged 40-80 years in the period 2011-2013, across the 24 sociodemographic groups.

For peer review only

For peer review only - http://bmjopen.bmj.com/site/about/guidelines.xhtml

For peer review only

For peer review only - http://bmjopen.bmj.com/site/about/guidelines.xhtml

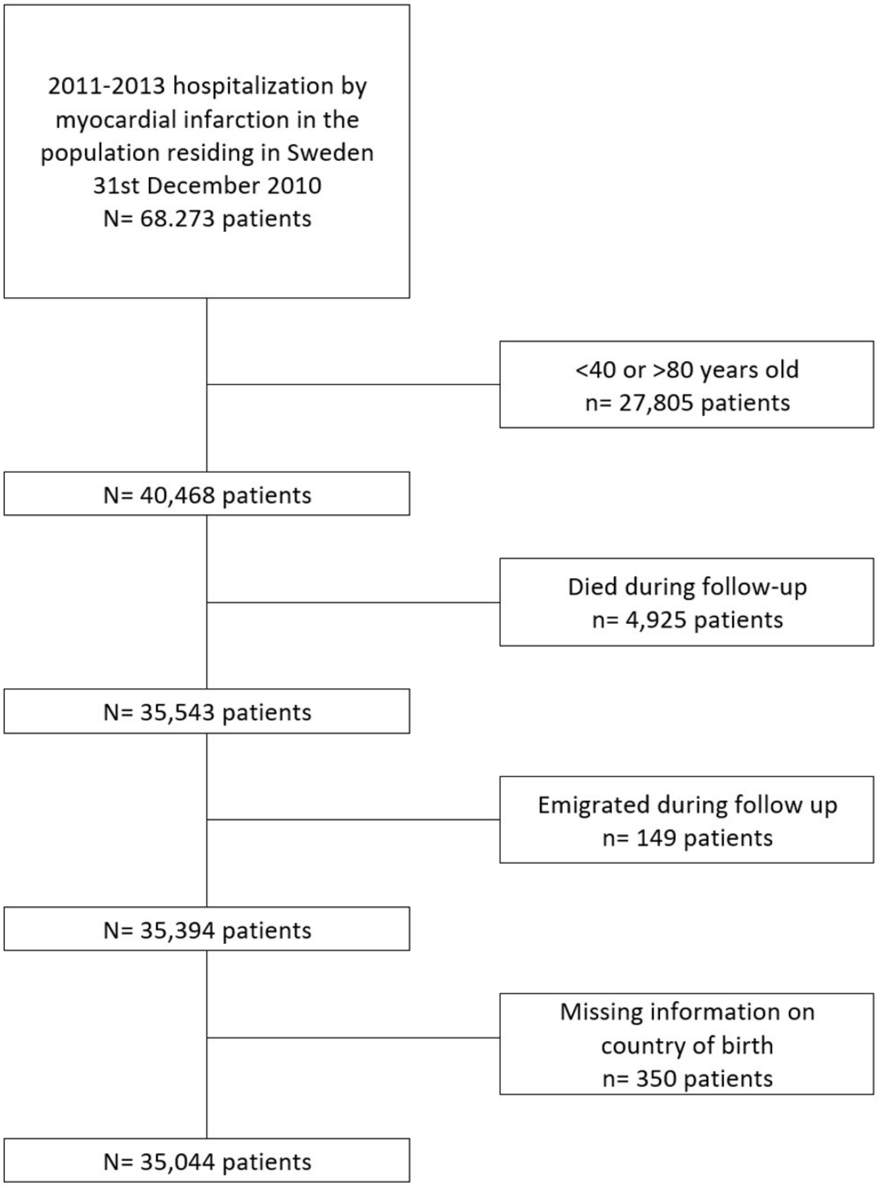

Figure 1: Study population flow-chart

261x348mm (96 x 96 DPI)

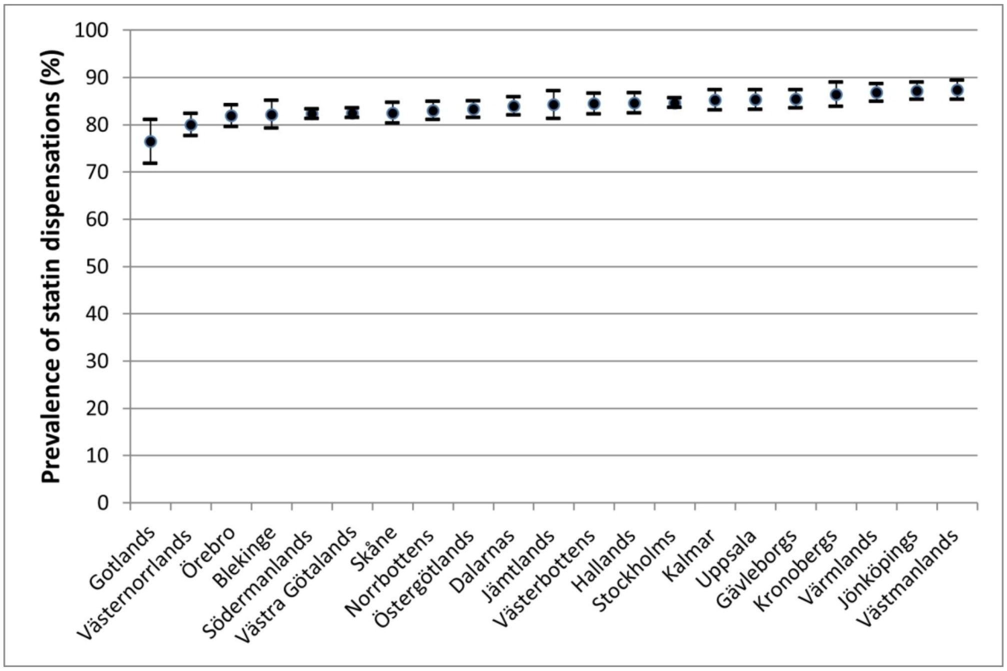

Figure 2: Crude prevalence (P) and 95% confidence intervals (CI) for use of statins 12 -18 months after hospital discharge with an acute myocardial infarction (AMI) diagnosis in patients aged 40-80 years in the period 2011-2013, across the 21 administrative regions of Sweden.

152x101mm (300 x 300 DPI)

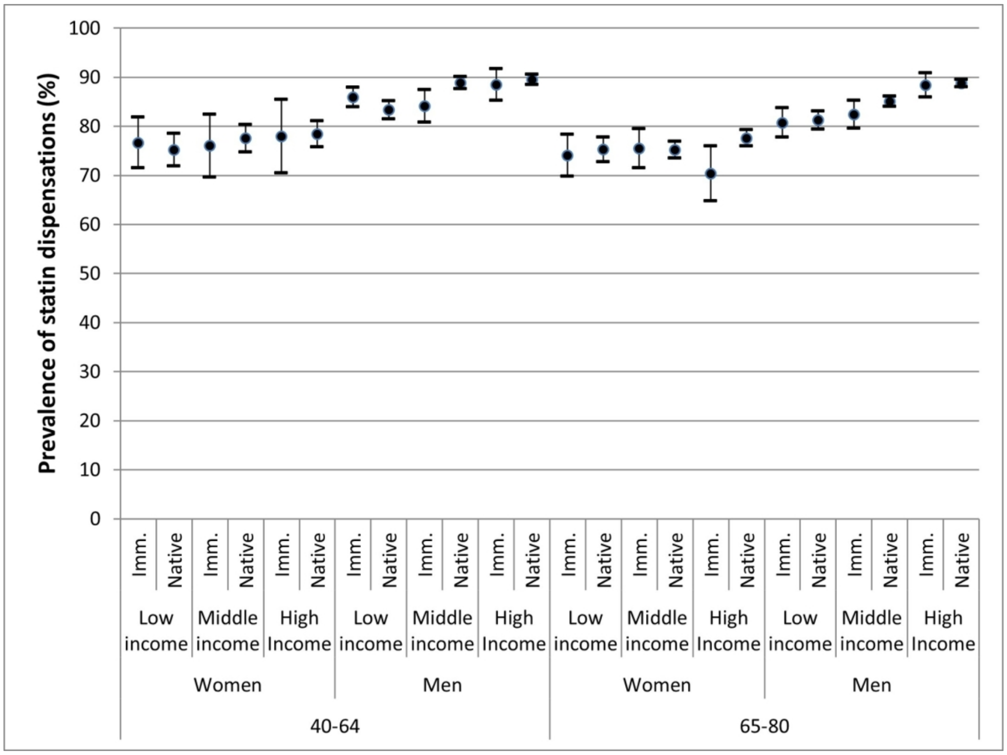

Figure 3: Crude prevalence (P) and 95% confidence intervals (CI) for use of statins 12 -18 months after hospital discharge with an acute myocardial infarction (AMI) diagnosis in patients aged 40-80 years in the period 2011-2013, across the 24 sociodemographic groups.

165x123mm (300 x 300 DPI)

STROBE Statement—Checklist of items that should be included in reports of ***cross-sectional studies***

	Item No	Recommendation	Page No
**Title and abstract**	1	(*a*) Indicate the study's design with a commonly used term in the title or the abstract	1-2
		(*b*) Provide in the abstract an informative and balanced summary of what was done and what was found	2
**Introduction**			
Background/rationale	2	Explain the scientific background and rationale for the investigation being reported	3-4
Objectives	3	State specific objectives, including any prespecified hypotheses	3-4
**Methods**			
Study design	4	Present key elements of study design early in the paper	4, 7-9
Setting	5	Describe the setting, locations, and relevant dates, including periods of recruitment, exposure, follow-up, and data collection	5-6
Participants	6	(*a*) Give the eligibility criteria, and the sources and methods of selection of participants	5
Variables	7	Clearly define all outcomes, exposures, predictors, potential confounders, and effect modifiers. Give diagnostic criteria, if applicable	6
Data sources/ measurement	8*	For each variable of interest, give sources of data and details of methods of assessment (measurement). Describe comparability of assessment methods if there is more than one group	5-6
Bias	9	Describe any efforts to address potential sources of bias	6
Study size	10	Explain how the study size was arrived at	5
Quantitative variables	11	Explain how quantitative variables were handled in the analyses. If applicable, describe which groupings were chosen and why	6-8
Statistical methods	12	(*a*) Describe all statistical methods, including those used to control for confounding	7-10
		(*b*) Describe any methods used to examine subgroups and interactions	7-10
		(*c*) Explain how missing data were addressed	5
		(*d*) If applicable, describe analytical methods taking account of sampling strategy	
		(*e*) Describe any sensitivity analyses	
**Results**			
Participants	13*	(a) Report numbers of individuals at each stage of study—eg numbers potentially eligible, examined for eligibility, confirmed eligible, included in the study, completing follow-up, and analysed	5, 20
		(b) Give reasons for non-participation at each stage	5, 20
		(c) Consider use of a flow diagram	20
Descriptive data	14*	(a) Give characteristics of study participants (eg demographic, clinical, social) and information on exposures and potential confounders	10-12, 23-24
		(b) Indicate number of participants with missing data for each variable of interest	20
Outcome data	15*	Report numbers of outcome events or summary measures	10-12, 23-24

For peer review only

Main results	16	(*a*) Give unadjusted estimates and, if applicable, confounder-adjusted estimates and their precision (eg, 95% confidence interval). Make clear which confounders were adjusted for and why they were included	10-12, 23-24
		(*b*) Report category boundaries when continuous variables were categorized	5, 9
		(*c*) If relevant, consider translating estimates of relative risk into absolute risk for a meaningful time period	
Other analyses	17	Report other analyses done—eg analyses of subgroups and interactions, and sensitivity analyses	
**Discussion**			
Key results	18	Summarise key results with reference to study objectives	12-13
Limitations	19	Discuss limitations of the study, taking into account sources of potential bias or imprecision. Discuss both direction and magnitude of any potential bias	14-16
Interpretation	20	Give a cautious overall interpretation of results considering objectives, limitations, multiplicity of analyses, results from similar studies, and other relevant evidence	13-16
Generalisability	21	Discuss the generalisability (external validity) of the study results	15-16
**Other information**			
Funding	22	Give the source of funding and the role of the funders for the present study and, if applicable, for the original study on which the present article is based	16-17

*Give information separately for exposed and unexposed groups.

Note: An Explanation and Elaboration article discusses each checklist item and gives methodological background and published examples of transparent reporting. The STROBE checklist is best used in conjunction with this article (freely available on the Web sites of PLoS Medicine at http://www.plosmedicine.org/, Annals of Internal Medicine at http://www.annals.org/, and Epidemiology at http://www.epidem.com/). Information on the STROBE Initiative is available at www.strobe-statement.org.

For peer review only

For peer review only - http://bmjopen.bmj.com/site/about/guidelines.xhtml

BMJ Open

Geographic and sociodemographic differences in statin dispensation after acute myocardial infarction in Sweden – A register based prospective cohort study applying analysis of individual heterogeneity and discriminatory accuracy (AIHDA) for basic comparisons of healthcare performance

Journal:	*BMJ Open*
Manuscript ID	bmjopen-2022-063117.R1
Article Type:	Original research
Date Submitted by the Author:	18-Nov-2022

Complete List of Authors:	Merlo, Juan; Lunds University Faculty of Medicine, Social Epidemiology<br>Öberg, Johan; Lund University Clinical Sciences, Unit for Social Epidemiology<br>Khalaf, Kani; Lund University, Department of Clinical Sciences<br>Perez-Vicente, Raquel; Lund University, Faculty of Medicine, Social Epidemiology<br>Leckie, George; University of Bristol, Multilevel Modelling

**Primary Subject Heading**:	Epidemiology
Secondary Subject Heading:	Epidemiology, Health services research
Keywords:	EPIDEMIOLOGY, Myocardial infarction < CARDIOLOGY, Quality in health care < HEALTH SERVICES ADMINISTRATION & MANAGEMENT, CLINICAL PHARMACOLOGY

SCHOLARONE™
Manuscripts

I, the Submitting Author has the right to grant and does grant on behalf of all authors of the Work (as defined in the below author licence), an exclusive licence and/or a non-exclusive licence for contributions from authors who are: i) UK Crown employees; ii) where BMJ has agreed a CC-BY licence shall apply, and/or iii) in accordance with the terms applicable for US Federal Government officers or employees acting as part of their official duties; on a worldwide, perpetual, irrevocable, royalty-free basis to BMJ Publishing Group Ltd ("BMJ") its licensees and where the relevant Journal is co-owned by BMJ to the co-owners of the Journal, to publish the Work in this journal and any other BMJ products and to exploit all rights, as set out in our licence.

The Submitting Author accepts and understands that any supply made under these terms is made by BMJ to the Submitting Author unless you are acting as an employee on behalf of your employer or a postgraduate student of an affiliated institution which is paying any applicable article publishing charge ("APC") for Open Access articles. Where the Submitting Author wishes to make the Work available on an Open Access basis (and intends to pay the relevant APC), the terms of reuse of such Open Access shall be governed by a Creative Commons licence – details of these licences and which Creative Commons licence will apply to this Work are set out in our licence referred to above.

Other than as permitted in any relevant BMJ Author's Self Archiving Policies, I confirm this Work has not been accepted for publication elsewhere, is not being considered for publication elsewhere and does not duplicate material already published. I confirm all authors consent to publication of this Work and authorise the granting of this licence.

For peer review only

Geographic and sociodemographic differences in statin dispensation after acute myocardial infarction in Sweden – A register based prospective cohort study applying analysis of individual heterogeneity and discriminatory accuracy (AIHDA) for basic comparisons of healthcare performance

Juan Merlo[1, 2] ORCID: 0000-0001-8379-9708

Johan Öberg[1, 3] ORCID: 0000-0002-0701-5155

Kani Khalaf[1, 3] ORCID: 0000-0002-6749-7037

Raquel Perez Vicente[1] ORCID: 0000-0002-6273-1656

George Leckie[4] ORCID: 0000-0003-1486-745X

[1] Unit for Social Epidemiology, Faculty of Medicine, Lund University, Malmö, Sweden

[2] Centre for Primary Health Care Research, Region Skåne, Malmö, Sweden

[3] Department of Health and Medical Care Management, Region Skåne, Malmö, Sweden

[4] Centre for Multilevel Modelling, University of Bristol, UK

Corresponding author: Prof. Juan Merlo juan.merlo@med.lu.se, Skåne University Hospital, CRC, Jan Waldenströms gata 35 building 60, floor 12, SE-205 02 Malmö, Sweden, +46 70 5092265

Keywords (MeSH): Social Epidemiology, Health Care Quality Assessment, Pharmacologies, Health Services Evaluation, Epidemiological Methods

Word count: 5710

For peer review only - http://bmjopen.bmj.com/site/about/guidelines.xhtml

Abstract word count: 247

Number of references: 41

For peer review only - http://bmjopen.bmj.com/site/about/guidelines.xhtml

Abstract

Objectives: In Sweden, as in many other countries, official monitoring of healthcare performance is mostly focused on geographical disparities in relation to a desirable benchmark. However, current evaluations could be improved by considering: (i) the intersection of other relevant axes of inequity like age, sex, income, and migration status; and (ii) the existence of individual heterogeneity around averages. Therefore, using an established quality indicator (i.e., dispensation of statins after acute myocardial infarction, AMI), we valuate both geographical and sociodemographic inequalities and illustrate how the analysis of individual heterogeneity and discriminatory accuracy (AIHDA) enhances such evaluations.

Population and methods: We applied AIHDA and calculated the area under the ROC curve (AUC) of regional and sociodemographic differences in the statin dispensations of 35,044 patients from 21 Swedish regions and 24 sociodemographic strata who were discharged from the hospital with an AMI diagnosis between January 2011 and December 2013. Following the Swedish National Board of Health and Welfare, we used a benchmark value of 90%.

Results: Dispensation of stains after AMI in Sweden did not reach the desired target of 90%. Regional differences were absent/very small (AUC = 0.537) while sociodemographic differences were small (AUC = 0.618). Women, especially those with

For peer review only - http://bmjopen.bmj.com/site/about/guidelines.xhtml

immigrant background and older than 65 years, have the lowest proportions of statin dispensations after AMI.

Conclusions: As the AUC statistics are small, interventions trying to achieve the benchmark value should be universal. However, a special emphasis should nevertheless be directed towards women, specially older women with immigrant backgrounds.

Strengths and limitations of this study:

- The applied Analysis of Individual Heterogeneity and Discriminatory Accuracy (AIHDA) provides an improved mapping and quantification of geographical and sociodemographic inequalities in healthcare quality
- The quality of the data in our study is high as it is based on national registers with valid and standardized information on diagnoses, medication use and sociodemographic variables
- We can directly compare our results to those presented by public authorities, such as the Swedish National Boars of Health and Welfare, since the definition of the quality indicator is the same.
- The definition and categorization of the sociodemographic variables is coarse. However, more fine categorizations would excessively increase the number of strata

For peer review only - http://bmjopen.bmj.com/site/about/guidelines.xhtml

- The AIHDA approach can be implemented using both traditional regression or more advanced multilevel models which provide several advantages. However, traditional regression is a valid and accessible alternative

For peer review only

For peer review only - http://bmjopen.bmj.com/site/about/guidelines.xhtml

Introduction

In Sweden as in many other countries, policies and laws emanating from patient needs regulate and define standards of healthcare[1]. Good healthcare is defined as safe, accessible, delivered in time, health promoting, and knowledge- and evidence-based. In addition, to be fully efficient and effective, good healthcare must be equitable. That is, on equal terms and according to patients' needs. On this background, The Swedish National Board of Health and Welfare (NBHW) and the Swedish Association of Local Authorities and Regions (SALAR) define quality indicators[2] that can be compared against specific benchmarks. These quality indicators are regularly monitored for performance assessment by means of simplified hospital and regional comparisons online (https://vardenisiffror.se/), and in reports such as *Open comparisons of healthcare performance*[3]. The benefits of monitoring for improving healthcare quality are many[4]. However, current comparisons could be improved for at least three reasons.

First, in spite of the current evidence on the existence of healthcare inequities conditioned by sociodemographic factors (e.g., age, sex, income, country of birth), today's official monitoring is mostly focused on geographical disparities, disregarding other relevant axes of possible inequity. Monitoring both geographic and sociodemographic inequalities seem necessary in order to improve the evaluation of healthcare performance[5] .

Second, comparisons are often based on the analysis and interpretation of differences

For peer review only - http://bmjopen.bmj.com/site/about/guidelines.xhtml

between group averages, disregarding individual heterogeneity around such averages[6 7], This is a fundamental but often ignored question in epidemiology and healthcare evaluation [6-9]

Third, the criteria for quantifying the size of the group differences in these comparisons are not clearly stated. For instance, when can we say that there are large regional differences in a quality indicator? The criterion of “statistical significance” is not sufficient as very small and meaningless differences between group averages may nonetheless prove statistically significant if the sample is large enough. Also, measurable differences between group averages may exist together with considerable overlap of individual heterogeneity between groups, which may render irrelevant the average differences (See elsewhere for an extended explanation of this concept[8 10]).

The analysis of individual heterogeneity and discriminatory accuracy (AIHDA) is an innovative analytical framework that has the potential to address the above criticisms and thereby improve the quantitative evaluation of health care quality. AIHDA is proving an increasingly popular approach in public health and health care epidemiology [5 8 9 11-20]. When applying AIHDA, we do not consider differences between group averages and differences between patients as if they are two separate and unrelated phenomena of interest. Rather, we adopt a conceptual multilevel perspective that simultaneously considers both types of differences. From this standpoint, we evaluate sociodemographic and geographical group differences by quantifying the share of the total individual

For peer review only - http://bmjopen.bmj.com/site/about/guidelines.xhtml

variation in patient outcomes that operates at the group level. In the case of binary patient outcomes (like most quality indicators), we can analogously evaluate the accuracy of the group level for discriminating individuals with the outcome from those without the outcome of interest. Applying AIHDA, we can evaluate the relevance of specific group differences (e.g., regional differences), and even compare the relative relevance of different grouping criteria (e.g., sociodemographic strata versus geographical regions). AIHDA can be applied using traditional regression[16] or more advanced multilevel models[5 8 9 13]. While we primarily recommended the use of multilevel models[21], for simplified routine monitoring and online presentations, traditional regression analysis provides a valid alternative.

Motivated by the above background, we aim to illustrate how the AIHDA approach can be applied in official online comparisons and reports. For doing so we analyze statin dispensation after acute myocardial infarction (AMI), which is a well-known process indicator specifically adopted by the Swedish NBHW[22]. In addition to geographical differences[4], research has shown sociodemographic inequalities in statin treatment among AMI patients, with less dispensations in women[23], immigrants[24], elderly people[25] and in patients with low socioeconomic position[26].

In summary, the aim of this study is to illustrate the use of AIHDA for public monitoring of sociodemographic and geographical differences in statin treatment after AMI hospitalization. For this purpose, we analyze the statins dispensations of 31,027 patients

For peer review only - http://bmjopen.bmj.com/site/about/guidelines.xhtml

discharged from the hospital with an AMI diagnosis between January 2011 and December 2013.

Population and methods

Databases

Our investigation is based on a record-linkage database of several registers with national coverage, including the Swedish Population Register (TPR) and the Longitudinal Integration Database for Health Insurance and Labour Market Studies (LISA), administrated by Statistics Sweden (SCB), as well as the National Patient Register (NPR), the Cause of Death Register, and the Swedish Prescribed Drug Register (SPDR) administrated by the NBHW. The NPR records both outpatient clinic and inpatient discharges from hospital with diagnoses coded according to the International Classification of Diseases, 10th edition (ICD-10). However, it does not cover information on diagnoses in primary health care. The SPDR contains information on all prescription drug dispensations from Swedish pharmacies. To maintain confidentiality, the registers were linked using an arbitrary serial number assigned to each individual by statistics Sweden instead of the Swedish unique personal identification number .

The Regional Ethics Review Board in Southern Sweden and the data safety committees from the NBHW and from Statistics Sweden approved the construction of the database.

For peer review only

For peer review only - http://bmjopen.bmj.com/site/about/guidelines.xhtml

Study population

We defined the study population using the same criteria as applied by the Swedish NBHW for evaluating the quality indicator on statins dispensation after hospital discharge for AMI.

The initial population included all 40,468 patients aged 40-80 years, who were residing in Sweden by 31 December 2010, and were discharged from hospital with an *AMI diagnosis* (ICD-10: I21 or I22) between 1 January 2011 and 31 December 2013. We followed each patient between the 13th and 18th month after discharge from the index AMI hospitalization regarding their statin dispensation from the pharmacies. We excluded 4,925 patients who died between discharge from hospital and end of follow-up,149 patients who emigrated, and 350 patients with missing information on country of birth. The final study population included 35,044 patients. See figure 1 for details.

Assessment of variables

We defined the *quality indicator* according to the Swedish authorities as at least 90% of patients aged 40-80 years with a hospital discharge diagnosis of AMI and as having had at least one statin dispensation between the 13th and 18th month period after the hospital discharge[22]. We define *statin use* as any recorded dispensation of statin or a statin-combination drug (ATC codes C10AA or C10BA) in the SPDR during the follow-up period.

For peer review only - http://bmjopen.bmj.com/site/about/guidelines.xhtml

We classified the patients into the 21 *administrative regions* of Sweden according to their legal residence address and used Region Skåne as the reference region in the comparisons. Administrative regions in Sweden form autonomous political units and act as principals for healthcare.

We categorized *age* into two groups: 40-64 years and 65-80. *Sex* was defined as male or female according to the registered information. We categorized patients according to their *country of birth* into native (i.e., born in Sweden) or not (i.e., immigrant).

We obtained information on *individualised disposable family income* for the years 2000, 2005 and 2010 to compute a cumulative income measure that considers the size of the household and the consumption weight of the individuals according to Statistics Sweden. For each of the three years, income levels were categorised into 25 quantiles using the complete Swedish population. These groups from the respective three years were summed up, so that each individual received a value between 3 (always in the lowest income group) and 75 (always in the highest income group). We categorised this cumulative income measure into three groups by tertiles (low, medium, or high income). Individuals with missing values on income during 2000 or 2005 were assigned the tertile values for the year 2010. No individuals had missing income data for 2010. By calculating a cumulative income measure, we obtain a more stable measurement of socioeconomic position.

For peer review only - http://bmjopen.bmj.com/site/about/guidelines.xhtml

For peer review only

Finally, we constructed a *sociodemographic multicategorical variable* consisting of all possible combinations of the categories of the four sociodemographic variables age, sex, income, and immigration status. This resulted in 24 strata.

Patient and public involvement

Because of the nature of our study, patient and public involvement is necessary. We will communicate the results of our study to politicians and healthcare professionals, who represent the public's interest.

Statistical and epidemiological analysis

To examine the association between geographical and sociodemographic variables and the use of statins, we calculated the crude percentage of patients who were dispensed statins in each region and in each multicategorical sociodemographic strata.

Next, we constructed three regression models following the AIHDA approach. Since the prevalence of the outcome was not extreme, we measured the associations between use of statins and the explanatory variables by prevalence ratios (PRs) and their 95% confidence intervals (CI) rather than by odds ratios. For this purpose, we applied Cox proportional hazards regression models with a constant follow-up time equal to 1 and report heteroskedasticity robust confidence intervals [27] using the vce(robust) option in Stata (see S2).

For peer review only - http://bmjopen.bmj.com/site/about/guidelines.xhtml

Model 1A estimates geographical inequalities by including only the geographical variable with the 21 regions, specifying Region Skåne as reference in the comparisons.

Model 1B estimates sociodemographic inequalities by including the sociodemographic multicategorical variable only, specifying the strata of native 40-64 years old men with high income as the reference category. The focus of our study was the sociodemographic multicategorical variable, but for informative reasons we performed a series of intermediate models one variable in turn from the set of variables used to construct the multicategorical variable (see supplementary information S1).

Model 2 enters the geographical variable and the sociodemographic multicategorical variable simultaneously. This model estimates the geographical and sociodemographic differences adjusted for each other

In order to evaluate and compare the relevance of the sociodemographic and geographical information for understanding use of statins after AMI, we estimated the Discriminatory accuracy (DA) for each model by calculating the area under the receiver operating characteristic curve (AUC) with 95% confidence intervals (CIs). The AUC is obtained by plotting the true-positive fraction against the false-positive fraction for different thresholds of the predicted probability of statin use. The value of the AUC ranges from .5 to 1, with .5 indicating the model has no predictive accuracy and 1 representing perfect discrimination. Using the criteria proposed by Hosmer and

For peer review only - http://bmjopen.bmj.com/site/about/guidelines.xhtml

Lemeshow [28], we classified DA as absent or very small (.5 ≤ AUC ≤ .6), small (.6 < AUC ≤ .7), large (.7 < AUC ≤ 0.8), or very large (AUC > .8). As explained in previous publications [8 10], measuring geographical and sociodemographic differences to evaluate healthcare quality is analogous to performing a diagnostic test [29]. No decision-maker in healthcare would want to use such a test without first knowing its discriminatory accuracy (i.e., sensitivity, specificity, AUC). We do not want to unnecessarily treat false positive individuals or incorrectly deny treatment of false negative patients. In our case, we want to know the accuracy of public sociodemographic and geographical comparisons for discriminating individuals with a positive result of the quality indicator (i.e., use of statins) from those with a negative result (i.e., no statins).

Strategy for evaluating sociodemographic and geographical differences

Applying the AIHDA framework means that we focus on both the group averages (that is, the region and sociodemographic strata averages) and the individual heterogeneity around these averages (that is, the AUC value, which informs on the overlap in individual heterogeneity across groups).

This framework includes four steps to achieve a complete analysis of inequalities. However, the approach may be modified for different research questions[8].

In the *first step (i)* we identify the quality indicator and the target or benchmark average value as we described above. In the *second step (ii)* we estimate and

visualize the groups' differences using appropriate plots (e.g., caterpillar plots) and in the *third step (iii)* we quantify the size of these differences. That is, for a correct interpretation of the plots, the size of the groups' differences needs to be evaluated using a measure of discriminatory accuracy like the AUC. In traditional comparisons of group averages, the criteria for quantifying the size of the geographical and sociodemographic differences are not clearly stated. However, in the AIHDA framework we evaluate geographical and sociodemographic differences by quantifying the share of the total variation in individual outcomes that operates at the group level (i.e., regions, sociodemographic strata). In the case of binary individual health outcomes, we can use the AUC as it provides information expressed in terms of discriminatory accuracy. Finally, in the *fourth step (iv)* we proceed to the interpretation of the results. Following the study referred earlier[8], we present a practical framework for evaluating geographical and sociodemographic differences. For this purpose, we need at least two types of information.

First, we need to know whether the average quality indicator (i.e., percentage of patients on statins after AMI) has reached the predetermined target value fully (i.e., ≥ 90% of all AMI patients are on statins), closely (i.e., 80-89%), or insufficiently (i.e., < 80%). The criteria we use to qualify the prevalence value as "fully" is based on the definition given by Swedish NBHW. However, the qualifications "closely" and "insufficiently" are ours and therefore subjective. More generally, the thresholds defining these categories will differ

For peer review only - http://bmjopen.bmj.com/site/about/guidelines.xhtml

across quality indicators.

Second, we need information on the *size of the group differences* as assessed by the AUC, which expresses the accuracy of the differences between group averages for discriminating patients with the outcome from those without the outcome. These two types of information need to be combined for the evaluation. Box 1 illustrates these ideas by means of a simple framework with 12 scenarios that can be used to orient the interpretation of an analysis.

For peer review only

For peer review only - http://bmjopen.bmj.com/site/about/guidelines.xhtml

Box 1. Framework for evaluating geographical and sociodemographic differences in a specific quality indicator. For this purpose, we need information about the *target indicator value*. That is, whether the average quality indicator (i.e., percentage of patients on statins after AMI) has "insufficiently", "closely" or "fully" reached a predetermined target *benchmark* level. We also need information on the area under receiver operating characteristics curve (AUC), which expresses the accuracy of the differences between group averages for discriminating patients with the outcome from those without the outcome. Combining this information, we obtain 12 different scenarios useful for the evaluation.

		**Target indicator value reached**		
**Size of the group differences**		Insufficiently	Closely	Fully
Absent/very small	.5 ≤ AUC ≤ .6	A	B	C
Small	.6 ≤ AUC < .7	D	E	F
Large	.7 ≤ AUC < .8	G	H	I
Very large	.8 ≤ AUC	J	K	L

In the ideal scenario (scenario C) the desired target level has been fully reached in the population, and the group differences are absent or very small. The conclusion would be that all groups have been treated appropriately and on equal terms. From this perspective, the quality of the healthcare is high.

In the worst scenario (scenario A) the desired target level has not been achieved in the population, and the groups differences are again absent. The conclusion would be that all groups have been treated on equal terms but inadequately. That is, the quality of healthcare in the whole population is low.

For peer review only - http://bmjopen.bmj.com/site/about/guidelines.xhtml

Observe that in both scenarios A and C interventions targeted to specific groups are not justifiable. Rather any intervention should be universal (i.e., directed to the whole country). In scenario C, the reason for the intervention would be to maintain the desirable value of the quality indicator in all groups. In the scenario A, the reason would be to reach this desirable level in all the groups.

The interpretation of the scenarios in the lowest corners of the table (J and L) is conditioned by the very large size of the group differences. For example, in scenario L even though the population as a whole has achieved the target level, some groups may not have done so. In contrast, in scenario J, some groups may have achieved the target level even though the population as a whole has not achieved it. In these scenarios (J and L) targeted group interventions are justified.

The framework we propose fits well with the concept of *proportionate universalism* for resource allocation in public health [30 31]. That is, health care actions must be universal, not targeted, but with a scale and intensity that is proportionate to the level of disadvantage. The AIHDA approach we apply in this study can be used to inform decisions regarding the appropriate scale and intensity for a given sociodemographic or geographic context.

For peer review only - http://bmjopen.bmj.com/site/about/guidelines.xhtml

Results

The overall prevalence of statin dispensations in AMI patients 12-18 months after discharge was 83.7% (29,231/35,044). Therefore, the benchmark value of 90% was *closely* but not fully met on a national level according to the framework in Box 1.

Figure 2 and Table 1 illustrate the prevalence of statin dispensations in each administrative region in Sweden. Västmanland has the highest prevalence of statin use at 87.4% while Gotland has the lowest prevalence at 76.4%. None of the regions reach the benchmark value of 90%. The relative differences when comparing with the region of Skåne (Model 1A) were tiny and Table 1 illustrates that only four regions (i.e., Gotland, Västernorrland, Stockholm and Västra Götaland) presented a slightly but conclusively lower PR than Skåne. No other region showed a conclusively higher PR than Skåne. The PR adjusted for the multicategorical sociodemographic variable (Model 2) were very similar to the unadjusted PR (Model 1A), indicating that the sociodemographic composition of the regions did not confound the geographical differences.

For peer review only

Table 1. Number of patients using statins (n), number of individuals (N), crude prevalence (P) and 95% confidence intervals (CI), unadjusted prevalence ratios (PR), and prevalence ratios adjusted for sociodemographic variables (PRa) for use of statins 12 -18 months after hospital discharge with an acute myocardial infarction (AMI) diagnosis in patients aged 40-80 years from the 21 Swedish geographical administrative regions in the period 2011-2013.

				Model 1A	Model 2
	n	N	P (95% CI) %	PR (95% CI)	PRa (95% CI)

For peer review only - http://bmjopen.bmj.com/site/about/guidelines.xhtml

Sweden	29321	35044	83.67 (83.28-84.06)	–	–
Administrative Regions					
Gotland	246	322	76.40 (71.76-81.04)	0.90 (0.85-0.96)	0.91 (0.85-0.97)
Västernorrland	922	1,153	79.97 (77.65-82.28)	0.95 (0.92-0.98)	0.94 (0.92-0.97)
Örebro	886	1,082	81.89 (79.59-84.18)	0.97 (0.94-1.00)	0.97 (0.94-1.00)
Blekinge	525	639	82.16 (79.19-85.13)	0.97 (0.93-1.01)	0.98 (0.94-1.02)
Stockholm	4,477	5,440	82.30 (81.28-83.31)	0.97 (0.96-0.99)	0.97 (0.95-0.98)
Västra Götaland	4,391	5,326	82.44 (81.42-83.47)	0.97 (0.96-0.99)	0.97 (0.96-0.99)
Uppsala	960	1,164	82.47 (80.29-84.66)	0.97 (0.95-1.00)	0.97 (0.94-1.00)
Norrbotten	1,215	1,465	82.94 (81.01-84.86)	0.98 (0.95-1.01)	0.98 (0.95-1.00)
Östergötland	1,400	1,682	83.23 (81.45-85.02)	0.98 (0.96-1.01)	0.98 (0.96-1.01)
Dalarna	1212	1,444	83.93 (82.04-85.83)	0.99 (0.97-1.02)	0.99 (0.97-1.02)
Jämtland	501	595	84.20 (81.27-87.13)	1.00 (0.96-1.03)	1.00 (0.96-1.03)
Västerbotten	893	1,058	84.40 (82.22-86.59)	1.00 (0.97-1.03)	0.99 (0.97-1.02)
Halland	940	1,112	84.53 (82.41-86.66)	1.00 (0.97-1.03)	1.00 (0.97-1.02)
Skåne	4,085	4,828	84.61 (83.59-85.63)	Reference	Reference
Kalmar	898	1,054	85.20 (83.06-87.34)	1.01 (0.98-1.04)	1.01 (0.98-1.04)
Södermanland	973	1,141	85.28 (83.22-87.33)	1.01 (0.98-1.04)	1.01 (0.98-1.04)
Gävleborg	1,126	1,318	85.43 (83.53-87.34)	1.01 (0.98-1.04)	1.01 (0.99-1.04)
Kronoberg	592	685	86.42 (83.86-88.99)	1.02 (0.99-1.05)	1.02 (0.99-1.05)
Värmland	1,076	1,240	86.77 (84.89-88.66)	1.03 (1.00-1.05)	1.03 (1.00-1.05)
Jönköping	1,126	1,292	87.15 (85.33-88.98)	1.03 (1.01-1.06)	1.03 (1.00-1.05)
Västmanland	877	1,004	87.35 (85.29-89.41)	1.03 (1.01-1.06)	1.04 (1.01-1.06)
Area under the ROC curve (AUC)				0.537	0.618

For peer review only

Figure 3 and Table 2 illustrate the crude prevalence of statin dispensations for each sociodemographic stratum, with the highest prevalence (i.e., 89.5%) in the reference strata of 40–64-year-old native men with high income, followed by the strata of 40–64-year-old native men with middle income (i.e., 88.8%). We found the lowest prevalence of statin use in the stratum of 65–80-year-old immigrant women with high income (i.e., 70.3%), closely followed by 65–80-year-old immigrant women with low income (i.e., 74.1%). Only the reference sociodemographic stratum almost grasped the target value of

For peer review only - http://bmjopen.bmj.com/site/about/guidelines.xhtml

90% (i.e., 89.5%). None of the female strata were close to reaching the target. In

Table 2. Number of patients using statins (n), number of individuals (N), crude prevalence (P) and 95% confidence intervals (CI), unadjusted prevalence ratios (PR), and prevalence ratios adjusted for administrative regions (PRa) for use of statins 12-18 months after hospital discharge with an acute myocardial infarction (AMI) diagnosis in patients aged, 40-80 years old from 24 sociodemographic strata in the period 2011-2013

				Model 1B	Model 2
	n	N	P (95% CI) %	PR (95% CI)	PRa (95% CI)
Sweden	29,321	35,044	83.67 (83.28-84.06)	–	–
Women					
65-80 , High income, Immigrant	180	256	70.31 (64.72-75.91)	0.79 (0.72-0.85)	0.79 (0.73-0.85)
65-80 , Low income, Immigrant	300	405	74.07 (69.81-78.34)	0.83 (0.78-0.88)	0.83 (0.78-0.88)
65-80 , Middle income, Native	1,936	2,575	75.18 (73.52-76.85)	0.84 (0.82-0.86)	0.84 (0.82-0.86)
40-64 , Low income, Native	479	637	75.20 (71.84-78.55)	0.84 (0.80-0.88)	0.84 (0.80-0.88)
65-80 , Low income, Native	852	1,132	75.27 (72.75-77.78)	0.84 (0.81-0.87)	0.84 (0.81-0.87)
65-80 , Middle income, Immigrant	338	448	75.45 (71.46-79.43)	0.84 (0.80-0.89)	0.84 (0.80-0.89)
40-64 , Middle income, Immigrant	130	171	76.02 (69.62-82.42)	0.85 (0.78-0.92)	0.85 (0.78-0.93)
40-64 , Low income, Immigrant	197	257	76.65 (71.48-81.83)	0.86 (0.80-0.92)	0.86 (0.80-0.92)
40-64 , Middle income, Native	656	846	77.54 (74.73-80.35)	0.87 (0.83-0.90)	0.86 (0.83-0.90)
65-80 , High income, Native	1,838	2,369	77.59 (75.91-79.26)	0.87 (0.85-0.89)	0.87 (0.85-0.89)
40-64 , High income, Immigrant	92	118	77.97 (70.49-85.44)	0.87 (0.79-0.96)	0.87 (0.79-0.96)
40-64 , High income, Native	709	904	78.43 (75.75-81.11)	0.88 (0.85-0.91)	0.87 (0.84-0.91)
Men					
65-80 , Low income, Immigrant	548	679	80.71 (77.74-83.67)	0.90 (0.87-0.94)	0.90 (0.87-0.94)
65-80 , Low income, Native	1,409	1,735	81.21 (79.37-83.05)	0.91 (0.88-0.93)	0.91 (0.88-0.93)

contrast, the male strata were often much closer to reaching the target. Table 2 also presents the unadjusted PR (Model 1B), and the PR adjusted for the geographical variable (Model 2), with the strata of 40–64-year-old high-income native men as reference stratum. As many as 19 sociodemographic strata show conclusively smaller PRs than the reference. The adjusted PR were very similar to the unadjusted PR indicating that geographical factors did not confound the sociodemographic differences.

For peer review only - http://bmjopen.bmj.com/site/about/guidelines.xhtml

65-80 , Middle income, Immigrant	565	686	82.36 (79.51-85.21)	0.92 (0.89-0.95)	0.92 (0.89-0.95)
40-64 , Low income, Native	1,283	1,540	83.31 (81.45-85.17)	0.93 (0.91-0.95)	0.93 (0.91-0.95)
40-64 , Middle income, Immigrant	397	472	84.11 (80.81-87.41)	0.94 (0.90-0.98)	0.94 (0.90-0.98)
65-80 , Middle income, Native	3,791	4,458	85.04 (85.04-85.04)	0.95 (0.93-0.97)	0.95 (0.93-0.96)
40-64 , Low income, Immigrant	1,006	1,171	85.91 (83.92-87.90)	0.96 (0.94-0.98)	0.96 (0.94-0.99)
65-80 , High income, Immigrant	570	645	88.37 (88.37-88.37)	0.99 (0.96-1.02)	0.99 (0.96-1.02)
40-64 , High income, Immigrant	337	381	88.45 (85.24-91.66)	0.99 (0.95-1.03)	0.99 (0.95-1.03)
65-80 , High income, Native	6,303	7,101	88.76 (88.76-88.76)	0.99 (0.98-1.01)	0.99 (0.98-1.00)
40-64 , Middle income, Native	2,240	2,522	88.82 (87.59-90.05)	0.99 (0.97-1.01)	0.99 (0.97-1.01)
40-64 , High income, Native	3,165	3,536	89.51 (88.50-90.52)	Reference	Reference
Area under the ROC curve (AUC)				0.609	0.618

Model 1A, which includes information about administrative regions, presented an AUC value of 0.537. This AUC value indicates that the geographical differences were *absent/very small,* and that region provides very limited information about statin dispensations. Model 1B, which includes information on the sociodemographic strata, showed an AUC of 0.609 which indicates that the sociodemographic differences were *small*. The sociodemographic AUC was 0.072 units higher than the AUC of the geographical model 1A. Model 2, which includes both geographical and sociodemographic information, presented an AUC value of 0.618, just 0.008 higher than that presented by model 1B. In the context of our analysis, these findings indicates that sociodemographic information is relatively more relevant than geographic information. In summary, the target indicator value was *closely* reached in Sweden as a whole. The geographical analysis indicated *absent/very small* differences, which corresponds with scenario B in Box 1. The sociodemographic analysis indicated *small* differences, which corresponds with the scenario E in Box 1.

For peer review only - http://bmjopen.bmj.com/site/about/guidelines.xhtml

In relation to the sociodemographic differences, the complementary analysis (S1) showed that the AUC was very small when we fitted separate models which included only one variable at a time. The highest AUC was for the variable sex (AUC = 0.579) which was 0.029 units lower than the AUC for all variables together (AUC = 0.608). We also observed a very small interaction of effects in the model with the multicategorical variable as when comparing with model with all the single variables together, the AUC of the model with the multicategorical variable only increases 0.001 units.

Discussion

We evaluated regional and sociodemographic inequalities in statin treatment after AMI. When doing so, we also aimed to demonstrate how AIHDA provides enhanced information for evaluating healthcare inequalities [8 32]. While traditional public comparisons focus on differences between group averages, AIHDA considers both differences between group averages and the individual heterogeneity around those averages as expressed by their discriminatory accuracy. AIHDA informs on the share of the total individual variation in patient outcomes that operates at the group level. That is, to which degree the group-level information is able to discriminate with accuracy patients with, from those without the indicator under study[8]. The idea of discriminatory accuracy is fundamental in many fields of epidemiology not only when evaluating statistical model

For peer review only - http://bmjopen.bmj.com/site/about/guidelines.xhtml

prediction,[33] but also in the evaluation of diagnostic and prognostic tests[29], as well as risk factors in public health[6] and in the study of geographical[32] and socioeconomic[18] determinates of health. It is also a cornerstone in current precision medicine[34]. Nevertheless, these concepts need wider adoption still and we refer elsewhere for a longer explanation[6] [7 8] [9].

The overall prevalence of statin treatment in our study population was 83.7% which while lower was judged high enough to have “closely” reached the target benchmark value of 90%. We observed regional and sociodemographic differences in the prevalence of statin treatment after AMI. However, the low AUC values indicated that the differences were “absent/very small” (AUC = 0.537) for the administrative regions and “small” (AUC = 0.609) for the sociodemographic groups. Combining both regional and sociodemographic information did not substantially increase the AUC of the sociodemographic information alone (AUC = 0.618). That is, the sociodemographic differences seem to play a modest but more relevant role than the regional differences for understanding inequalities in statin treatment after AMI.

Our analysis also shows that women in all strata have a lower prevalence of statin dispensations after AMI than men, which is in line with previous research indicating the existence of gender inequalities [23]. However, those inequalities need be interpreted in the light of the small DA of the sociodemographic strata. In fact, while the prevalence of using statins is higher among men, the fact that AMI patients are disproportionately male means that majority of non-statin users are nevertheless also men.

For peer review only - http://bmjopen.bmj.com/site/about/guidelines.xhtml

According to the framework presented in box 1, the sociodemographic differences would be placed in scenario E. That is, the target value was closely but not fully reached and there were small differences between the sociodemographic strata. Accordingly, any strategy aimed to improve the outcome of the quality indicator should be universal but proportionately more intense among sociodemographic groups with increased risk not using statins. Analogously, the differences between administrative regions would be placed in the scenario B. In this case we should recommend universal interventions without targeting any specific regions.

Traditional studies exclusively based on differences between group averages implicate several disadvantages as compared to AIHDA. It is known that epidemiological studies comparing group averages may *stigmatize* the individuals belonging to groups with "bad" average values and create *false expectations* for the individuals belonging to groups with "good" average values. This situation is true for both traditional and AIHDA studies and it is an inevitable side effect in epidemiology. Nevertheless, the perils of stigmatization and false expectations are often ethically justifiable because the aim of the epidemiological analyses is to improve the health and heath care of the individuals. However, knowing the DA of the group comparisons allows us to decide whether pointing out specific population groups is justifiable or not. If the DA is high, pointing out specific population groups is justifiable. However, if the DA is low, the traditional emphasis on differences between group averages should be avoided. For instance, our results show that,

For peer review only - http://bmjopen.bmj.com/site/about/guidelines.xhtml

because the AUC value is low, differences between administrative regions are less relevant for understanding individual statin dispensations. Therefore, simplistic interpretation based on visual analyses of figures like the caterpillar plot in our study (Figure 2) should be sidestepped.

By overlooking the DA of the group-averages, traditional studies may simultaneously lead to both under and overtreatment if a possible intervention is based on group information only. If the DA is low, many individuals taking statins but belonging to a group with a “bad” average (i.e., false positives cases) could be exposed to the intervention while many individuals not taking statins but belonging to a group with a “good” average (i.e., false negative cases) would not receive any intervention. For instance, using the information in Table 1, we can see that the region of Gotland has the worst average value (i.e., 76.4%), meaning that 76 of the 322 patients were not dispensed statins. However, in the region of Västmanland with the best average value (i.e., 87.4 %), this figure was 127 of the 1004 patients since Västmanland has a larger population. That is, the “best” region has 1.7 (= 127/76) times more patients lacking statin treatment that the “worst” region. It is correct that the region of Västmanland has a higher proportion of treated patients but from a national perspective the burden of untreated patients is also higher in the region of Västmanland. Therefore, if the DA is low, any potential intervention to increase statin treatment after AMI needs to be universal (i.e., on the whole country) and directed to all AMI patients no matter the region they belong to.

For peer review only - http://bmjopen.bmj.com/site/about/guidelines.xhtml

Similarly, in Table 2 we can estimate that the number of male AMI patients without statins (n = 3,312) was much higher than the number of female AMI patients without statins (n = 2,411) even though women consistently have a lower prevalence and a lower PR of statin dispensation than men. This reflects that while the prevalence of using statins is higher among men, the fact that AMI patients are disproportionately male means that majority of non-statin users are nevertheless also men.

Strengths and limitations

One strength of this study is the quality of the data since all information is based on national registers where the information is recorded in a highly standardized way for both ICD and ATC codes. The validity of the AMI diagnosis as recorded in the NPR is high, [35] and the sociodemographic information is recorded by Statistics Sweden with a longstanding experience in the field [https://www.scb.se/en/About-us/main-activity/quality-work/]. Moreover, we can directly compare our results to those presented by the NBHW since the definition of the quality indicator is the same. Another methodological strength in this study is the incorporation of a measurement of DA, which allows assessing differences between geographical and sociodemographic categories.

One limitation of this study is that we excluded the 5,424 individuals who died, emigrated, or had missing information on country of birth which corresponds to approximately 13%

For peer review only - http://bmjopen.bmj.com/site/about/guidelines.xhtml

of the final study population, and it cannot be ruled out that this exclusion introduces a selection bias. However, we do not think that this limitation alters our conclusions as the selection criteria are in concordance with the those used by the Swedish authorities. Another limitation in this study could be the use of household income as a proxy for socioeconomic position, which does not capture other financial assets, nor necessarily higher education.

The definition of country of birth within this study could be elaborated with more refined categories, other than native and immigrant. Immigrant is a broad concept which includes, among other factors, cultural, economic, and educational heritages from another countries. However, more categories of country of birth would result in a larger number of multicategorical strata with a smaller number of within strata observations. Similarly, there is a possible underestimation of the impact of age since we only use two categories in our analyses. We chose two categories to keep the analysis as simple as possible and also because in the case of the quality indicator we analyze we think it is sufficient to include the age of retirement (i.e., 65 years for most patients at the time of the study) as the cut-off. It is know that this division is relevant for understanding socioeconomic differences in health care utilization [36]. However, for other quality indicators it may be better to provide a more age classes.

We use the AUC as a measure of discriminatory accuracy and in some situations when e.g., false negatives are more likely to occur than false positives because of information

For peer review only - http://bmjopen.bmj.com/site/about/guidelines.xhtml

bias, assuming the same weight for both false negatives and positive might give misleading AUC values. However, this is not a problem in biomedical applications in general and in our study in particular as positive or negative events are easy to identify (a patient uses statins or not) and the information is, less probably, differentially biased. The AUC value may also depend on the size of the group being compared. For instance, a stratum may have a high predicted probability of using statins so all individuals in the stratum are classified as "positive" but if the stratum is very large the number of individuals in the stratum who do not use statins (i.e., false positive) may be large and the AUC reduced. This could be prevented by weighting the strata by the inverse of the number of individuals or using other measures of discriminatory accuracy like those based on components of variance.[8] However, in our case the unweighted results are relevant *per se*. A low unweighted AUC would suggest that any potential intervention to increase use of statins should not be targeted to specific strata with a low predicted probability of statin use but be universal and directed to all the strata since there are many patients in the strata with high predicted probabilities who do not use statins. For instance, in our study 37% (2,146/5,723) of the patients who did not use statins reside in the 10 counties with highest predicted probability of using statins, and this value was 58% (3,312/5,723) for the 12 sociodemographic strata with highest predicted probability of using statins.

For peer review only

Conceptually, the AIHDA approach adopts a multilevel perspective that partitions the individual differences into within groups (level 1) and between groups (level 2). Therefore, it would be appropriate to denominate the approach as Multilevel AIHDA or "MAIHDA". From the statistical point of view, the AIHDA approach can be implemented using both traditional regression analyses with the group as fixed effects [12 14 16 37] or more advanced multilevel regression analyses (MLRA) with a random intercept effect for the groups [5 8 9 11 13 15 17-19]. MLRA provide conceptual and methodological advantages as compared to traditional fixed effects analyses[21]. For instance, both fixed and random effects analyses considers the groups as contexts but in the traditional fixed effects analyses all information is modelled at the first level (individual) while in MLRA the groups are explicitly modeled as a second level. In MLRA the relative differences (e.g., PR) have the grand mean (i.e., the mean of the groups means) as reference while in traditional analyses the reference is a specific group (as in our present study). MLRA can better handle groups with few individuals via providing reliability weighted estimations. In addition to the AUC for assessing the importance of the group effects [38], MLRA also provides the variance partition coefficient (VPC) [32 39]. The VPC quantifies the proportion of outcome variation that lies between groups. Nevertheless, in order to reach the widest possible audience, in the current study we aimed to demonstrate the use traditional fixed effects regression within the AIHDA framework. In doing so, we hope to maximally increase the understanding and the application of AIHDA in current epidemiological

For peer review only - http://bmjopen.bmj.com/site/about/guidelines.xhtml

practice aimed to the quantitative evaluation of health care quality and public comparison in health care [3 10].

Implications and Conclusions

Dispensations of statins after AMI in Sweden are, according to the national quality indicator, closely but not fully met in relation to the benchmark value of 90%. However, neither regional nor sociodemographic differences in group averages seem to be major determinants of dispensation of statins. Therefore, any intervention aiming to reach the benchmark value should be universal. Sociodemographic differences nevertheless appeared more relevant than geographic differences. However, our results suggest that in spite the lower prevalence of statin prescription in some groups (e.g., women, especially to those with immigrant background and older than 65 years), the exclusive targeting of interventions to those groups would be unsuitable as many AMI patients lacking statin prescription are in the groups with higher prevalence of statin prescription. In other words, we cannot base decisions on stratum prevalence alone, we also need to consider the size of the strata

We need to clarify that our purpose was not to introduce a new statistical technique. Rather, our aim is to introduce an innovative strategy of analysis. By terming this strategy as “analysis of individual heterogeneity and discriminatory accuracy (AIHDA)” we underlie the importance of focusing on components of individual heterogeneity and discriminatory accuracy and not only on differences between group averages (as it is the

For peer review only - http://bmjopen.bmj.com/site/about/guidelines.xhtml

traditional approach). Within this strategy you can use any statistic technique like logistic or Poisson regression. We have also developed the AIHDA approach using multilevel models that we denominate MAIHDA [9] [5 8 15 17-19 40 41].

The challenge of this study is double. First, we aim to introduce a strategy of analysis that may discomfort the readers habituated with traditional focus on group mean differences [7]. Second, we aim to present this strategy of analysis in a correct but simplified way. The aim of our study is to provide a simple alternative for evaluation of inequalities in routine health care. Still the analytical approach and the way we use some concepts are innovative which may produce some unavoidable initial confusion.

A regular and public monitoring of geographic and sociodemographic inequalities in quality indicators is fundamental for the evaluation of health care quality. For this purpose, we need to apply state of the art statistical and epidemiological methods. The traditional mean-centric approach based on the analysis of differences between groups averages provides incomplete information that might mislead decision makers in healthcare. As illustrated in our study, AIHDA is not complicated to implement, and it provides more nuanced information than traditional public comparisons based only on group averages. We therefore propose the application of the AIHDA framework for the quantitative evaluation of healthcare quality when performing geographic and sociodemographic comparisons of quality indicators.

Footnotes

For peer review only - http://bmjopen.bmj.com/site/about/guidelines.xhtml

- **Contributors** JM had the initiative of the study and acquired the data. JM and JÖ wrote the original manuscript. RP-V and JÖ performed the analyses in coordination with JM, KK and GL. All the authors have contributed to the design of the study, JM and GL developed the methodology and directed the analyses. All authors have contributed to the interpretation of the results and drafting of the manuscript. All authors have revised the last version of the manuscript.

- **Funding** The Swedish Research Council (Vetenskapsrådet) supported this work through the project 'Multilevel Analyses of Individual Heterogeneity: innovative concepts and methodological approaches in Public Health and Social Epidemiology': (#2017-01321, PI: Juan Merlo).
- **Competing interests** None declared.
- **Ethics approval** Regional ethics committee Lund: ID: 2014/856. Data safety committees from the National board of health and welfare: ID 9542/2015. Statistics Sweden: ID 231424/878144-5.
- **Patient consent for publication** Not required.
- **Provenance and peer review** Not commissioned, externally peer reviewed.
- **Data availability statement** Data are available upon reasonable request. Original data are available from the Swedish National Board of Health and Welfare and Statistics, after approval of a research project by an Ethical Committee and by the data safety committees of Swedish Authorities.

For peer review only - http://bmjopen.bmj.com/site/about/guidelines.xhtml

References

1. Socialstyrelsen. God vård – om ledningssystem för kvalitet och patientsäkerhet i hälso- och sjukvården. In: Welfare SBoHa, ed., 2006:ISBN: 91-85482-05-6.
2. Heurgren M, Åberg A, Köster M, et al. Performance indicators in Swedish Health Care. *BMC Health Serv Res* 2007;7(A10) doi: ttps://doi.org/10.1186/1472-6963-7-S1-A10
3. Öppna jämförelser av hälso- och sjukvårdens kvalitet och effektivitet Jämförelser mellan landsting . *Sveriges Kommuner och Landsting wwwsklse/publikationer ISBN 978-91-7164-485-5* 2009
4. Sorman H. [Open comparisons of health care: "One of the most important contributions to modernization of health care"]. *Lakartidningen* 2010;107(32-33):1828-9. [published Online First: 2010/09/29]
5. Khalaf K, Axelsson Fisk S, Ekberg-Jansson A, et al. Geographical and sociodemographic differences in discontinuation of medication for Chronic Obstructive Pulmonary Disease - A Cross-Classified Multilevel Analysis of Individual Heterogeneity and Discriminatory Accuracy (MAIHDA). *Clin Epidemiol* 2020;12:783-96. doi: 10.2147/CLEP.S247368 [published Online First: 2020/08/09]
6. Merlo J, Mulinari S, Wemrell M, et al. The tyranny of the averages and the indiscriminate use of risk factors in Public Health: The case of coronary heart disease. *SSM - Population Health* 2017;3:684-98. doi: https://doi.org/10.1016/j.ssmph.2017.08.005 [published Online First: 2017.08.005]
7. Merlo J. Invited commentary: multilevel analysis of individual heterogeneity-a fundamental critique of the current probabilistic risk factor epidemiology. *Am J Epidemiol* 2014;180(2):208-12; discussion 13-4. doi: 10.1093/aje/kwu108
8. Merlo J, Wagner P, Leckie G. A simple multilevel approach for analysing geographical inequalities in public health reports: The case of municipality differences in obesity. *Health Place* 2019;58:102145. doi: 10.1016/j.healthplace.2019.102145 [published Online First: 2019/06/14]
9. Merlo J. Multilevel analysis of individual heterogeneity and discriminatory accuracy (MAIHDA) within an intersectional framework. *Soc Sci Med* 2018;203:74-80. doi: 10.1016/j.socscimed.2017.12.026 [published Online First: 2018/01/07]
10. Merlo J. Flernivåanalyser behövs i öppna jämförelser inom hälso- och sjukvård. *Läkartidningen 2019;116:FTEL* 2019
11. Evans CR, Erickson N. Intersectionality and depression in adolescence and early adulthood: A MAIHDA analysis of the national longitudinal study of adolescent to adult health, 1995-2008. *Soc Sci Med* 2019;220:1-11. doi: 10.1016/j.socscimed.2018.10.019
12. Mulinari S, Bredstrom A, Merlo J. Questioning the discriminatory accuracy of broad migrant categories in public health: self-rated health in Sweden. *Eur J Public Health* 2015;25(6):911-7. doi: 10.1093/eurpub/ckv099
13. Ljungman H, Wemrell M, Khalaf K, et al. Antidepressant use in Sweden: an intersectional multilevel analysis of individual heterogeneity and discriminatory accuracy (MAIHDA). *Scand J Public Health* 2021:1403494821993723. doi: 10.1177/1403494821993723 [published Online First: 2021/02/24]
14. Axelsson Fisk S, Lindstrom M, Perez-Vicente R, et al. Understanding the complexity of socioeconomic disparities in smoking prevalence in Sweden: a cross-sectional study applying intersectionality theory. *BMJ Open* 2021;11(2):e042323. doi: 10.1136/bmjopen-2020-042323 [published Online First: 2021/02/13]
15. Axelsson Fisk S, Mulinari S, Wemrell M, et al. Chronic Obstructive Pulmonary Disease in Sweden: An intersectional multilevel analysis of individual heterogeneity and discriminatory accuracy. *SSM Popul Health* 2018;4:334-46. doi: 10.1016/j.ssmph.2018.03.005
16. Wemrell M, Bennet L, Merlo J. Understanding the complexity of socioeconomic disparities in type 2 diabetes risk: a study of 4.3 million people in Sweden. *BMJ Open Diabetes Res Care* 2019;7(1):e000749. doi: 10.1136/bmjdrc-2019-000749 [published Online First: 2019/12/05]

For peer review only - http://bmjopen.bmj.com/site/about/guidelines.xhtml

17. Hernandez-Yumar A, Wemrell M, Abasolo Alesson I, et al. Socioeconomic differences in body mass index in Spain: An intersectional multilevel analysis of individual heterogeneity and discriminatory accuracy. *PLoS One* 2018;13(12):e0208624. doi: 10.1371/journal.pone.0208624
18. Persmark A, Wemrell M, Zettermark S, et al. Precision public health: Mapping socioeconomic disparities in opioid dispensations at Swedish pharmacies by Multilevel Analysis of Individual Heterogeneity and Discriminatory Accuracy (MAIHDA). *PLoS One* 2019;14(8):e0220322. doi: 10.1371/journal.pone.0220322 [published Online First: 2019/08/28]
19. Rodriguez-Lopez M, Merlo J, Perez-Vicente R, et al. Cross-classified Multilevel Analysis of Individual Heterogeneity and Discriminatory Accuracy (MAIHDA) to evaluate hospital performance: the case of hospital differences in patient survival after acute myocardial infarction. *BMJ Open* 2020;10(10):e036130. doi: 10.1136/bmjopen-2019-036130 [published Online First: 2020/10/26]
20. Ghith N, Frolich A, Merlo J. The role of the clinical departments for understanding patient heterogeneity in one-year mortality after a diagnosis of heart failure: A multilevel analysis of individual heterogeneity for profiling provider outcomes. *PLoS One* 2017;12(12):e0189050. doi: 10.1371/journal.pone.0189050
21. Evans CR, Leckie G, Merlo J. Multilevel versus single-level regression for the analysis of multilevel information: The case of quantitative intersectional analysis. *Soc Sci Med* 2020;245:112499. doi: 10.1016/j.socscimed.2019.112499 [published Online First: 2019/09/23]
22. Socialstyrelsen. Nationella riktlinjer för hjärtsjukvård Stöd för styrning och ledning. In: 2018-6-28| A, ed. Stockholm, Sweden, 2018.
23. Ruiz-Cantero MT, Blasco-Blasco M, Chilet-Rosell E, et al. Gender bias in therapeutic effort: from research to health care. *Farm Hosp* 2020;44(3):109-13. doi: 10.7399/fh.11394 [published Online First: 2020/05/27]
24. Chang AY, Abou-Arraj NE, Rodriguez F. Interventions to Reduce Ethnic and Racial Disparities in Dyslipidemia Management. *Current treatment options in cardiovascular medicine* 2019;21(5):24. doi: 10.1007/s11936-019-0725-4 [published Online First: 2019/05/09]
25. Wylie LE, Waterbrook AL, Dalen JE. Are Statins Indicated in Senior Citizens? A Review of Statin Therapy in the Elderly. *Am J Med* 2021 doi: 10.1016/j.amjmed.2021.11.009 [published Online First: 2021/12/03]
26. Hyun KK, Brieger D, Woodward M, et al. The effect of socioeconomic disadvantage on prescription of guideline-recommended medications for patients with acute coronary syndrome: systematic review and meta-analysis. *Int J Equity Health* 2017;16(1):162. doi: 10.1186/s12939-017-0658-z [published Online First: 2017/09/02]
27. Barros AJ, Hirakata VN. Alternatives for logistic regression in cross-sectional studies: an empirical comparison of models that directly estimate the prevalence ratio. *BMC Med Res Methodol* 2003;3:21. doi: 10.1186/1471-2288-3-21
28. Hosmer DW, Lemeshow S. Applied logistic regression. 2nd ed. New York: Wiley 2000.
29. Pepe M. S., Janes H., Longton G., et al. Limitations of the odds ratio in gauging the performance of a diagnostic, prognostic, or screening marker. *American journal of epidemiology* 2004;159(9):882-90. [published Online First: 2004/04/24]
30. Marmot M, Bell R. Fair society, healthy lives. *Public Health* 2012;126 Suppl 1:S4-S10. doi: 10.1016/j.puhe.2012.05.014 [published Online First: 2012/07/13]
31. Carey G, Crammond B, De Leeuw E. Towards health equity: a framework for the application of proportionate universalism. *Int J Equity Health* 2015;14:81. doi: 10.1186/s12939-015-0207-6
32. Merlo J, Wagner P, Ghith N, et al. An Original Stepwise Multilevel Logistic Regression Analysis of Discriminatory Accuracy: The Case of Neighbourhoods and Health. *PLoS One* 2016;11(4):e0153778. https://doi.org/10.1371/journal.pone.78. doi: https://doi.org/10.1371/journal.pone.0153778
33. Moons KG, de Groot JA, Bouwmeester W, et al. Critical appraisal and data extraction for systematic reviews of prediction modelling studies: the CHARMS checklist. *PLoS Med* 2014;11(10):e1001744. doi: 10.1371/journal.pmed.1001744

For peer review only - http://bmjopen.bmj.com/site/about/guidelines.xhtml

34. Ginsburg GS, Phillips KA. Precision Medicine: From Science To Value. *Health Aff (Millwood)* 2018;37(5):694-701. doi: 10.1377/hlthaff.2017.1624 [published Online First: 2018/05/08]
35. Ludvigsson JF, Andersson E, Ekbom A, et al. External review and validation of the Swedish national inpatient register. *BMC Public Health* 2011;11:450. doi: 10.1186/1471-2458-11-450 [published Online First: 2011/06/11]
36. Merlo J, Gerdtham UG, Lynch J, et al. Social inequalities in health – do they diminish with age? Revisiting the question in Sweden 1999. *Int J Equity Health* 2003;2(1):2.
37. Wemrell M, Karlsson N, Perez Vicente R, et al. An intersectional analysis providing more precise information on inequities in self-rated health. *Int J Equity Health* 2021;20(1):54. doi: 10.1186/s12939-020-01368-0 [published Online First: 2021/02/05]
38. Wagner P, Merlo J. Discriminatory accuracy of a random effect in multilevel logistic regression. *International Journal of Epidemiology* 2014;44:i49-i50.
39. Merlo J, Wagner P, Austin PC, et al. General and specific contextual effects in multilevel regression analyses and their paradoxical relationship: A conceptual tutorial. *SSM Popul Health* 2018;5:33-37. doi: 10.1016/j.ssmph.2018.05.006
40. Ljungman H, Wemrell M, Khalaf K, et al. Antidepressant use in Sweden: an intersectional multilevel analysis of individual heterogeneity and discriminatory accuracy (MAIHDA). *Scand J Public Health* 2022;50(3):395-403. doi: 10.1177/1403494821993723 [published Online First: 2021/02/24]
41. Zettermark S, Khalaf K, Perez-Vicente R, et al. Population heterogeneity in associations between hormonal contraception and antidepressant use in Sweden: a prospective cohort study applying intersectional multilevel analysis of individual heterogeneity and discriminatory accuracy (MAIHDA). *BMJ Open* 2021;11(10):e049553. doi: 10.1136/bmjopen-2021-049553 [published Online First: 2021/10/03]

For peer review only

For peer review only - http://bmjopen.bmj.com/site/about/guidelines.xhtml

Figure 1: Study population flow-chart

Figure 2: Crude prevalence (P) and 95% confidence intervals (CI) for use of statins 12 -18 months after hospital discharge with an acute myocardial infarction (AMI) diagnosis in patients aged 40-80 years in the period 2011-2013, across the 21 administrative regions of Sweden.

Figure 3: Crude prevalence (P) and 95% confidence intervals (CI) for use of statins 12 -18 months after hospital discharge with an acute myocardial infarction (AMI) diagnosis in patients aged 40-80 years in the period 2011-2013, across the 24 sociodemographic groups.

For peer review only

For peer review only - http://bmjopen.bmj.com/site/about/guidelines.xhtml

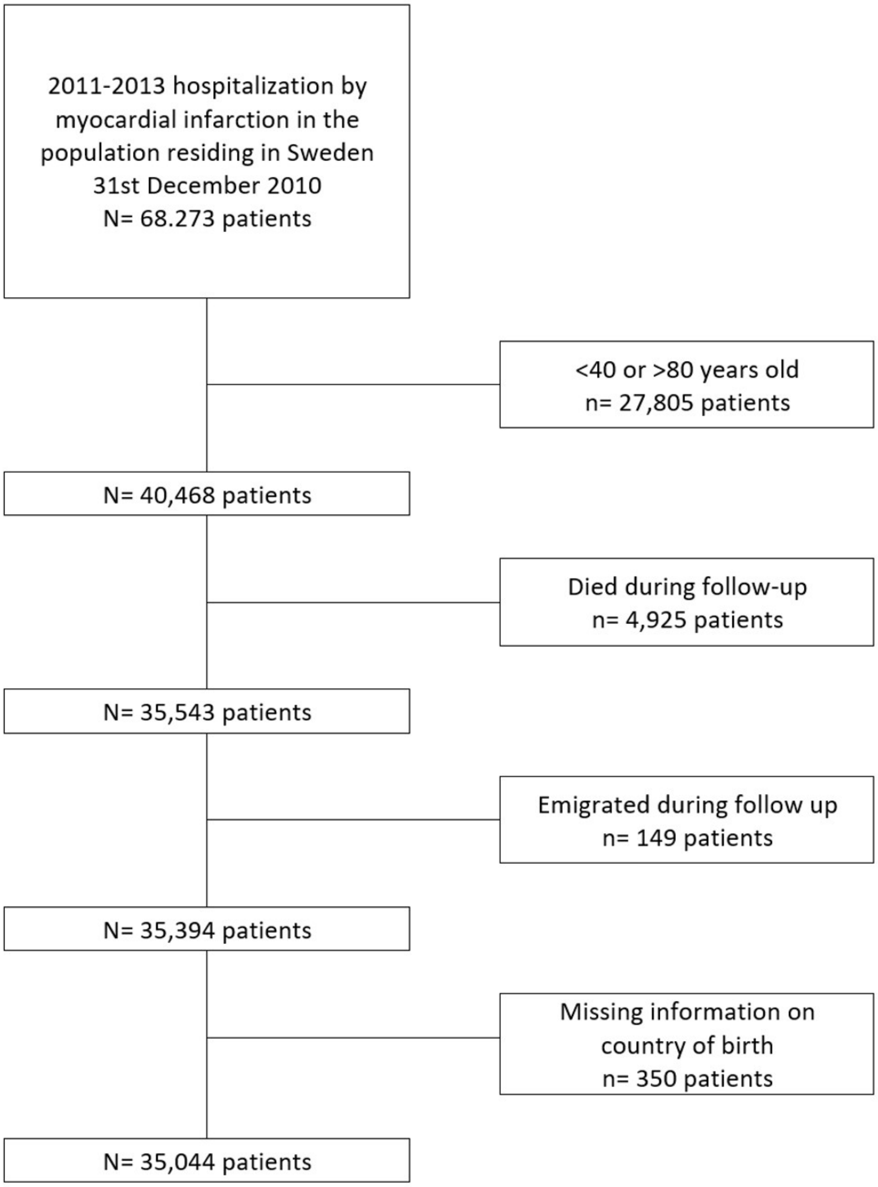

Figure 1: Study population flow-chart

261x348mm (96 x 96 DPI)

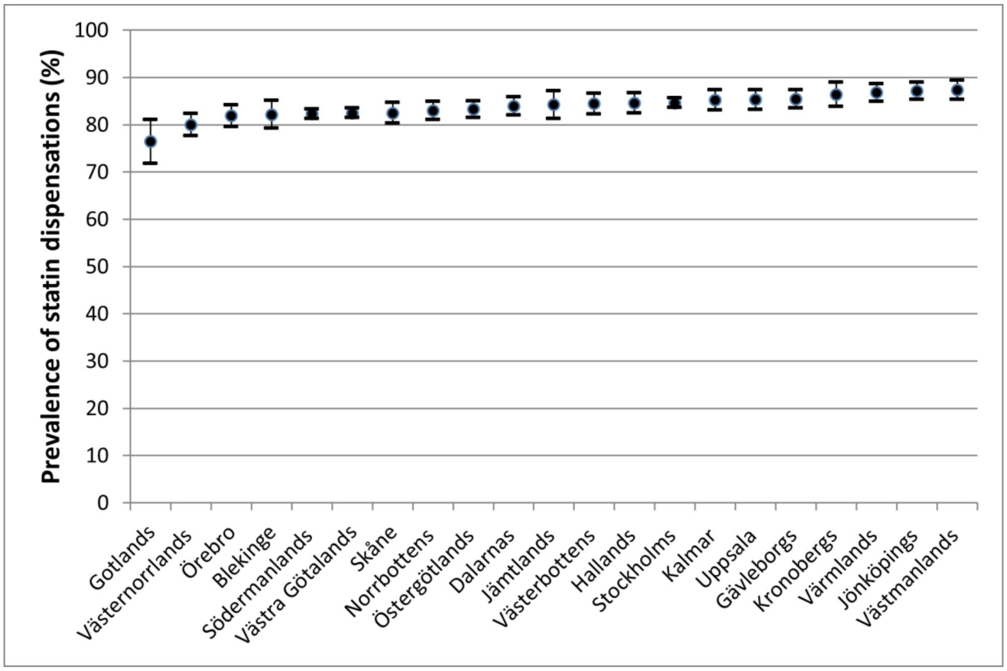

Figure 2: Crude prevalence (P) and 95% confidence intervals (CI) for use of statins 12 -18 months after hospital discharge with an acute myocardial infarction (AMI) diagnosis in patients aged 40-80 years in the period 2011-2013, across the 21 administrative regions of Sweden.

152x101mm (330 x 330 DPI)

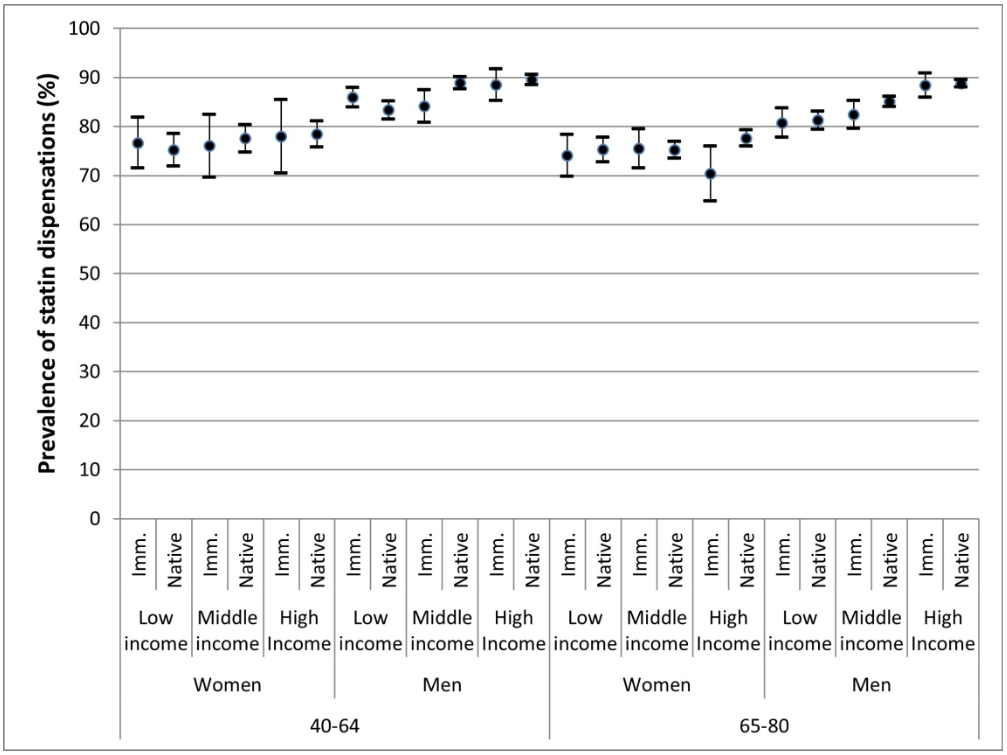

Figure 3: Crude prevalence (P) and 95% confidence intervals (CI) for use of statins 12 -18 months after hospital discharge with an acute myocardial infarction (AMI) diagnosis in patients aged 40-80 years in the period 2011-2013, across the 24 sociodemographic groups.

165x123mm (330 x 330 DPI)

SUPLEMENTARY MATERIAL (S)

S1: Supplementary analyses

SM 1. Prevalence Ratios (PR) and (95% confidence intervals) for use of statins 12-18 months after hospital discharge with an acute myocardial infarction (AMI) diagnosis in patients aged, 40-80 years old from 24 sociodemographic strata in the period 2011-2013

	Age	Sex	Income	Country of birth	All	Multicategorical *
Age						
40-64	1.03 (1.02-1.04)				1.02 (1.01-1.04)	
65-80	Reference					
Sex						
Women		Reference				
Men		1.14 (1.12-1.15)			1.13 (1.12-1.14)	
Income						
Low			Reference			
Middle			1.03 (1.01-1.04)		1.03 (1.01-1.04)	
High			1.07 (1.06-1.09)		1.06 (1.05-1.08)	
Country of birth						
Immigrant				Reference		
Native				1.03 (1.01-1.04)	1.01 (1.00-1.03)	
AUC	0.512	0.579	0.546	0.511	0.608	0.609

*Model 1B in table 2. AUC: area under the ROC curve

For peer review only

S2: Stata codes

Main analysis
```<br>. use cohort_selected.dta, clear<br><br>. generate time = 1<br>. stset time, failure(statins) id(lpnr)<br><br>* Model 1a<br>. stcox ib11.region, vce(robust)<br>. predict p1a<br><br>* Model 1b<br>. stcox ib12.inter, vce(robust)<br>. predict p1b<br><br>* Model 2<br>. stcox ib11.region ib12.inter, vce(robust)<br>. predict p2<br><br>. roccomp statins p1a p1b p2<br>```
Prevalences
```<br>. use cohort_selected.dta, clear<br>. by region, sort: egen cases = sum(statins)<br>. by region: gen total = _N<br>. keep cases total region<br>. duplicates drop<br><br>. use cohort_selected.dta, clear<br>. by inter, sort: egen cases = sum(statins)<br>. by inter: generate total = _N<br>. keep cases total inter<br>. duplicates drop<br><br>. use cohort_selected.dta, clear<br>. egen cases = sum(statins)<br>. display _N<br>. tabulate cases<br>```
Supplementary analysis
```<br>. use cohort_selected.dta, clear<br>```

For peer review only - http://bmjopen.bmj.com/site/about/guidelines.xhtml

```
. generate time = 1
. stset time, failure(statins) id(lpnr)

* Model 1b_age
. stcox ib1.age_cat, vce(robust)
. predict p2a

* Model 1b_sex
. stcox i.male, vce(robust)
. predict p2b

* Model 1b_income
. stcox i.cum, vce(robust)
. predict p2c

* Model 1b_country
. stcox i.Sw, vce(robust)
. predict p2d

* Model 1b_all
. stcox ib1.age_cat i.male i.cum i.Sw, vce(robust)
. predict p2e

. roccomp statins p2*
```


For peer review only - http://bmjopen.bmj.com/site/about/guidelines.xhtml

STROBE Statement—Checklist of items that should be included in reports of ***cross-sectional studies***

	Item No	Recommendation	Page No
**Title and abstract**	1	(*a*) Indicate the study's design with a commonly used term in the title or the abstract	1-2
		(*b*) Provide in the abstract an informative and balanced summary of what was done and what was found	2
**Introduction**			
Background/rationale	2	Explain the scientific background and rationale for the investigation being reported	3-4
Objectives	3	State specific objectives, including any prespecified hypotheses	3-4
**Methods**			
Study design	4	Present key elements of study design early in the paper	4, 7-9
Setting	5	Describe the setting, locations, and relevant dates, including periods of recruitment, exposure, follow-up, and data collection	5-6
Participants	6	(*a*) Give the eligibility criteria, and the sources and methods of selection of participants	5
Variables	7	Clearly define all outcomes, exposures, predictors, potential confounders, and effect modifiers. Give diagnostic criteria, if applicable	6
Data sources/ measurement	8*	For each variable of interest, give sources of data and details of methods of assessment (measurement). Describe comparability of assessment methods if there is more than one group	5-6
Bias	9	Describe any efforts to address potential sources of bias	6
Study size	10	Explain how the study size was arrived at	5
Quantitative variables	11	Explain how quantitative variables were handled in the analyses. If applicable, describe which groupings were chosen and why	6-8
Statistical methods	12	(*a*) Describe all statistical methods, including those used to control for confounding	7-10
		(*b*) Describe any methods used to examine subgroups and interactions	7-10
		(*c*) Explain how missing data were addressed	5
		(*d*) If applicable, describe analytical methods taking account of sampling strategy	
		(*e*) Describe any sensitivity analyses	
**Results**			
Participants	13*	(a) Report numbers of individuals at each stage of study—eg numbers potentially eligible, examined for eligibility, confirmed eligible, included in the study, completing follow-up, and analysed	5, 20
		(b) Give reasons for non-participation at each stage	5, 20
		(c) Consider use of a flow diagram	20
Descriptive data	14*	(a) Give characteristics of study participants (eg demographic, clinical, social) and information on exposures and potential confounders	10-12, 23-24
		(b) Indicate number of participants with missing data for each variable of interest	20
Outcome data	15*	Report numbers of outcome events or summary measures	10-12, 23-24

For peer review only - http://bmjopen.bmj.com/site/about/guidelines.xhtml

Main results	16	(*a*) Give unadjusted estimates and, if applicable, confounder-adjusted estimates and their precision (eg, 95% confidence interval). Make clear which confounders were adjusted for and why they were included	10-12, 23-24
		(*b*) Report category boundaries when continuous variables were categorized	5, 9
		(*c*) If relevant, consider translating estimates of relative risk into absolute risk for a meaningful time period	
Other analyses	17	Report other analyses done—eg analyses of subgroups and interactions, and sensitivity analyses	
**Discussion**			
Key results	18	Summarise key results with reference to study objectives	12-13
Limitations	19	Discuss limitations of the study, taking into account sources of potential bias or imprecision. Discuss both direction and magnitude of any potential bias	14-16
Interpretation	20	Give a cautious overall interpretation of results considering objectives, limitations, multiplicity of analyses, results from similar studies, and other relevant evidence	13-16
Generalisability	21	Discuss the generalisability (external validity) of the study results	15-16
**Other information**			
Funding	22	Give the source of funding and the role of the funders for the present study and, if applicable, for the original study on which the present article is based	16-17

*Give information separately for exposed and unexposed groups.

Note: An Explanation and Elaboration article discusses each checklist item and gives methodological background and published examples of transparent reporting. The STROBE checklist is best used in conjunction with this article (freely available on the Web sites of PLoS Medicine at http://www.plosmedicine.org/, Annals of Internal Medicine at http://www.annals.org/, and Epidemiology at http://www.epidem.com/). Information on the STROBE Initiative is available at www.strobe-statement.org.

For peer review only

BMJ Open

Geographic and sociodemographic differences in statin dispensation after acute myocardial infarction in Sweden – A register based prospective cohort study applying analysis of individual heterogeneity and discriminatory accuracy (AIHDA) for basic comparisons of healthcare quality.

Journal:	*BMJ Open*
Manuscript ID	bmjopen-2022-063117.R2
Article Type:	Original research
Date Submitted by the Author:	06-Apr-2023
Complete List of Authors:	Merlo, Juan; Lunds University Faculty of Medicine, Social Epidemiology<br>Öberg, Johan; Lund University Clinical Sciences, Unit for Social Epidemiology<br>Khalaf, Kani; Lund University, Department of Clinical Sciences<br>Perez-Vicente, Raquel; Lund University, Faculty of Medicine, Social Epidemiology<br>Leckie, George; University of Bristol, Multilevel Modelling
**Primary Subject Heading**:	Epidemiology
Secondary Subject Heading:	Epidemiology, Health services research
Keywords:	EPIDEMIOLOGY, Myocardial infarction < CARDIOLOGY, Quality in health care < HEALTH SERVICES ADMINISTRATION & MANAGEMENT, CLINICAL PHARMACOLOGY

SCHOLARONE™
Manuscripts

I, the Submitting Author has the right to grant and does grant on behalf of all authors of the Work (as defined in the below author licence), an exclusive licence and/or a non-exclusive licence for contributions from authors who are: i) UK Crown employees; ii) where BMJ has agreed a CC-BY licence shall apply, and/or iii) in accordance with the terms applicable for US Federal Government officers or employees acting as part of their official duties; on a worldwide, perpetual, irrevocable, royalty-free basis to BMJ Publishing Group Ltd ("BMJ") its licensees and where the relevant Journal is co-owned by BMJ to the co-owners of the Journal, to publish the Work in this journal and any other BMJ products and to exploit all rights, as set out in our licence.

The Submitting Author accepts and understands that any supply made under these terms is made by BMJ to the Submitting Author unless you are acting as an employee on behalf of your employer or a postgraduate student of an affiliated institution which is paying any applicable article publishing charge ("APC") for Open Access articles. Where the Submitting Author wishes to make the Work available on an Open Access basis (and intends to pay the relevant APC), the terms of reuse of such Open Access shall be governed by a Creative Commons licence – details of these licences and which Creative Commons licence will apply to this Work are set out in our licence referred to above.

Other than as permitted in any relevant BMJ Author's Self Archiving Policies, I confirm this Work has not been accepted for publication elsewhere, is not being considered for publication elsewhere and does not duplicate material already published. I confirm all authors consent to publication of this Work and authorise the granting of this licence.

For peer review only

Geographic and sociodemographic differences in statin dispensation after acute myocardial infarction in Sweden – A register based prospective cohort study applying analysis of individual heterogeneity and discriminatory accuracy (AIHDA) for basic comparisons of healthcare quality.

Juan Merlo[1, 2] ORCID: 0000-0001-8379-9708

Johan Öberg[1, 3] ORCID: 0000-0002-0701-5155

Kani Khalaf[1, 3] ORCID: 0000-0002-6749-7037

Raquel Perez Vicente[1] ORCID: 0000-0002-6273-1656

George Leckie[4] ORCID: 0000-0003-1486-745X

[1] Unit for Social Epidemiology, Faculty of Medicine, Lund University, Malmö, Sweden

[2] Centre for Primary Health Care Research, Region Skåne, Malmö, Sweden

[3] Department of Health and Medical Care Management, Region Skåne, Malmö, Sweden

[4] Centre for Multilevel Modelling, University of Bristol, UK

Corresponding author: Prof. Juan Merlo juan.merlo@med.lu.se, Skåne University Hospital, CRC, Jan Waldenströms gata 35 building 60, floor 12, SE-205 02 Malmö, Sweden, +46 70 5092265

Keywords (MeSH): Social Epidemiology, Health Care Quality Assessment, Pharmacologies, Health Services Evaluation, Epidemiological Methods

Word count: 5594

Abstract word count: 247

Number of references: 40

For peer review only

For peer review only

For peer review only - http://bmjopen.bmj.com/site/about/guidelines.xhtml

Abstract

Background: In Sweden, as in many other countries, official monitoring of healthcare quality is mostly focused on geographical disparities in relation to a desirable benchmark. However, current evaluations could be improved by considering: (i) the intersection of other relevant axes of inequity like age, sex, income, and migration status; and (ii) the existence of individual heterogeneity around averages. Therefore, using an established quality indicator (i.e., dispensation of statins after acute myocardial infarction, AMI), we valuate both geographical and sociodemographic inequalities and illustrate how the analysis of individual heterogeneity and discriminatory accuracy (AIHDA) enhances such evaluations.

Population and methods: We applied AIHDA and calculated the area under the ROC curve (AUC) of regional and sociodemographic differences in the statin dispensations of 35,044 patients from 21 Swedish regions and 24 sociodemographic strata who were discharged from the hospital with an AMI diagnosis between January 2011 and December 2013. Following the Swedish National Board of Health and Welfare, we used a benchmark value of 90%.

Results: Dispensation of stains after AMI in Sweden did not reach the desired target of 90%. Regional differences were absent/very small (AUC = 0.537) while sociodemographic differences were small (AUC = 0.618). Women, especially those with

For peer review only

immigrant background and older than 65 years, have the lowest proportions of statin dispensations after AMI.

Conclusions: As the AUC statistics are small, interventions trying to achieve the benchmark value should be universal. However, a special emphasis should nevertheless be directed towards women, especially older women with immigrant backgrounds.

For peer review only

For peer review only - http://bmjopen.bmj.com/site/about/guidelines.xhtml

Strengths and limitations of this study:

- The applied Analysis of Individual Heterogeneity and Discriminatory Accuracy (AIHDA) provides an improved mapping and quantification of geographical and sociodemographic inequalities in healthcare quality
- The quality of the data in our study is high as it is based on national registers with valid and standardized information on diagnoses, medication use and sociodemographic variables
- We can directly compare our results to those presented by public authorities, such as the Swedish National Boars of Health and Welfare, since the definition of the quality indicator is the same.
- The definition and categorization of the sociodemographic variables is coarse. However, more fine categorizations would excessively increase the number of strata
- The AIHDA approach can be implemented using both traditional regression or more advanced multilevel models which provide several advantages. However, traditional regression is a valid and accessible alternative

For peer review only

For peer review only - http://bmjopen.bmj.com/site/about/guidelines.xhtml

Introduction

In Sweden as in many other countries, policies and laws emanating from patient needs regulate and define standards of healthcare[1]. Good healthcare is defined as safe, accessible, delivered in time, health promoting, and knowledge- and evidence-based. In addition, to be fully efficient and effective, good healthcare must be equitable. That is, on equal terms and according to patients' needs. On this background, The Swedish National Board of Health and Welfare (NBHW) and the Swedish Association of Local Authorities and Regions (SALAR) define quality indicators[2] that can be compared against specific benchmarks. These quality indicators are regularly monitored for quality of performance assessment by means of simplified hospital and regional comparisons online (https://vardenisiffror.se/), and in reports such as *Open comparisons of healthcare performance*[3]. The benefits of monitoring for improving healthcare quality are many[4]. However, current comparisons could be improved for several reasons.

In spite of the current evidence on the existence of healthcare inequities conditioned by sociodemographic factors (e.g., age, sex, income, country of birth), today's official monitoring is mostly focused on geographical disparities, disregarding other relevant axes of possible inequity. Monitoring both geographic and sociodemographic inequalities seem necessary in order to improve the evaluation of quality of healthcare performance[5].

In addition, current comparisons are often based on the analysis and interpretation of differences between group averages, disregarding individual heterogeneity around such

For peer review only - http://bmjopen.bmj.com/site/about/guidelines.xhtml

averages[6 7]. This is a fundamental but often ignored question in epidemiology and healthcare evaluation [6-10]. To understand this concept we strongly suggest the reading of a previous publication[9] describing in detail this idea in non-technical terms. Shortly, when comparing group averages, we also need to quantify the extent of the groups' distributions of individual values (i.e., heterogeneity around averages). The size of this overlap needs be considered when evaluating differences between group averages[9].

Also, the criteria for quantifying the size of the group differences in official comparisons are not clearly stated. For instance, when can we say that there are large regional differences in a quality indicator? The criterion of "statistical significance" is not sufficient as very small and meaningless differences between group averages may nonetheless prove statistically significant if the sample is large enough. Therefore, measurable differences between group averages may exist together with considerable overlap of individual heterogeneity between groups, which may render the average differences irrelevant [9 11]. Therefore, group differences should not only be assessed by simple measures of differences between averages but also as the share of the total individual variance that is at the group level. Alternatively, we should quantify the accuracy of the information provided by the groups differences to classify persons according to the outcome.

The analysis of individual heterogeneity and discriminatory accuracy (AIHDA) we promote in this study, is an innovative analytical strategy that has the potential to address

For peer review only - http://bmjopen.bmj.com/site/about/guidelines.xhtml

the above criticisms and thereby improve the quantitative evaluation of health care quality. AIHDA is not a new statistical technique but rather a strategy of analysis that stress the importance of focusing on components of individual heterogeneity and discriminatory accuracy and not only on differences between group averages (as it is the traditional approach). AIHDA is proving an increasingly popular approach in public health and health care epidemiology [5 9 10 12-21]. When applying AIHDA, we do not consider differences between group averages and differences between patients as if they are two separate and unrelated phenomena of interest. Rather, we adopt a conceptually multilevel perspective that simultaneously considers both types of differences. From this standpoint, we evaluate sociodemographic and geographical group differences by quantifying the share of the total individual variation in patient outcomes that operates at the group level. In the case of binary patient outcomes (like most quality indicators), we can analogously evaluate the accuracy of the group level for discriminating individuals with the outcome from those without the outcome of interest. Applying AIHDA, we can evaluate the relevance of specific group differences (e.g., regional differences), and even compare the relative relevance of different grouping criteria (e.g., sociodemographic strata versus geographical regions).

AIHDA can be applied using traditional regression[13 15 17 22] or more advanced multilevel regression analyses (MLRA) that we denominate (M)AIHDA[5 9 10 12 14 16 18-20 23 24]. MLRA provide conceptual and methodological advantages as compared to traditional

For peer review only - http://bmjopen.bmj.com/site/about/guidelines.xhtml

regression analyses[25]. For instance, conceptually both kinds of analyses considers the groups as contexts but only MLRA explicitly models the groups as a second level (i.e., as a random effect using technical terminology). In MLRA the relative group differences (e.g., prevalence ratios) have the grand mean (i.e., the mean of the groups' means) as reference while in traditional analyses the reference is a specific group (as in our present study). MLRA can better handle groups with few individuals via providing reliability weighted estimations.

While we primarily recommended the use of multilevel models[25], for simplified routine monitoring and online presentations, traditional regression analysis provides a valid AIHDA alternative. However, independently of the statistical technique, we want to stress that we consider the groups (e.g., regions or socioeconomic strata) as contexts rather than as characteristics attached to the individuals. That is, conceptually we adopt multilevel perspective that partitions the individual differences into within groups (level 1) and between groups (level 2).

The challenge of the present study is double. First, we aim to introduce a strategy of analysis that may discomfort the readers habituated with traditional focus on group mean differences[7]. Second, we aim to present this strategy in a correct but easy way. Our intention is to provide a simple alternative for evaluation of inequalities in routine health care. Still the analytical approach and the way we use some concepts are innovative which may need some extra contemplation. We provide suitable references for the

For peer review only - http://bmjopen.bmj.com/site/about/guidelines.xhtml

readers who will a deeper understanding of both AIHDA [13 15 17 22] and (M)AIHDA[5 9 10 12 14 16 18-20 23 24]

Motivated by the above background, we illustrate how the AIHDA approach can be applied for public monitoring of geographical as well as sociodemographic and differences in statin treatment after hospitalization for acute myocardial infarction (AMI), which is a well-known process indicator specifically adopted by the Swedish NBHW[26]. We analyze both regional[4] and sociodemographic differences as previous research has shown less dispensations in women[27], immigrants[28], elderly people[29] and in patients with low socioeconomic position[30]. For this purpose, we analyze the statins dispensations of 31,027 patients discharged from the hospital with an AMI diagnosis between January 2011 and December 2013.

Population and methods

Databases

Our investigation is based on a record-linkage database of several registers with national coverage, including the Swedish Population Register (TPR) and the Longitudinal Integration Database for Health Insurance and Labour Market Studies (LISA), administrated by Statistics Sweden (SCB), as well as the National Patient Register (NPR), the Cause of Death Register, and the Swedish Prescribed Drug Register (SPDR) administrated by the NBHW. The NPR records both outpatient clinic and inpatient

For peer review only - http://bmjopen.bmj.com/site/about/guidelines.xhtml
For peer review only

discharges from hospital with diagnoses coded according to the International Classification of Diseases, 10th edition (ICD-10). However, it does not cover information on diagnoses in primary health care. The SPDR contains information on all prescription drug dispensations from Swedish pharmacies. To maintain confidentiality, the registers were linked using an arbitrary serial number assigned to each individual by statistics Sweden instead of the Swedish unique personal identification number .

The Regional Ethics Review Board in Southern Sweden and the data safety committees from the NBHW and from Statistics Sweden approved the construction of the database.

Study population

We defined the study population using the same criteria as applied by the Swedish NBHW for evaluating the quality indicator on statins dispensation after hospital discharge for AMI.

The initial population included all 40,468 patients aged 40-80 years, who were residing in Sweden by 31 December 2010, and were discharged from hospital with an *AMI diagnosis* (ICD-10: I21 or I22) between 1 January 2011 and 31 December 2013. We followed each patient between the 13th and 18th month after discharge from the index AMI hospitalization regarding their statin dispensation from the pharmacies. We excluded 4,925 patients who died between discharge from hospital and end of follow-up,149 patients who emigrated, and 350 patients with missing information on country of birth. The final study population included 35,044 patients. See figure 1 for details. The

For peer review only - http://bmjopen.bmj.com/site/about/guidelines.xhtml

selection criteria are in concordance with those used by the Swedish authorities to define the quality indicator. Therefore, we do not question the validity of indicator, but just present a way of analysing it,

Assessment of variables

We defined the *quality indicator* according to the Swedish authorities as at least 90% of patients aged 40-80 years with a hospital discharge diagnosis of AMI and as having had at least one statin dispensation between the 13th and 18th month period after the hospital discharge[26]. We define *statin use* as any recorded dispensation of statin or a statin-combination drug (ATC codes C10AA or C10BA) in the SPDR during the follow-up period.

We classified the patients into the 21 *administrative regions* of Sweden according to their legal residence address and used Region Skåne as the reference region in the comparisons. Administrative regions in Sweden form autonomous political units and act as principals for healthcare.

We categorized *age* into two groups: 40-64 years and 65-80. *Sex* was defined as male or female according to the registered information. We categorized patients according to their *country of birth* into native (i.e., born in Sweden) or not (i.e., immigrant).

We obtained information on *individualised disposable family income* for the years 2000, 2005 and 2010 to compute a cumulative income measure that considers the size of the

For peer review only - http://bmjopen.bmj.com/site/about/guidelines.xhtml

household and the consumption weight of the individuals according to Statistics Sweden. For each of the three years, income levels were categorised into 25 quantiles using the complete Swedish population. These groups from the respective three years were summed up, so that each individual received a value between 3 (always in the lowest income group) and 75 (always in the highest income group). We categorised this cumulative income measure into three groups by tertiles (low, medium, or high income). Individuals with missing values on income during 2000 or 2005 were assigned the tertile values for the year 2010. No individuals had missing income data for 2010. By calculating a cumulative income measure, we obtain a more stable measurement of socioeconomic position.

Finally, we constructed a *sociodemographic multicategorical variable* consisting of all possible combinations of the categories of the four sociodemographic variables age, sex, income, and immigration status. This resulted in 24 strata.

Patient and public involvement

Because of the nature of our study, patient and public involvement is necessary. We will communicate the results of our study to politicians and healthcare professionals, who represent the public's interest.

For peer review only - http://bmjopen.bmj.com/site/about/guidelines.xhtml

Statistical and epidemiological analysis

To examine the association between geographical and sociodemographic variables and the use of statins, we calculated the crude percentage of patients who were dispensed statins in each region and in each multicategorical sociodemographic strata.

Next, we constructed three regression models following the AIHDA approach. Since the prevalence of the outcome was not extreme, we measured the associations between use of statins and the explanatory variables by prevalence ratios (PRs) and their 95% confidence intervals (CI) rather than by odds ratios. For this purpose, we applied Cox proportional hazards regression models with a constant follow-up time equal to 1 and report heteroskedasticity robust confidence intervals [31] using the *vce(robust)* option in Stata (see S2).

Model 1A estimates geographical inequalities by including only the geographical variable with the 21 regions, specifying Region Skåne as reference in the comparisons.

Model 1B estimates sociodemographic inequalities by including the sociodemographic multicategorical variable only, specifying the strata of native 40-64 years old men with high income as the reference category. The focus of our study was the sociodemographic multicategorical variable, but for informative reasons we performed a

For peer review only - http://bmjopen.bmj.com/site/about/guidelines.xhtml

series of intermediate models one variable in turn from the set of variables used to construct the multicategorical variable (see supplementary information S1).

Model 2 enters the geographical variable and the sociodemographic multicategorical variable simultaneously. This model estimates the geographical and sociodemographic differences adjusted for each other.

In order to evaluate and compare the relevance of the sociodemographic and geographical information for understanding use of statins after AMI, we estimated the Discriminatory accuracy (DA) for each model by calculating the area under the receiver operating characteristic curve (AUC) with 95% confidence intervals (CIs). The AUC is obtained by plotting the true-positive fraction against the false-positive fraction for different thresholds of the predicted probability of statin use. The value of the AUC ranges from .5 to 1, with .5 indicating the model has no predictive accuracy and 1 representing perfect discrimination. Using the criteria proposed by Hosmer and Lemeshow [32], we classified DA as absent or very small ($.5 \leq AUC \leq .6$), small ($.6 < AUC \leq .7$), large ($.7 < AUC \leq 0.8$), or very large ($AUC > .8$). As explained in previous publications [9 11], measuring geographical and sociodemographic differences to evaluate healthcare quality is analogous to performing a diagnostic test [33]. No decision-maker in healthcare would want to use such a test without first knowing its discriminatory accuracy (i.e., sensitivity, specificity, AUC). We do not want to unnecessarily treat false positive individuals or incorrectly deny treatment of false negative patients. In our case, we want

For peer review only - http://bmjopen.bmj.com/site/about/guidelines.xhtml

to know the accuracy of public sociodemographic and geographical comparisons for discriminating individuals with a positive result of the quality indicator (i.e., use of statins) from those with a negative result (i.e., no statins).

Strategy for evaluating sociodemographic and geographical differences

Applying the AIHDA framework means that we focus on both the group averages (that is, the region and sociodemographic strata averages) and the individual heterogeneity around these averages (that is, the AUC value, which informs on the overlap in individual heterogeneity across groups).

This framework includes four steps to achieve a complete analysis of inequalities. However, the approach may be modified for different research questions[9].

In the *first step (i)* we identify the quality indicator and the target or benchmark average value as we described above. In the *second step (ii)* we estimate and visualize the groups' differences using appropriate plots (e.g., caterpillar plots) and in the *third step (iii)* we quantify the size of these differences. That is, for a correct interpretation of the plots, the size of the groups' differences needs to be evaluated using a measure of discriminatory accuracy like the AUC. In traditional comparisons of group averages, the criteria for quantifying the size of the geographical and sociodemographic differences are not clearly stated. However, in the AIHDA framework we evaluate geographical and sociodemographic differences by quantifying the share of the total

For peer review only - http://bmjopen.bmj.com/site/about/guidelines.xhtml

variation in individual outcomes that operates at the group level (i.e., regions, sociodemographic strata). In the case of binary individual health outcomes, we can use the AUC as it provides information expressed in terms of discriminatory accuracy. Finally, in the *fourth step (iv)* we proceed to the interpretation of the results. Following the study referred earlier[9], we present a practical framework for evaluating geographical and sociodemographic differences. For this purpose, we need at least two types of information.

First, we need to know whether the average quality indicator (i.e., percentage of patients on statins after AMI) has reached the predetermined target value fully (i.e., ≥ 90% of all AMI patients are on statins), closely (i.e., 80-89%), or insufficiently (i.e., < 80%). The criteria we use to qualify the prevalence value as “fully” is based on the definition given by Swedish NBHW. However, the qualifications "closely" and “insufficiently” are ours and therefore subjective. More generally, the thresholds defining these categories will differ across quality indicators.

Second, we need information on the *size of the group differences* as assessed by the AUC, which expresses the accuracy of the differences between group averages for discriminating patients with the outcome from those without the outcome. These two types of information need to be combined for the evaluation. Box 1 illustrates these ideas by means of a simple framework with 12 scenarios that can be used to orient the interpretation of an analysis.

For peer review only - http://bmjopen.bmj.com/site/about/guidelines.xhtml

For peer review only

For peer review only - http://bmjopen.bmj.com/site/about/guidelines.xhtml

Box 1. Framework for evaluating geographical and sociodemographic differences in a specific quality indicator. For this purpose, we need information about the *target indicator value*. That is, whether the average quality indicator (i.e., percentage of patients on statins after AMI) has "insufficiently", "closely" or "fully" reached a predetermined target *benchmark* level. We also need information on the area under receiver operating characteristics curve (AUC), which expresses the accuracy of the differences between group averages for discriminating patients with the outcome from those without the outcome. Combining this information, we obtain 12 different scenarios useful for the evaluation.

		**Target indicator value reached**		
**Size of the group differences**		Insufficiently	Closely	Fully
Absent/very small	.5 ≤ AUC ≤ .6	A	B	C
Small	.6 ≤ AUC < .7	D	E	F
Large	.7 ≤ AUC < .8	G	H	I
Very large	.8 ≤ AUC	J	K	L

In the ideal scenario (scenario C) the desired target level has been fully reached in the population of patients, and the group differences are absent or very small. The conclusion would be that all groups have been treated appropriately and on equal terms. From this perspective, the quality of the healthcare is high.

In the worst scenario (scenario A) the desired target level has not been achieved in the population of patients, and the groups differences are again absent. The conclusion would be that all groups have been treated on equal terms but inadequately. That is, the quality of healthcare in the whole population is low.

For peer review only - http://bmjopen.bmj.com/site/about/guidelines.xhtml

Observe that in both scenarios A and C interventions targeted to specific groups are not justifiable. Rather any intervention should be universal (i.e., directed to the whole country). In scenario C, the reason for the intervention would be to maintain the desirable value of the quality indicator in all groups. In the scenario A, the reason would be to reach this desirable level in all the groups.

The interpretation of the scenarios in the lowest corners of the table (J and L) is conditioned by the very large size of the group differences. For example, in scenario L even though the population as a whole has achieved the target level, some groups may not have done so. In contrast, in scenario J, some groups may have achieved the target level even though the population as a whole has not achieved it. In these scenarios (J and L) targeted group interventions are justified.

The framework we propose fits well with the concept of *proportionate universalism* for resource allocation in public health [34 35]. That is, health care actions must be universal, not targeted, but with a scale and intensity that is proportionate to the level of disadvantage. The AIHDA approach we apply in this study can be used to inform decisions regarding the appropriate scale and intensity for a given sociodemographic or geographic context.

For peer review only - http://bmjopen.bmj.com/site/about/guidelines.xhtml

Results

The overall prevalence of statin dispensations in AMI patients 12-18 months after discharge was 83.7% (29,231/35,044). Therefore, the benchmark value of 90% was *closely* but not fully met on a national level according to the framework in Box 1.

Figure 2 and Table 1 illustrate the prevalence of statin dispensations in each administrative region in Sweden. Västmanland has the highest prevalence of statin use at 87.4% while Gotland has the lowest prevalence at 76.4%. None of the regions reach the benchmark value of 90%. The relative differences when comparing with the region of Skåne (Model 1A) were tiny and Table 1 illustrates that only four regions (i.e., Gotland, Västernorrland, Stockholm and Västra Götaland) presented a slightly but conclusively lower PR than Skåne. No other region showed a conclusively higher PR than Skåne. The PR adjusted for the multicategorical sociodemographic variable (Model 2) were very similar to the unadjusted PR (Model 1A), indicating that the sociodemographic composition of the regions did not confound the geographical differences.

Table 1. Number of patients using statins (n), number of individuals (N), crude prevalence (P) and 95% confidence intervals (CI), unadjusted prevalence ratios (PR), and prevalence ratios adjusted for sociodemographic variables (PRa) for use of statins 12 -18 months after hospital discharge with an acute myocardial infarction (AMI) diagnosis in patients aged 40-80 years from the 21 Swedish geographical administrative regions in the period 2011-2013.

				Model 1A	Model 2
	n	N	P (95% CI) %	PR (95% CI)	PRa (95% CI)

For peer review only - http://bmjopen.bmj.com/site/about/guidelines.xhtml

Sweden	29321	35044	83.67 (83.28-84.06)	–	–
Administrative Regions					
Gotland	246	322	76.40 (71.76-81.04)	0.90 (0.85-0.96)	0.91 (0.85-0.97)
Västernorrland	922	1,153	79.97 (77.65-82.28)	0.95 (0.92-0.98)	0.94 (0.92-0.97)
Örebro	886	1,082	81.89 (79.59-84.18)	0.97 (0.94-1.00)	0.97 (0.94-1.00)
Blekinge	525	639	82.16 (79.19-85.13)	0.97 (0.93-1.01)	0.98 (0.94-1.02)
Stockholm	4,477	5,440	82.30 (81.28-83.31)	0.97 (0.96-0.99)	0.97 (0.95-0.98)
Västra Götaland	4,391	5,326	82.44 (81.42-83.47)	0.97 (0.96-0.99)	0.97 (0.96-0.99)
Uppsala	960	1,164	82.47 (80.29-84.66)	0.97 (0.95-1.00)	0.97 (0.94-1.00)
Norrbotten	1,215	1,465	82.94 (81.01-84.86)	0.98 (0.95-1.01)	0.98 (0.95-1.00)
Östergötland	1,400	1,682	83.23 (81.45-85.02)	0.98 (0.96-1.01)	0.98 (0.96-1.01)
Dalarna	1212	1,444	83.93 (82.04-85.83)	0.99 (0.97-1.02)	0.99 (0.97-1.02)
Jämtland	501	595	84.20 (81.27-87.13)	1.00 (0.96-1.03)	1.00 (0.96-1.03)
Västerbotten	893	1,058	84.40 (82.22-86.59)	1.00 (0.97-1.03)	0.99 (0.97-1.02)
Halland	940	1,112	84.53 (82.41-86.66)	1.00 (0.97-1.03)	1.00 (0.97-1.02)
Skåne	4,085	4,828	84.61 (83.59-85.63)	Reference	Reference
Kalmar	898	1,054	85.20 (83.06-87.34)	1.01 (0.98-1.04)	1.01 (0.98-1.04)
Södermanland	973	1,141	85.28 (83.22-87.33)	1.01 (0.98-1.04)	1.01 (0.98-1.04)
Gävleborg	1,126	1,318	85.43 (83.53-87.34)	1.01 (0.98-1.04)	1.01 (0.99-1.04)
Kronoberg	592	685	86.42 (83.86-88.99)	1.02 (0.99-1.05)	1.02 (0.99-1.05)
Värmland	1,076	1,240	86.77 (84.89-88.66)	1.03 (1.00-1.05)	1.03 (1.00-1.05)
Jönköping	1,126	1,292	87.15 (85.33-88.98)	1.03 (1.01-1.06)	1.03 (1.00-1.05)
Västmanland	877	1,004	87.35 (85.29-89.41)	1.03 (1.01-1.06)	1.04 (1.01-1.06)
Area under the ROC curve (AUC)				0.537	0.618

Figure 3 and Table 2 illustrate the crude prevalence of statin dispensations for each sociodemographic stratum, with the highest prevalence (i.e., 89.5%) in the reference strata of 40–64-year-old native men with high income, followed by the strata of 40–64-year-old native men with middle income (i.e., 88.8%). We found the lowest prevalence of statin use in the stratum of 65–80-year-old immigrant women with high income (i.e., 70.3%), closely followed by 65–80-year-old immigrant women with low income (i.e., 74.1%). Only the reference sociodemographic stratum almost grasped the target value of

For peer review only

90% (i.e., 89.5%). None of the female strata were close to reaching the target. In

Table 2. Number of patients using statins (n), number of individuals (N), crude prevalence (P) and 95% confidence intervals (CI), unadjusted prevalence ratios (PR), and prevalence ratios adjusted for administrative regions (PRa) for use of statins 12-18 months after hospital discharge with an acute myocardial infarction (AMI) diagnosis in patients aged, 40-80 years old from 24 sociodemographic strata in the period 2011-2013

				Model 1B	Model 2
	n	N	P (95% CI) %	PR (95% CI)	PRa (95% CI)
Sweden	29,321	35,044	83.67 (83.28-84.06)	–	–
Women					
65-80 , High income, Immigrant	180	256	70.31 (64.72-75.91)	0.79 (0.72-0.85)	0.79 (0.73-0.85)
65-80 , Low income, Immigrant	300	405	74.07 (69.81-78.34)	0.83 (0.78-0.88)	0.83 (0.78-0.88)
65-80 , Middle income, Native	1,936	2,575	75.18 (73.52-76.85)	0.84 (0.82-0.86)	0.84 (0.82-0.86)
40-64 , Low income, Native	479	637	75.20 (71.84-78.55)	0.84 (0.80-0.88)	0.84 (0.80-0.88)
65-80 , Low income, Native	852	1,132	75.27 (72.75-77.78)	0.84 (0.81-0.87)	0.84 (0.81-0.87)
65-80 , Middle income, Immigrant	338	448	75.45 (71.46-79.43)	0.84 (0.80-0.89)	0.84 (0.80-0.89)
40-64 , Middle income, Immigrant	130	171	76.02 (69.62-82.42)	0.85 (0.78-0.92)	0.85 (0.78-0.93)
40-64 , Low income, Immigrant	197	257	76.65 (71.48-81.83)	0.86 (0.80-0.92)	0.86 (0.80-0.92)
40-64 , Middle income, Native	656	846	77.54 (74.73-80.35)	0.87 (0.83-0.90)	0.86 (0.83-0.90)
65-80 , High income, Native	1,838	2,369	77.59 (75.91-79.26)	0.87 (0.85-0.89)	0.87 (0.85-0.89)
40-64 , High income, Immigrant	92	118	77.97 (70.49-85.44)	0.87 (0.79-0.96)	0.87 (0.79-0.96)
40-64 , High income, Native	709	904	78.43 (75.75-81.11)	0.88 (0.85-0.91)	0.87 (0.84-0.91)
Men					
65-80 , Low income, Immigrant	548	679	80.71 (77.74-83.67)	0.90 (0.87-0.94)	0.90 (0.87-0.94)
65-80 , Low income, Native	1,409	1,735	81.21 (79.37-83.05)	0.91 (0.88-0.93)	0.91 (0.88-0.93)

contrast, the male strata were often much closer to reaching the target. Table 2 also presents the unadjusted PR (Model 1B), and the PR adjusted for the geographical variable (Model 2), with the strata of 40–64-year-old high-income native men as reference stratum. As many as 19 sociodemographic strata show conclusively smaller PRs than the reference. The adjusted PR were very similar to the unadjusted PR indicating that geographical factors did not confound the sociodemographic differences.

For peer review only

For peer review only - http://bmjopen.bmj.com/site/about/guidelines.xhtml

65-80 , Middle income, Immigrant	565	686	82.36 (79.51-85.21)	0.92 (0.89-0.95)	0.92 (0.89-0.95)
40-64 , Low income, Native	1,283	1,540	83.31 (81.45-85.17)	0.93 (0.91-0.95)	0.93 (0.91-0.95)
40-64 , Middle income, Immigrant	397	472	84.11 (80.81-87.41)	0.94 (0.90-0.98)	0.94 (0.90-0.98)
65-80 , Middle income, Native	3,791	4,458	85.04 (85.04-85.04)	0.95 (0.93-0.97)	0.95 (0.93-0.96)
40-64 , Low income, Immigrant	1,006	1,171	85.91 (83.92-87.90)	0.96 (0.94-0.98)	0.96 (0.94-0.99)
65-80 , High income, Immigrant	570	645	88.37 (88.37-88.37)	0.99 (0.96-1.02)	0.99 (0.96-1.02)
40-64 , High income, Immigrant	337	381	88.45 (85.24-91.66)	0.99 (0.95-1.03)	0.99 (0.95-1.03)
65-80 , High income, Native	6,303	7,101	88.76 (88.76-88.76)	0.99 (0.98-1.01)	0.99 (0.98-1.00)
40-64 , Middle income, Native	2,240	2,522	88.82 (87.59-90.05)	0.99 (0.97-1.01)	0.99 (0.97-1.01)
40-64 , High income, Native	3,165	3,536	89.51 (88.50-90.52)	Reference	Reference
Area under the ROC curve (AUC)				0.609	0.618

Model 1A, which includes information about administrative regions, presented an AUC value of 0.537. This AUC value indicates that the geographical differences were *absent/very small,* and that region provides very limited information about statin dispensations. Model 1B, which includes information on the sociodemographic strata, showed an AUC of 0.609 which indicates that the sociodemographic differences were *small*. The sociodemographic AUC was 0.072 units higher than the AUC of the geographical model 1A. Model 2, which includes both geographical and sociodemographic information, presented an AUC value of 0.618, just 0.008 higher than that presented by model 1B. In the context of our analysis, these findings indicates that sociodemographic information is relatively more relevant than geographic information. In summary, the target indicator value was *closely* reached in Sweden as a whole. The geographical analysis indicated *absent/very small* differences, which corresponds with scenario B in Box 1. The sociodemographic analysis indicated *small* differences, which corresponds with the scenario E in Box 1.

For peer review only - http://bmjopen.bmj.com/site/about/guidelines.xhtml

In relation to the sociodemographic differences, the complementary analysis (S1) showed that the AUC was very small when we fitted separate models which included only one variable at a time. The highest AUC was for the variable sex (AUC = 0.579) which was 0.029 units lower than the AUC for all variables together (AUC = 0.608). We also observed a very small interaction of effects in the model with the multicategorical variable as when comparing with model with all the single variables together, the AUC of the model with the multicategorical variable only increases 0.001 units.

Discussion

We evaluated regional and sociodemographic inequalities in statin treatment after AMI. When doing so, we also aimed to demonstrate how AIHDA provides enhanced information for evaluating healthcare inequalities [9 36]. While traditional public comparisons focus on differences between group averages, AIHDA considers both differences between group averages and the individual heterogeneity around those averages as expressed by their discriminatory accuracy. AIHDA informs on the share of the total individual variation in patient outcomes that operates at the group level. That is, to which degree the group-level information is able to discriminate with accuracy patients with, from those without the indicator under study[9]. The idea of discriminatory accuracy is fundamental in many fields of epidemiology not only when evaluating statistical model

For peer review only - http://bmjopen.bmj.com/site/about/guidelines.xhtml

prediction,[37] but also in the evaluation of diagnostic and prognostic tests[33], as well as risk factors in public health[6] and in the study of geographical[36] and socioeconomic[19] determinates of health. It is also a cornerstone in current precision medicine[38]. Nevertheless, these concepts need wider adoption still and we refer elsewhere for a longer explanation[6 7 9 10].

The overall prevalence of statin treatment in our study population was 83.7% which while lower was judged high enough to have “closely” reached the target benchmark value of 90%. We observed regional and sociodemographic differences in the prevalence of statin treatment after AMI. However, the low AUC values indicated that the differences were “absent/very small” (AUC = 0.537) for the administrative regions and “small” (AUC = 0.609) for the sociodemographic groups. Combining both regional and sociodemographic information did not substantially increase the AUC of the sociodemographic information alone (AUC = 0.618). That is, the sociodemographic differences seem to play a modest but more relevant role than the regional differences for understanding inequalities in statin treatment after AMI.

Our analysis also shows that women in all strata have a lower prevalence of statin dispensations after AMI than men, which is in line with previous research indicating the existence of gender inequalities [27]. However, those inequalities need be interpreted in the light of the small DA of the sociodemographic strata. In fact, while the prevalence of using statins is higher among men, the fact that AMI patients are disproportionately male means that majority of non-statin users are nevertheless also men.

For peer review only - http://bmjopen.bmj.com/site/about/guidelines.xhtml

According to the framework presented in box 1, the sociodemographic differences would be placed in scenario E. That is, the target value was closely but not fully reached and there were small differences between the sociodemographic strata. Accordingly, any strategy aimed to improve the outcome of the quality indicator should be universal but proportionately more intense among sociodemographic groups with increased risk not using statins. Analogously, the differences between administrative regions would be placed in the scenario B. In this case we should recommend universal interventions without targeting any specific regions.

Strengths and limitations

In addition to an improved and more nuanced analysis, AIHDA conveys further strengths as compared to traditional studies exclusively based on differences between group averages. It is known that epidemiological studies comparing group averages may *stigmatize* the individuals belonging to groups with "bad" average values and create *false expectations* for the individuals belonging to groups with "good" average values. This situation is true for both traditional and AIHDA studies and it is an inevitable side effect in epidemiology. Nevertheless, the perils of stigmatization and false expectations are often ethically justifiable because the aim of the epidemiological analyses is to improve the health and heath care of the individuals. However, knowing the DA of the group comparisons allows us to decide whether pointing out specific population groups is justifiable or not. If the DA is high, pointing out specific population groups is justifiable.

For peer review only - http://bmjopen.bmj.com/site/about/guidelines.xhtml

However, if the DA is low, the traditional emphasis on differences between group averages should be avoided. For instance, our results show that, because the AUC value is low, differences between administrative regions are less relevant for understanding individual statin dispensations. Therefore, simplistic interpretation based on visual analyses of figures like the caterpillar plot in our study (Figure 2) should be sidestepped.

By overlooking the DA of the group-averages, traditional studies may simultaneously lead to both under and overtreatment if a possible intervention is based on group information only. If the DA is low, many individuals taking statins but belonging to a group with a “bad” average (i.e., false positives cases) could be exposed to the intervention while many individuals not taking statins but belonging to a group with a “good” average (i.e., false negative cases) would not receive any intervention. For instance, using the information in Table 1, we can see that the region of Gotland has the worst average value (i.e., 76.4%), meaning that 76 of the 322 patients were not dispensed statins. However, in the region of Västmanland with the best average value (i.e., 87.4 %), this figure was 127 of the 1004 patients since Västmanland has a larger population. That is, the “best” region has 1.7 (= 127/76) times more patients lacking statin treatment that the “worst” region. It is correct that the region of Västmanland has a higher proportion of treated patients but from a national perspective the burden of untreated patients is also higher in the region of Västmanland. Therefore, if the DA is low, any potential

For peer review only - http://bmjopen.bmj.com/site/about/guidelines.xhtml

intervention to increase statin treatment after AMI needs to be universal (i.e., on the whole country) and directed to all AMI patients no matter the region they belong to.

Similarly, in Table 2 we can estimate that the number of male AMI patients without statins (n = 3,312) was much higher than the number of female AMI patients without statins (n = 2,411) even though women consistently have a lower prevalence and a lower PR of statin dispensation than men. This reflects that while the prevalence of using statins is higher among men, the fact that AMI patients are disproportionately male means that majority of non-statin users are nevertheless also men.

One further strength of this study is the quality of the data since all information is based on national registers where the information is recorded in a highly standardized way for both ICD and ATC codes. The validity of the AMI diagnosis as recorded in the NPR is high, [39] and the sociodemographic information is recorded by Statistics Sweden with a longstanding experience in the field [https://www.scb.se/en/About-us/main-activity/quality-work/]. Moreover, we can directly compare our results to those presented by the NBHW since the definition of the quality indicator is the same.

One limitation of our study is that we excluded the 5,424 individuals who died, emigrated, or had missing information on country of birth which corresponds to approximately 13% of the final study population, and it cannot be ruled out that this exclusion introduces a selection bias. However, we do not think that this limitation alters our conclusions as the

For peer review only - http://bmjopen.bmj.com/site/about/guidelines.xhtml

selection criteria are in concordance with the those used by the Swedish authorities. Another limitation in this study could be the use of household income as a proxy for socioeconomic position, which does not capture other financial assets, nor necessarily higher education.

The definition of country of birth within this study could be elaborated with more refined categories, other than native and immigrant. Immigrant is a broad concept which includes, among other factors, cultural, economic, and educational heritages from another countries. However, more categories of country of birth would result in a larger number of multicategorical strata with a smaller number of within strata observations. Similarly, there is a possible underestimation of the impact of age since we only use two categories in our analyses. We chose two categories to keep the analysis as simple as possible and also because in the case of the quality indicator we analyze we think it is sufficient to include the age of retirement (i.e., 65 years for most patients at the time of the study) as the cut-off. It is know that this division is relevant for understanding socioeconomic differences in health care utilization [40]. However, for other quality indicators it may be better to provide more age classes.

We use the AUC as a measure of discriminatory accuracy and in some situations when e.g., false negatives are more likely to occur than false positives because of information bias, assuming the same weight for both false negatives and positive might give misleading AUC values. However, this is not a problem in biomedical applications in

For peer review only - http://bmjopen.bmj.com/site/about/guidelines.xhtml

general and in our study in particular as positive or negative events are easy to identify (a patient uses statins or not) and the information is, less probably, differentially biased. The AUC value may also depend on the size of the group being compared. For instance, a stratum may have a high predicted probability of using statins so all individuals in the stratum are classified as “positive” but if the stratum is very large the number of individuals in the stratum who do not use statins (i.e., false positive) may be large and the AUC reduced. This could be prevented by weighting the strata by the inverse of the number of individuals or using other measures of discriminatory accuracy like those based on components of variance.[9] However, in our case the unweighted results are relevant *per se*. A low unweighted AUC would suggest that any potential intervention to increase use of statins should not be targeted to specific strata with a low predicted probability of statin use but be universal and directed to all the strata since there are many patients in the strata with high predicted probabilities who do not use statins. For instance, in our study 37% (2,146/5,723) of the patients who did not use statins reside in the 10 counties with highest predicted probability of using statins, and this value was 58% (3,312/5,723) for the 12 sociodemographic strata with highest predicted probability of using statins.

Implications and Conclusions

Dispensations of statins after AMI in Sweden are, according to the national quality indicator, closely but not fully met in relation to the benchmark value of 90%. However,

For peer review only - http://bmjopen.bmj.com/site/about/guidelines.xhtml

neither regional nor sociodemographic differences in group averages seem to be major determinants of dispensation of statins. Therefore, any intervention aiming to reach the benchmark value should be universal. Sociodemographic differences nevertheless appeared more relevant than geographic differences. However, our results suggest that in spite the lower prevalence of statin prescription in some groups (e.g., women, especially to those with immigrant background and older than 65 years), the exclusive targeting of interventions to those groups would be unsuitable as many AMI patients lacking statin prescription are in the groups with higher prevalence of statin prescription. In other words, we cannot base decisions on stratum prevalence alone, we also need to consider the size of the strata.

In conclusion, as we illustrate in our study, AIHDA is not complicated to implement, and it provides more nuanced information than traditional comparisons of quality of healthcare performance based only on group averages. We therefore propose the application of the AIHDA framework for the quantitative assessment of healthcare quality by geographic and sociodemographic comparisons of quality indicators.

Footnotes

- **Contributors** JM had the initiative of the study and acquired the data. JM and JÖ wrote the original manuscript. RP-V and JÖ performed the analyses in coordination with JM, KK and GL. All the authors have contributed to the design of the study, JM and GL developed the methodology and directed the analyses. All

For peer review only - http://bmjopen.bmj.com/site/about/guidelines.xhtml

authors have contributed to the interpretation of the results and drafting of the manuscript. All authors have revised the last version of the manuscript.

- **Funding** The Swedish Research Council (Vetenskapsrådet) supported this work through the project 'Multilevel Analyses of Individual Heterogeneity: innovative concepts and methodological approaches in Public Health and Social Epidemiology': (#2017-01321, PI: Juan Merlo).
- **Competing interests** None declared.
- **Ethics approval** Regional ethics committee Lund: ID: 2014/856. Data safety committees from the National board of health and welfare: ID 9542/2015. Statistics Sweden: ID 231424/878144-5.
- **Patient consent for publication** Not required.
- **Provenance and peer review** Not commissioned, externally peer reviewed.
- **Data availability statement** Data are available upon reasonable request. Original data are available from the Swedish National Board of Health and Welfare and Statistics, after approval of a research project by an Ethical Committee and by the data safety committees of Swedish Authorities.

For peer review only - http://bmjopen.bmj.com/site/about/guidelines.xhtml

References

1. Socialstyrelsen. God vård – om ledningssystem för kvalitet och patientsäkerhet i hälso- och sjukvården. In: Welfare SBoHa, ed., 2006:ISBN: 91-85482-05-6.
2. Heurgren M, Åberg A, Köster M, et al. Performance indicators in Swedish Health Care. *BMC Health Serv Res* 2007;7(A10) doi: ttps://doi.org/10.1186/1472-6963-7-S1-A10
3. Öppna jämförelser av hälso- och sjukvårdens kvalitet och effektivitet Jämförelser mellan landsting . *Sveriges Kommuner och Landsting wwwsklse/publikationer ISBN 978-91-7164-485-5* 2009
4. Sorman H. [Open comparisons of health care: "One of the most important contributions to modernization of health care"]. *Lakartidningen* 2010;107(32-33):1828-9. [published Online First: 2010/09/29]
5. Khalaf K, Axelsson Fisk S, Ekberg-Jansson A, et al. Geographical and sociodemographic differences in discontinuation of medication for Chronic Obstructive Pulmonary Disease - A Cross-Classified Multilevel Analysis of Individual Heterogeneity and Discriminatory Accuracy (MAIHDA). *Clin Epidemiol* 2020;12:783-96. doi: 10.2147/CLEP.S247368 [published Online First: 2020/08/09]
6. Merlo J, Mulinari S, Wemrell M, et al. The tyranny of the averages and the indiscriminate use of risk factors in Public Health: The case of coronary heart disease. *SSM - Population Health* 2017;3:684-98. doi: https://doi.org/10.1016/j.ssmph.2017.08.005 [published Online First: 2017.08.005]
7. Merlo J. Invited commentary: multilevel analysis of individual heterogeneity-a fundamental critique of the current probabilistic risk factor epidemiology. *Am J Epidemiol* 2014;180(2):208-12; discussion 13-4. doi: 10.1093/aje/kwu108
8. Merlo J. Multilevel analytical approaches in social epidemiology: measures of health variation compared with traditional measures of association. *J Epidemiol Community Health* 2003;57(8):550-2. doi: 10.1136/jech.57.8.550 [published Online First: 2003/07/29]
9. Merlo J, Wagner P, Leckie G. A simple multilevel approach for analysing geographical inequalities in public health reports: The case of municipality differences in obesity. *Health Place* 2019;58:102145. doi: 10.1016/j.healthplace.2019.102145 [published Online First: 2019/06/14]
10. Merlo J. Multilevel analysis of individual heterogeneity and discriminatory accuracy (MAIHDA) within an intersectional framework. *Soc Sci Med* 2018;203:74-80. doi: 10.1016/j.socscimed.2017.12.026 [published Online First: 2018/01/07]
11. Merlo J. Flernivåanalyser behövs i öppna jämförelser inom hälso- och sjukvård. *Läkartidningen 2019;116:FTEL* 2019
12. Evans CR, Erickson N. Intersectionality and depression in adolescence and early adulthood: A MAIHDA analysis of the national longitudinal study of adolescent to adult health, 1995-2008. *Soc Sci Med* 2019;220:1-11. doi: 10.1016/j.socscimed.2018.10.019
13. Mulinari S, Bredstrom A, Merlo J. Questioning the discriminatory accuracy of broad migrant categories in public health: self-rated health in Sweden. *Eur J Public Health* 2015;25(6):911-7. doi: 10.1093/eurpub/ckv099
14. Ljungman H, Wemrell M, Khalaf K, et al. Antidepressant use in Sweden: an intersectional multilevel analysis of individual heterogeneity and discriminatory accuracy (MAIHDA). *Scand J Public Health* 2021:1403494821993723. doi: 10.1177/1403494821993723 [published Online First: 2021/02/24]
15. Axelsson Fisk S, Lindstrom M, Perez-Vicente R, et al. Understanding the complexity of socioeconomic disparities in smoking prevalence in Sweden: a cross-sectional study applying intersectionality theory. *BMJ Open* 2021;11(2):e042323. doi: 10.1136/bmjopen-2020-042323 [published Online First: 2021/02/13]
16. Axelsson Fisk S, Mulinari S, Wemrell M, et al. Chronic Obstructive Pulmonary Disease in Sweden: An intersectional multilevel analysis of individual heterogeneity and discriminatory accuracy. *SSM Popul Health* 2018;4:334-46. doi: 10.1016/j.ssmph.2018.03.005

For peer review only - http://bmjopen.bmj.com/site/about/guidelines.xhtml

17. Wemrell M, Bennet L, Merlo J. Understanding the complexity of socioeconomic disparities in type 2 diabetes risk: a study of 4.3 million people in Sweden. *BMJ Open Diabetes Res Care* 2019;7(1):e000749. doi: 10.1136/bmjdrc-2019-000749 [published Online First: 2019/12/05]
18. Hernandez-Yumar A, Wemrell M, Abasolo Alesson I, et al. Socioeconomic differences in body mass index in Spain: An intersectional multilevel analysis of individual heterogeneity and discriminatory accuracy. *PLoS One* 2018;13(12):e0208624. doi: 10.1371/journal.pone.0208624
19. Persmark A, Wemrell M, Zettermark S, et al. Precision public health: Mapping socioeconomic disparities in opioid dispensations at Swedish pharmacies by Multilevel Analysis of Individual Heterogeneity and Discriminatory Accuracy (MAIHDA). *PLoS One* 2019;14(8):e0220322. doi: 10.1371/journal.pone.0220322 [published Online First: 2019/08/28]
20. Rodriguez-Lopez M, Merlo J, Perez-Vicente R, et al. Cross-classified Multilevel Analysis of Individual Heterogeneity and Discriminatory Accuracy (MAIHDA) to evaluate hospital performance: the case of hospital differences in patient survival after acute myocardial infarction. *BMJ Open* 2020;10(10):e036130. doi: 10.1136/bmjopen-2019-036130 [published Online First: 2020/10/26]
21. Ghith N, Frolich A, Merlo J. The role of the clinical departments for understanding patient heterogeneity in one-year mortality after a diagnosis of heart failure: A multilevel analysis of individual heterogeneity for profiling provider outcomes. *PLoS One* 2017;12(12):e0189050. doi: 10.1371/journal.pone.0189050
22. Wemrell M, Karlsson N, Perez Vicente R, et al. An intersectional analysis providing more precise information on inequities in self-rated health. *Int J Equity Health* 2021;20(1):54. doi: 10.1186/s12939-020-01368-0 [published Online First: 2021/02/05]
23. Ljungman H, Wemrell M, Khalaf K, et al. Antidepressant use in Sweden: an intersectional multilevel analysis of individual heterogeneity and discriminatory accuracy (MAIHDA). *Scand J Public Health* 2022;50(3):395-403. doi: 10.1177/1403494821993723 [published Online First: 2021/02/24]
24. Zettermark S, Khalaf K, Perez-Vicente R, et al. Population heterogeneity in associations between hormonal contraception and antidepressant use in Sweden: a prospective cohort study applying intersectional multilevel analysis of individual heterogeneity and discriminatory accuracy (MAIHDA). *BMJ Open* 2021;11(10):e049553. doi: 10.1136/bmjopen-2021-049553 [published Online First: 2021/10/03]
25. Evans CR, Leckie G, Merlo J. Multilevel versus single-level regression for the analysis of multilevel information: The case of quantitative intersectional analysis. *Soc Sci Med* 2020;245:112499. doi: 10.1016/j.socscimed.2019.112499 [published Online First: 2019/09/23]
26. Socialstyrelsen. Nationella riktlinjer för hjärtsjukvård Stöd för styrning och ledning. In: 2018-6-28| A, ed. Stockholm, Sweden, 2018.
27. Ruiz-Cantero MT, Blasco-Blasco M, Chilet-Rosell E, et al. Gender bias in therapeutic effort: from research to health care. *Farm Hosp* 2020;44(3):109-13. doi: 10.7399/fh.11394 [published Online First: 2020/05/27]
28. Chang AY, Abou-Arraj NE, Rodriguez F. Interventions to Reduce Ethnic and Racial Disparities in Dyslipidemia Management. *Current treatment options in cardiovascular medicine* 2019;21(5):24. doi: 10.1007/s11936-019-0725-4 [published Online First: 2019/05/09]
29. Wylie LE, Waterbrook AL, Dalen JE. Are Statins Indicated in Senior Citizens? A Review of Statin Therapy in the Elderly. *Am J Med* 2021 doi: 10.1016/j.amjmed.2021.11.009 [published Online First: 2021/12/03]
30. Hyun KK, Brieger D, Woodward M, et al. The effect of socioeconomic disadvantage on prescription of guideline-recommended medications for patients with acute coronary syndrome: systematic review and meta-analysis. *Int J Equity Health* 2017;16(1):162. doi: 10.1186/s12939-017-0658-z [published Online First: 2017/09/02]
31. Barros AJ, Hirakata VN. Alternatives for logistic regression in cross-sectional studies: an empirical comparison of models that directly estimate the prevalence ratio. *BMC Med Res Methodol* 2003;3:21. doi: 10.1186/1471-2288-3-21

For peer review only - http://bmjopen.bmj.com/site/about/guidelines.xhtml

32. Hosmer DW, Lemeshow S. Applied logistic regression. 2nd ed. New York: Wiley 2000.
33. Pepe M. S., Janes H., Longton G., et al. Limitations of the odds ratio in gauging the performance of a diagnostic, prognostic, or screening marker. *Am J Epidemiol* 2004;159(9):882-90. [published Online First: 2004/04/24]
34. Marmot M, Bell R. Fair society, healthy lives. *Public Health* 2012;126 Suppl 1:S4-S10. doi: 10.1016/j.puhe.2012.05.014 [published Online First: 2012/07/13]
35. Carey G, Crammond B, De Leeuw E. Towards health equity: a framework for the application of proportionate universalism. *Int J Equity Health* 2015;14:81. doi: 10.1186/s12939-015-0207-6
36. Merlo J, Wagner P, Ghith N, et al. An Original Stepwise Multilevel Logistic Regression Analysis of Discriminatory Accuracy: The Case of Neighbourhoods and Health. *PLoS One* 2016;11(4):e0153778. https://doi.org/10.1371/journal.pone.78. doi: https://doi.org/10.1371/journal.pone.0153778
37. Moons KG, de Groot JA, Bouwmeester W, et al. Critical appraisal and data extraction for systematic reviews of prediction modelling studies: the CHARMS checklist. *PLoS Med* 2014;11(10):e1001744. doi: 10.1371/journal.pmed.1001744
38. Ginsburg GS, Phillips KA. Precision Medicine: From Science To Value. *Health Aff (Millwood)* 2018;37(5):694-701. doi: 10.1377/hlthaff.2017.1624 [published Online First: 2018/05/08]
39. Ludvigsson JF, Andersson E, Ekbom A, et al. External review and validation of the Swedish national inpatient register. *BMC Public Health* 2011;11:450. doi: 10.1186/1471-2458-11-450
40. Merlo J, Gerdtham UG, Lynch J, et al. Social inequalities in health – do they diminish with age? Revisiting the question in Sweden 1999. *Int J Equity Health* 2003;2(1):2.

For peer review only

For peer review only - http://bmjopen.bmj.com/site/about/guidelines.xhtml

Figure 1: Study population flow-chart

Figure 2: Crude prevalence (P) and 95% confidence intervals (CI) for use of statins 12 -18 months after hospital discharge with an acute myocardial infarction (AMI) diagnosis in patients aged 40-80 years in the period 2011-2013, across the 21 administrative regions of Sweden.

Figure 3: Crude prevalence (P) and 95% confidence intervals (CI) for use of statins 12 -18 months after hospital discharge with an acute myocardial infarction (AMI) diagnosis in patients aged 40-80 years in the period 2011-2013, across the 24 sociodemographic groups.

For peer review only

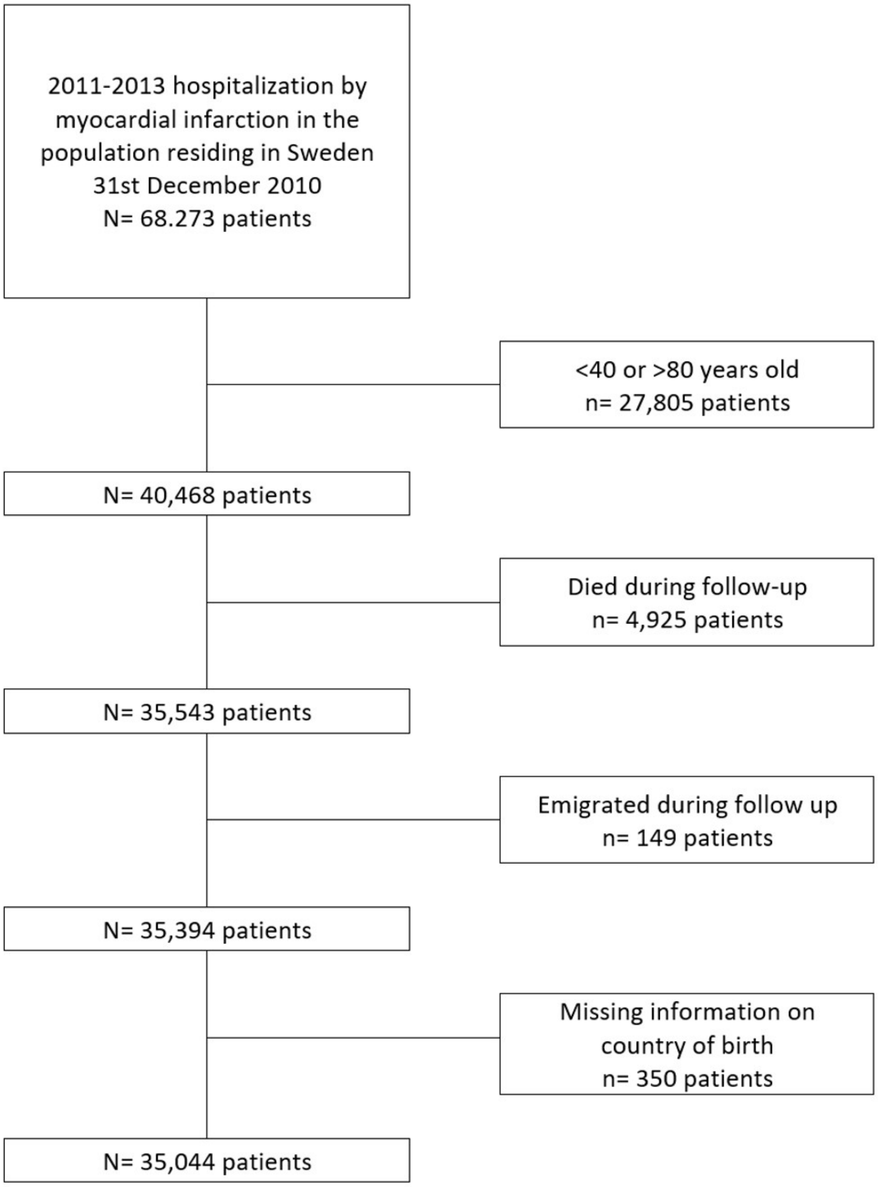

Figure 1: Study population flow-chart

261x348mm (96 x 96 DPI)

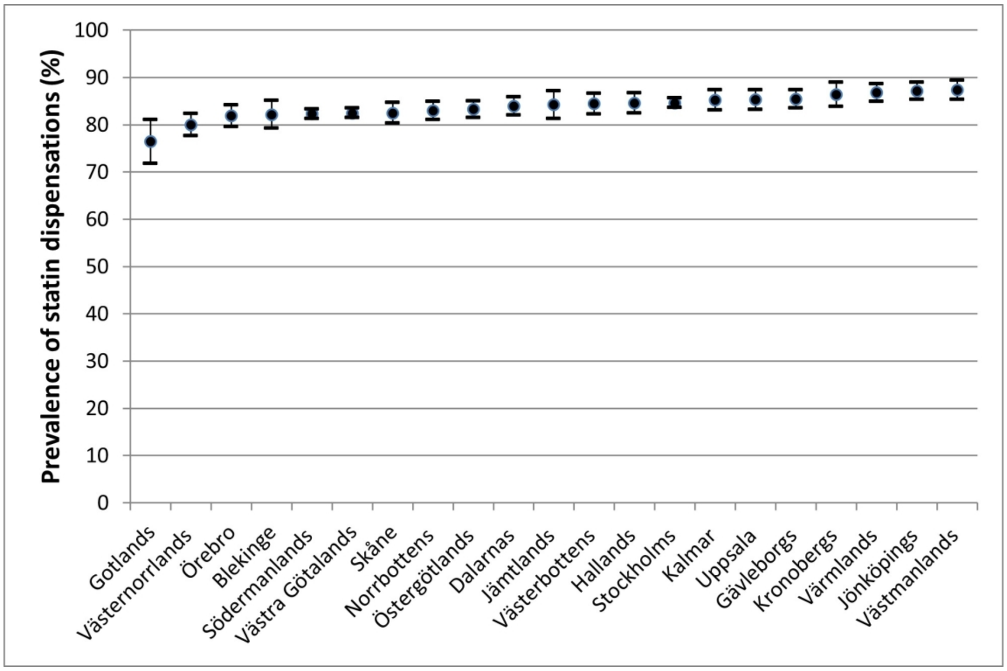

Figure 2: Crude prevalence (P) and 95% confidence intervals (CI) for use of statins 12 -18 months after hospital discharge with an acute myocardial infarction (AMI) diagnosis in patients aged 40-80 years in the period 2011-2013, across the 21 administrative regions of Sweden.

152x101mm (330 x 330 DPI)

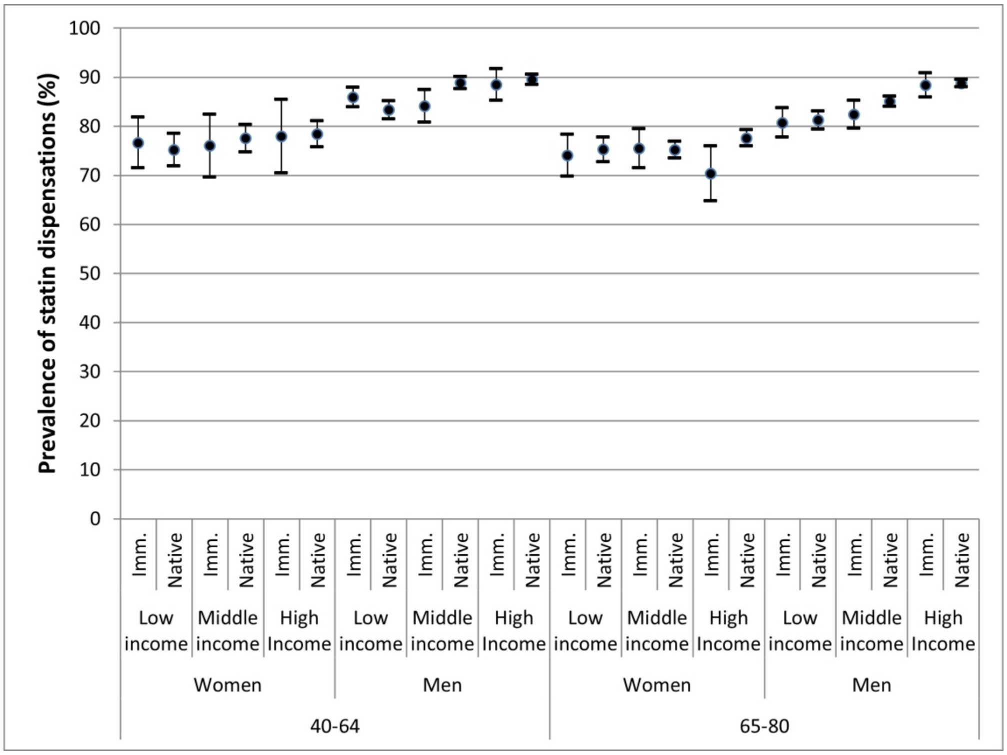

Figure 3: Crude prevalence (P) and 95% confidence intervals (CI) for use of statins 12 -18 months after hospital discharge with an acute myocardial infarction (AMI) diagnosis in patients aged 40-80 years in the period 2011-2013, across the 24 sociodemographic groups.

165x123mm (330 x 330 DPI)

SUPLEMENTARY MATERIAL (S)

S1: Supplementary analyses

SM 1. Prevalence Ratios (PR) and (95% confidence intervals) for use of statins 12-18 months after hospital discharge with an acute myocardial infarction (AMI) diagnosis in patients aged, 40-80 years old from 24 sociodemographic strata in the period 2011-2013

	Age	Sex	Income	Country of birth	All	Multicategorical *
Age						
40-64	1.03 (1.02-1.04)				1.02 (1.01-1.04)	
65-80	Reference					
Sex						
Women		Reference				
Men		1.14 (1.12-1.15)			1.13 (1.12-1.14)	
Income						
Low			Reference			
Middle			1.03 (1.01-1.04)		1.03 (1.01-1.04)	
High			1.07 (1.06-1.09)		1.06 (1.05-1.08)	
Country of birth						
Immigrant				Reference		
Native				1.03 (1.01-1.04)	1.01 (1.00-1.03)	
AUC	0.512	0.579	0.546	0.511	0.608	0.609

*Model 1B in table 2. AUC: area under the ROC curve

For peer review only

S2: Stata codes

Main analysis

```
. use cohort_selected.dta, clear

. generate time = 1
. stset time, failure(statins) id(lpnr)

* Model 1a
. stcox ib11.region, vce(robust)
. predict p1a

* Model 1b
. stcox ib12.inter, vce(robust)
. predict p1b

* Model 2
. stcox ib11.region ib12.inter, vce(robust)
. predict p2

. roccomp statins p1a p1b p2
```

Prevalences

```
. use cohort_selected.dta, clear
. by region, sort: egen cases = sum(statins)
. by region: gen total = _N
. keep cases total region
. duplicates drop

. use cohort_selected.dta, clear
. by inter, sort: egen cases = sum(statins)
. by inter: generate total = _N
. keep cases total inter
. duplicates drop

. use cohort_selected.dta, clear
. egen cases = sum(statins)
. display _N
. tabulate cases
```

Supplementary analysis

```
. use cohort_selected.dta, clear
```


For peer review only - http://bmjopen.bmj.com/site/about/guidelines.xhtml

```
. generate time = 1
. stset time, failure(statins) id(lpnr)

* Model 1b_age
. stcox ib1.age_cat, vce(robust)
. predict p2a

* Model 1b_sex
. stcox i.male, vce(robust)
. predict p2b

* Model 1b_income
. stcox i.cum, vce(robust)
. predict p2c

* Model 1b_country
. stcox i.Sw, vce(robust)
. predict p2d

* Model 1b_all
. stcox ib1.age_cat i.male i.cum i.Sw, vce(robust)
. predict p2e

. roccomp statins p2*
```


For peer review only - http://bmjopen.bmj.com/site/about/guidelines.xhtml

STROBE Statement—Checklist of items that should be included in reports of ***cross-sectional studies***

	Item No	Recommendation	Page No
**Title and abstract**	1	(*a*) Indicate the study's design with a commonly used term in the title or the abstract	1-2
		(*b*) Provide in the abstract an informative and balanced summary of what was done and what was found	2
**Introduction**			
Background/rationale	2	Explain the scientific background and rationale for the investigation being reported	4-7
Objectives	3	State specific objectives, including any prespecified hypotheses	4-6
**Methods**			
Study design	4	Present key elements of study design early in the paper	7-8
Setting	5	Describe the setting, locations, and relevant dates, including periods of recruitment, exposure, follow-up, and data collection	4, 7-8
Participants	6	(*a*) Give the eligibility criteria, and the sources and methods of selection of participants	7-8
Variables	7	Clearly define all outcomes, exposures, predictors, potential confounders, and effect modifiers. Give diagnostic criteria, if applicable	8-9
Data sources/ measurement	8*	For each variable of interest, give sources of data and details of methods of assessment (measurement). Describe comparability of assessment methods if there is more than one group	7-9
Bias	9	Describe any efforts to address potential sources of bias	7-9
Study size	10	Explain how the study size was arrived at	7-8
Quantitative variables	11	Explain how quantitative variables were handled in the analyses. If applicable, describe which groupings were chosen and why	7-9
Statistical methods	12	(*a*) Describe all statistical methods, including those used to control for confounding	9-14
		(*b*) Describe any methods used to examine subgroups and interactions	9-14
		(*c*) Explain how missing data were addressed	7-8
		(*d*) If applicable, describe analytical methods taking account of sampling strategy	
		(*e*) Describe any sensitivity analyses	
**Results**			
Participants	13*	(a) Report numbers of individuals at each stage of study—eg numbers potentially eligible, examined for eligibility, confirmed eligible, included in the study, completing follow-up, and analysed	7-8
		(b) Give reasons for non-participation at each stage	7-8, 28
		(c) Consider use of a flow diagram	28
Descriptive data	14*	(a) Give characteristics of study participants (eg demographic, clinical, social) and information on exposures and potential confounders	15, 17, 28
		(b) Indicate number of participants with missing data for each variable of interest	28
Outcome data	15*	Report numbers of outcome events or summary measures	15, 17

For peer review only

For peer review only - http://bmjopen.bmj.com/site/about/guidelines.xhtml

Main results	16	(*a*) Give unadjusted estimates and, if applicable, confounder-adjusted estimates and their precision (eg, 95% confidence interval). Make clear which confounders were adjusted for and why they were included	15, 17
		(*b*) Report category boundaries when continuous variables were categorized	8-9, 15, 17
		(*c*) If relevant, consider translating estimates of relative risk into absolute risk for a meaningful time period	
Other analyses	17	Report other analyses done—eg analyses of subgroups and interactions, and sensitivity analyses	
**Discussion**			
Key results	18	Summarise key results with reference to study objectives	14-18
Limitations	19	Discuss limitations of the study, taking into account sources of potential bias or imprecision. Discuss both direction and magnitude of any potential bias	20-23
Interpretation	20	Give a cautious overall interpretation of results considering objectives, limitations, multiplicity of analyses, results from similar studies, and other relevant evidence	20-23
Generalisability	21	Discuss the generalisability (external validity) of the study results	20-23
**Other information**			
Funding	22	Give the source of funding and the role of the funders for the present study and, if applicable, for the original study on which the present article is based	24

*Give information separately for exposed and unexposed groups.

Note: An Explanation and Elaboration article discusses each checklist item and gives methodological background and published examples of transparent reporting. The STROBE checklist is best used in conjunction with this article (freely available on the Web sites of PLoS Medicine at http://www.plosmedicine.org/, Annals of Internal Medicine at http://www.annals.org/, and Epidemiology at http://www.epidem.com/). Information on the STROBE Initiative is available at www.strobe-statement.org.

For peer review only

For peer review only - http://bmjopen.bmj.com/site/about/guidelines.xhtml

BMJ Open

Geographic and sociodemographic differences in statin dispensation after acute myocardial infarction in Sweden – A register based prospective cohort study applying analysis of individual heterogeneity and discriminatory accuracy (AIHDA) for basic comparisons of healthcare quality.

Journal:	*BMJ Open*
Manuscript ID	bmjopen-2022-063117.R3
Article Type:	Original research
Date Submitted by the Author:	04-Jul-2023
Complete List of Authors:	Merlo, Juan; Lunds University Faculty of Medicine, Social Epidemiology<br>Öberg, Johan; Lund University Clinical Sciences, Unit for Social Epidemiology<br>Khalaf, Kani; Lund University, Department of Clinical Sciences<br>Perez-Vicente, Raquel; Lund University, Faculty of Medicine, Social Epidemiology<br>Leckie, George; University of Bristol, Multilevel Modelling
**Primary Subject Heading**:	Epidemiology
Secondary Subject Heading:	Epidemiology, Health services research
Keywords:	EPIDEMIOLOGY, Myocardial infarction < CARDIOLOGY, Quality in health care < HEALTH SERVICES ADMINISTRATION & MANAGEMENT, CLINICAL PHARMACOLOGY

SCHOLARONE™
Manuscripts

I, the Submitting Author has the right to grant and does grant on behalf of all authors of the Work (as defined in the below author licence), an exclusive licence and/or a non-exclusive licence for contributions from authors who are: i) UK Crown employees; ii) where BMJ has agreed a CC-BY licence shall apply, and/or iii) in accordance with the terms applicable for US Federal Government officers or employees acting as part of their official duties; on a worldwide, perpetual, irrevocable, royalty-free basis to BMJ Publishing Group Ltd ("BMJ") its licensees and where the relevant Journal is co-owned by BMJ to the co-owners of the Journal, to publish the Work in this journal and any other BMJ products and to exploit all rights, as set out in our licence.

The Submitting Author accepts and understands that any supply made under these terms is made by BMJ to the Submitting Author unless you are acting as an employee on behalf of your employer or a postgraduate student of an affiliated institution which is paying any applicable article publishing charge ("APC") for Open Access articles. Where the Submitting Author wishes to make the Work available on an Open Access basis (and intends to pay the relevant APC), the terms of reuse of such Open Access shall be governed by a Creative Commons licence – details of these licences and which Creative Commons licence will apply to this Work are set out in our licence referred to above.

Other than as permitted in any relevant BMJ Author's Self Archiving Policies, I confirm this Work has not been accepted for publication elsewhere, is not being considered for publication elsewhere and does not duplicate material already published. I confirm all authors consent to publication of this Work and authorise the granting of this licence.

For peer review only

Geographic and sociodemographic differences in statin dispensation after acute myocardial infarction in Sweden – A register based prospective cohort study applying analysis of individual heterogeneity and discriminatory accuracy (AIHDA) for basic comparisons of healthcare quality.

Juan Merlo[1, 2] ORCID: 0000-0001-8379-9708

Johan Öberg[1, 3] ORCID: 0000-0002-0701-5155

Kani Khalaf[1, 3] ORCID: 0000-0002-6749-7037

Raquel Perez Vicente[1] ORCID: 0000-0002-6273-1656

George Leckie[4] ORCID: 0000-0003-1486-745X

[1] Unit for Social Epidemiology, Faculty of Medicine, Lund University, Malmö, Sweden

[2] Centre for Primary Health Care Research, Region Skåne, Malmö, Sweden

[3] Department of Health and Medical Care Management, Region Skåne, Malmö, Sweden

[4] Centre for Multilevel Modelling, University of Bristol, UK

Corresponding author: Prof. Juan Merlo juan.merlo@med.lu.se, Skåne University Hospital, CRC, Jan Waldenströms gata 35 building 60, floor 12, SE-205 02 Malmö, Sweden, +46 70 5092265

Keywords (MeSH): Social Epidemiology, Health Care Quality Assessment, Pharmacologies, Health Services Evaluation, Epidemiological Methods

Word count: 5464

Abstract word count: 247

Number of references: 42

For peer review only

Abstract

Background: In Sweden, as in many other countries, official monitoring of healthcare quality is mostly focused on geographical disparities in relation to a desirable benchmark. However, current evaluations could be improved by considering: (i) the intersection of other relevant axes of inequity like age, sex, income, and migration status; and (ii) the existence of individual heterogeneity around averages. Therefore, using an established quality indicator (i.e., dispensation of statins after acute myocardial infarction, AMI), we valuate both geographical and sociodemographic inequalities and illustrate how the analysis of individual heterogeneity and discriminatory accuracy (AIHDA) enhances such evaluations.

Population and methods: We applied AIHDA and calculated the area under the ROC curve (AUC) of regional and sociodemographic differences in the statin dispensations of 35,044 patients from 21 Swedish regions and 24 sociodemographic strata who were discharged from the hospital with an AMI diagnosis between January 2011 and December 2013. Following the Swedish National Board of Health and Welfare, we used a benchmark value of 90%.

Results: Dispensation of stains after AMI in Sweden did not reach the desired target of 90%. Regional differences were absent/very small (AUC = 0.537) while sociodemographic differences were small (AUC = 0.618). Women, especially those with

For peer review only - http://bmjopen.bmj.com/site/about/guidelines.xhtml

immigrant background and older than 65 years, have the lowest proportions of statin dispensations after AMI.

Conclusions: As the AUC statistics are small, interventions trying to achieve the benchmark value should be universal. However, a special emphasis should nevertheless be directed towards women, especially older women with immigrant backgrounds.

For peer review only

For peer review only - http://bmjopen.bmj.com/site/about/guidelines.xhtml

Strengths and limitations of this study:

- The applied Analysis of Individual Heterogeneity and Discriminatory Accuracy (AIHDA) provides an improved mapping and quantification of geographical and sociodemographic inequalities in healthcare quality
- The quality of the data in our study is high as it is based on national registers with valid and **standardized** information on diagnoses, medication use and sociodemographic variables
- We can directly compare our results to those presented by public authorities, such as the Swedish National Boars of Health and Welfare, since the definition of the quality indicator is the same.
- The definition and categorization of the sociodemographic variables is coarse. However, more fine categorizations would excessively increase the number of strata
- The AIHDA approach can be implemented using both traditional regression or more advanced multilevel models which provide several advantages. However, traditional regression is a valid and accessible alternative

For peer review only - http://bmjopen.bmj.com/site/about/guidelines.xhtml

Introduction

In Sweden as in many other countries, policies and laws emanating from patient needs regulate and define standards of healthcare.[1] Good healthcare is defined as safe, accessible, delivered in time, health promoting, and knowledge- and evidence-based. In addition, to be fully efficient and effective, good healthcare must be equitable. That is, on equal terms and according to patients' needs. On this background, The Swedish National Board of Health and Welfare (NBHW) and the Swedish Association of Local Authorities and Regions (SALAR) define quality indicators[2] that can be compared against specific benchmarks. These quality indicators are regularly monitored for quality of performance assessment by means of simplified hospital and regional comparisons online (https://vardenisiffror.se/), and in reports such as *Open comparisons of healthcare performance.*[3] The benefits of monitoring for improving healthcare quality are many.[4] However, current comparisons could be improved for several reasons.

In spite of the current evidence on the existence of healthcare inequities conditioned by sociodemographic factors (e.g., age, sex, income, country of birth), today's official monitoring is mostly focused on geographical disparities, disregarding other relevant axes of possible inequity. Monitoring both geographic and sociodemographic inequalities seem necessary in order to improve the evaluation of quality of healthcare performance.[5]

In addition, current comparisons are often based on the analysis and interpretation of

For peer review only - http://bmjopen.bmj.com/site/about/guidelines.xhtml

differences between group averages, disregarding individual heterogeneity around such averages.[6, 7] This is a fundamental but often ignored question in epidemiology and healthcare evaluation.[6-10] To understand this concept, we strongly suggest the reading of a previous example[9] describing in detail this idea in non-technical terms. When comparing group averages, such as hospital averages in quality outcomes, we also need to quantify the extent of the groups' distributions of individual values (i.e., heterogeneity around averages). The size of this overlap needs be considered when evaluating differences between group averages.[9]

Also, the criteria for quantifying the size of the group differences in official comparisons are not clearly stated. For instance, when can we say that there are large regional differences in a quality indicator? The criterion of "statistical significance" is not sufficient as very small and meaningless differences between group averages may nonetheless prove statistically significant if the sample is large enough. Therefore, measurable differences between group averages may exist together with considerable overlap of individual heterogeneity between groups, which may render the average differences irrelevant.[9, 11] Therefore, group differences should not only be assessed by simple measures of differences between averages but also as the share of the total individual variance that is at the group level. Alternatively, we should quantify the accuracy of the information provided by the groups differences to classify persons according to the outcome.

For peer review only - http://bmjopen.bmj.com/site/about/guidelines.xhtml

The analysis of individual heterogeneity and discriminatory accuracy (AIHDA) we promote in this study, is an innovative analytical strategy that has the potential to address the above criticisms and thereby improve the quantitative evaluation of health care quality. AIHDA is not a new statistical technique but rather a strategy of analysis that stress the importance of focusing on components of individual heterogeneity and discriminatory accuracy and not only on differences between group averages (as it is the traditional approach). AIHDA is proving an increasingly popular approach in public health and health care epidemiology.[5, 9, 10, 12-21] When applying AIHDA, we do not consider differences between group averages and differences between patients as if they are two separate and unrelated phenomena of interest. Rather, we adopt a conceptually multilevel perspective that simultaneously considers both types of differences. From this standpoint, we evaluate sociodemographic and geographical group differences by quantifying the share of the total individual variation in patient outcomes that operates at the group level. In the case of binary patient outcomes (like most quality indicators), we can analogously evaluate the accuracy of the group level for discriminating individuals with the outcome from those without the outcome of interest. Applying AIHDA, we can evaluate the relevance of specific group differences (e.g., regional differences), and even compare the relative relevance of different grouping criteria (e.g., sociodemographic strata versus geographical regions).

AIHDA can be applied using traditional regression[13, 15, 17, 22] or more advanced

For peer review only - http://bmjopen.bmj.com/site/about/guidelines.xhtml

multilevel regression analyses (MLRA) that we denominate (M)AIHDA.[5, 9, 10, 12, 14, 16, 18-20, 23, 24] MLRA provide conceptual and methodological advantages as compared to traditional regression analyses.[25] For instance, conceptually both kinds of analyses consider the groups as contexts but only MLRA explicitly models the groups as a second level (i.e., as a random effect using technical terminology). In MLRA the relative group differences (e.g., prevalence ratios) have the grand mean (i.e., the mean of the groups' means) as reference while in traditional analyses the reference is a specific group (as in our present study). MLRA can better handle groups with few individuals via providing reliability weighted estimations. The interested reader may obtain a deeper understanding on this issue elsewhere.[25-27]

While we primarily recommended the use of multilevel models[25], for simplified routine monitoring and online presentations, traditional regression analysis provides a valid AIHDA alternative. However, independently of the statistical technique, we want to stress that we consider the groups (e.g., regions or socioeconomic strata) as contexts rather than as characteristics attached to the individuals. That is, conceptually we adopt multilevel perspective that partitions the individual differences into within groups (level 1) and between groups (level 2).

The challenge of the present study is double. First, we aim to introduce a strategy of analysis that may discomfort the readers habituated with traditional focus on group mean differences.[7] Second, we aim to present this strategy in a correct but easy way. Our

For peer review only - http://bmjopen.bmj.com/site/about/guidelines.xhtml

intention is to provide a simple alternative for evaluation of inequalities in routine health care. Still the analytical approach and the way we use some concepts are innovative which may need some extra contemplation. We provide suitable references for the readers who will a deeper understanding of both AIHDA [13, 15, 17, 22] and (M)AIHDA.[5, 9, 10, 12, 14, 16, 18-20, 23, 24]

Motivated by the above background, we illustrate how the AIHDA approach can be applied for public monitoring of geographical as well as sociodemographic and differences in statin treatment after hospitalization for acute myocardial infarction (AMI), which is a well-known process indicator specifically adopted by the Swedish NBHW.[28] We analyze both regional[4] and sociodemographic differences as previous research has shown less dispensations in women[29], immigrants[30], elderly people[31] and in patients with low socioeconomic position.[32] For this purpose, we analyze the statins dispensations of 31,027 patients discharged from the hospital with an AMI diagnosis between January 2011 and December 2013.

Population and methods

Databases

Our investigation is based on a record-linkage database of several registers with national coverage, including the Swedish Population Register (TPR) and the Longitudinal Integration Database for Health Insurance and Labour Market Studies (LISA),

For peer review only - http://bmjopen.bmj.com/site/about/guidelines.xhtml

administrated by Statistics Sweden (SCB), as well as the National Patient Register (NPR), the Cause of Death Register, and the Swedish Prescribed Drug Register (SPDR) administrated by the NBHW. The NPR records both outpatient clinic and inpatient discharges from hospital with diagnoses coded according to the International Classification of Diseases, 10th edition (ICD-10). However, it does not cover information on diagnoses in primary health care. The SPDR contains information on all prescription drug dispensations from Swedish pharmacies. To maintain confidentiality, the registers were linked using an arbitrary serial number assigned to each individual by statistics Sweden instead of the Swedish unique personal identification number.

The Regional Ethics Review Board in Southern Sweden and the data safety committees from the NBHW and from Statistics Sweden approved the construction of the database.

Study population

We defined the study population using the same criteria as applied by the Swedish NBHW for evaluating the quality indicator on statins dispensation after hospital discharge for AMI.

The initial population included all 40,468 patients aged 40-80 years, who were residing in Sweden by 31 December 2010, and were discharged from hospital with an *AMI diagnosis* (ICD-10: I21 or I22) between 1 January 2011 and 31 December 2013. We followed each patient between the 13th and 18th month after discharge from the index AMI hospitalization regarding their statin dispensation from the pharmacies. We excluded

For peer review only

4,925 patients who died between discharge from hospital and end of follow-up,149 patients who emigrated, and 350 patients with missing information on country of birth. The final study population included 35,044 patients. See figure 1 for details. The selection criteria are in concordance with those used by the Swedish authorities to define the quality indicator. Therefore, we do not question the validity of indicator, but just present a way of analysing it,

Assessment of variables

We defined the *quality indicator* according to the Swedish authorities as at least 90% of patients aged 40-80 years with a hospital discharge diagnosis of AMI and as having had at least one statin dispensation between the 13th and 18th month period after the hospital discharge.[28] We define *statin use* as any recorded dispensation of statin or a statin-combination drug (ATC codes C10AA or C10BA) in the SPDR during the follow-up period.

We classified the patients into the 21 *administrative regions* of Sweden according to their legal residence address and used Region Skåne as the reference region in the comparisons. Administrative regions in Sweden form autonomous political units and act as principals for healthcare.

We categorized *age* into two groups: 40-64 years and 65-80. *Sex* was defined as male or female according to the registered information. We categorized patients according to their *country of birth* into native (i.e., born in Sweden) or not (i.e., immigrant).

For peer review only - http://bmjopen.bmj.com/site/about/guidelines.xhtml

We obtained information on *individualised disposable family income* for the years 2000, 2005 and 2010 to compute a cumulative income measure that considers the size of the household and the consumption weight of the individuals according to Statistics Sweden. For each of the three years, income levels were categorised into 25 quantiles using the complete Swedish population. These groups from the respective three years were summed up, so that each individual received a value between 3 (always in the lowest income group) and 75 (always in the highest income group). We categorised this cumulative income measure into three groups by tertiles (low, medium, or high income). Individuals with missing values on income during 2000 or 2005 were assigned the tertile values for the year 2010. No individuals had missing income data for 2010. By calculating a cumulative income measure, we obtain a more stable measurement of socioeconomic position.

Finally, we constructed a *sociodemographic multicategorical variable* consisting of all possible combinations of the categories of the four sociodemographic variables age, sex, income, and immigration status. This resulted in 24 strata.

Patient and public involvement

Because of the nature of our study, patient and public involvement is necessary. We will communicate the results of our study to politicians and healthcare professionals, who represent the public's interest.

For peer review only - http://bmjopen.bmj.com/site/about/guidelines.xhtml

Statistical and epidemiological analysis

To examine the association between geographical and sociodemographic variables and the use of statins, we calculated the crude percentage of patients who were dispensed statins in each region and in each multicategorical sociodemographic strata.

Next, we constructed three regression models following the AIHDA approach. Since the prevalence of the outcome was not extreme, we measured the associations between use of statins and the explanatory variables by prevalence ratios (PRs) and their 95% confidence intervals (CI) rather than by odds ratios. For this purpose, we applied Cox proportional hazards regression models with a constant follow-up time equal to 1 and report heteroskedasticity robust confidence intervals [33] using the *vce(robust)* option in Stata (see supplementary information S1).

Model 1A estimates geographical inequalities by including only the geographical variable with the 21 regions, specifying Region Skåne as reference in the comparisons.

Model 1B estimates sociodemographic inequalities by including the sociodemographic multicategorical variable only, specifying the strata of native 40-64 years old men with high income as the reference category. The focus of our study was the sociodemographic multicategorical variable, but for informative reasons we performed a

For peer review only

For peer review only - http://bmjopen.bmj.com/site/about/guidelines.xhtml

series of intermediate models one variable in turn from the set of variables used to construct the multicategorical variable (see supplementary information S2).

Model 2 enters the geographical variable and the sociodemographic multicategorical variable simultaneously. This model estimates the geographical and sociodemographic differences adjusted for each other.

In order to evaluate and compare the relevance of the sociodemographic and geographical information for understanding use of statins after AMI, we estimated the Discriminatory accuracy (DA) for each model by calculating the area under the receiver operating characteristic curve (AUC) with 95% confidence intervals (CIs). The AUC is obtained by plotting the true-positive fraction against the false-positive fraction for different thresholds of the predicted probability of statin use. The value of the AUC ranges from .5 to 1, with .5 indicating the model has no predictive accuracy and 1 representing perfect discrimination. Using the criteria proposed by Hosmer and Lemeshow [34], we classified DA as absent or very small (.5 ≤ AUC ≤ .6), small (.6 < AUC ≤ .7), large (.7 < AUC ≤ 0.8), or very large (AUC > .8). As explained in previous publications [9, 11], measuring geographical and sociodemographic differences to evaluate healthcare quality is analogous to performing a diagnostic test.[35] No decision-maker in healthcare would want to use such a test without first knowing its discriminatory accuracy (i.e., sensitivity, specificity, AUC). We do not want to unnecessarily treat false positive individuals or incorrectly deny treatment of false negative patients. In our case,

For peer review only - http://bmjopen.bmj.com/site/about/guidelines.xhtml

we want to know the accuracy of public sociodemographic and geographical comparisons for discriminating individuals with a positive result of the quality indicator (i.e., use of statins) from those with a negative result (i.e., no statins).

Strategy for evaluating sociodemographic and geographical differences

Applying the AIHDA framework means that we focus on both the group averages (that is, the region and sociodemographic strata averages) and the individual heterogeneity around these averages (that is, the AUC value, which informs on the overlap in individual heterogeneity across groups).

This framework includes four steps to achieve a complete analysis of inequalities. However, the approach may be modified for different research questions.[9]

In the *first step (i)* we identify the quality indicator and the target or benchmark average value as we described above. In the *second step (ii)* we estimate and visualize the groups' differences using appropriate plots (e.g., caterpillar plots) and in the *third step (iii)* we quantify the size of these differences. That is, for a correct interpretation of the plots, the size of the groups' differences needs to be evaluated using a measure of discriminatory accuracy like the AUC. In traditional comparisons of group averages, the criteria for quantifying the size of the geographical and sociodemographic differences are not clearly stated. However, in the AIHDA framework we evaluate geographical and sociodemographic differences by quantifying the share of the total

For peer review only - http://bmjopen.bmj.com/site/about/guidelines.xhtml

variation in individual outcomes that operates at the group level (i.e., regions, sociodemographic strata). In the case of binary individual health outcomes, we can use the AUC as it provides information expressed in terms of discriminatory accuracy. Finally, in the *fourth step (iv)* we proceed to the interpretation of the results. Following the study referred earlier[9], we present a practical framework for evaluating geographical and sociodemographic differences. For this purpose, we need at least two types of information.

First, we need to know whether the average quality indicator (i.e., percentage of patients on statins after AMI) has reached the predetermined target value fully (i.e., ≥ 90% of all AMI patients are on statins), closely (i.e., 80-89%), or insufficiently (i.e., < 80%). The criteria we use to qualify the prevalence value as “fully” is based on the definition given by Swedish NBHW. However, the qualifications "closely" and “insufficiently” are ours and therefore subjective. More generally, the thresholds defining these categories will differ across quality indicators.

Second, we need information on the *size of the group differences* as assessed by the AUC, which expresses the accuracy of the differences between group averages for discriminating patients with the outcome from those without the outcome. These two types of information need to be combined for the evaluation. Box 1 illustrates these ideas by means of a simple framework with 12 scenarios that can be used to orient the interpretation of an analysis.

For peer review only - http://bmjopen.bmj.com/site/about/guidelines.xhtml

For peer review only

For peer review only - http://bmjopen.bmj.com/site/about/guidelines.xhtml

Box 1. Framework for evaluating geographical and sociodemographic differences in a specific quality indicator. For this purpose, we need information about the *target indicator value*. That is, whether the average quality indicator (i.e., percentage of patients on statins after AMI) has "insufficiently", "closely" or "fully" reached a predetermined target *benchmark* level. We also need information on the area under receiver operating characteristics curve (AUC), which expresses the accuracy of the differences between group averages for discriminating patients with the outcome from those without the outcome. Combining this information, we obtain 12 different scenarios useful for the evaluation.

		**Target indicator value reached**		
**Size of the group differences**		Insufficiently	Closely	Fully
Absent/very small	.5 ≤ AUC ≤ .6	A	B	C
Small	.6 ≤ AUC < .7	D	E	F
Large	.7 ≤ AUC < .8	G	H	I
Very large	.8 ≤ AUC	J	K	L

In the ideal scenario (scenario C) the desired target level has been fully reached in the population of patients, and the group differences are absent or very small. The conclusion would be that all groups have been treated appropriately and on equal terms. From this perspective, the quality of the healthcare is high.

In the worst scenario (scenario A) the desired target level has not been achieved in the population of patients, and the groups differences are again absent. The conclusion would be that all groups have been treated on equal terms but inadequately. That is, the quality of healthcare in the whole population is low.

For peer review only - http://bmjopen.bmj.com/site/about/guidelines.xhtml

Observe that in both scenarios A and C interventions targeted to specific groups are not justifiable. Rather any intervention should be universal (i.e., directed to the whole country). In scenario C, the reason for the intervention would be to maintain the desirable value of the quality indicator in all groups. In the scenario A, the reason would be to reach this desirable level in all the groups.

The interpretation of the scenarios in the lowest corners of the table (J and L) is conditioned by the very large size of the group differences. For example, in scenario L even though the population as a whole has achieved the target level, some groups may not have done so. In contrast, in scenario J, some groups may have achieved the target level even though the population as a whole has not achieved it. In these scenarios (J and L) targeted group interventions are justified.

The framework we propose fits well with the concept of *proportionate universalism* for resource allocation in public health.[36, 37] That is, health care actions must be universal, not targeted, but with a scale and intensity that is proportionate to the level of disadvantage. The AIHDA approach we apply in this study can be used to inform decisions regarding the appropriate scale and intensity for a given sociodemographic or geographic context.

For peer review only - http://bmjopen.bmj.com/site/about/guidelines.xhtml

Results

The overall prevalence of statin dispensations in AMI patients 12-18 months after discharge was 83.7% (29,231/35,044). Therefore, the benchmark value of 90% was *closely* but not fully met on a national level according to the framework in Box 1.

Figure 2 and Table 1 illustrate the prevalence of statin dispensations in each administrative region in Sweden. Västmanland has the highest prevalence of statin use at 87.4% while Gotland has the lowest prevalence at 76.4%. None of the regions reach the benchmark value of 90%. The relative differences when comparing with the region of Skåne (Model 1A) were tiny and Table 1 illustrates that only four regions (i.e., Gotland, Västernorrland, Stockholm and Västra Götaland) presented a slightly but conclusively lower PR than Skåne. No other region showed a conclusively higher PR than Skåne. The PR adjusted for the multicategorical sociodemographic variable (Model 2) were very similar to the unadjusted PR (Model 1A), indicating that the sociodemographic composition of the regions did not confound the geographical differences.

Table 1. Number of patients using statins (n), number of individuals (N), crude prevalence (P) and 95% confidence intervals (CI), unadjusted prevalence ratios (PR), and prevalence ratios adjusted for sociodemographic variables (PRa) for use of statins 12 -18 months after hospital discharge with an acute myocardial infarction (AMI) diagnosis in patients aged 40-80 years from the 21 Swedish geographical administrative regions in the period 2011-2013.

				Model 1A	Model 2
	n	N	P (95% CI) %	PR (95% CI)	PRa (95% CI)

For peer review only - http://bmjopen.bmj.com/site/about/guidelines.xhtml

Sweden	29321	35044	83.67 (83.28-84.06)	–	–
Administrative Regions					
Gotland	246	322	76.40 (71.76-81.04)	0.90 (0.85-0.96)	0.91 (0.85-0.97)
Västernorrland	922	1,153	79.97 (77.65-82.28)	0.95 (0.92-0.98)	0.94 (0.92-0.97)
Örebro	886	1,082	81.89 (79.59-84.18)	0.97 (0.94-1.00)	0.97 (0.94-1.00)
Blekinge	525	639	82.16 (79.19-85.13)	0.97 (0.93-1.01)	0.98 (0.94-1.02)
Stockholm	4,477	5,440	82.30 (81.28-83.31)	0.97 (0.96-0.99)	0.97 (0.95-0.98)
Västra Götaland	4,391	5,326	82.44 (81.42-83.47)	0.97 (0.96-0.99)	0.97 (0.96-0.99)
Uppsala	960	1,164	82.47 (80.29-84.66)	0.97 (0.95-1.00)	0.97 (0.94-1.00)
Norrbotten	1,215	1,465	82.94 (81.01-84.86)	0.98 (0.95-1.01)	0.98 (0.95-1.00)
Östergötland	1,400	1,682	83.23 (81.45-85.02)	0.98 (0.96-1.01)	0.98 (0.96-1.01)
Dalarna	1212	1,444	83.93 (82.04-85.83)	0.99 (0.97-1.02)	0.99 (0.97-1.02)
Jämtland	501	595	84.20 (81.27-87.13)	1.00 (0.96-1.03)	1.00 (0.96-1.03)
Västerbotten	893	1,058	84.40 (82.22-86.59)	1.00 (0.97-1.03)	0.99 (0.97-1.02)
Halland	940	1,112	84.53 (82.41-86.66)	1.00 (0.97-1.03)	1.00 (0.97-1.02)
Skåne	4,085	4,828	84.61 (83.59-85.63)	Reference	Reference
Kalmar	898	1,054	85.20 (83.06-87.34)	1.01 (0.98-1.04)	1.01 (0.98-1.04)
Södermanland	973	1,141	85.28 (83.22-87.33)	1.01 (0.98-1.04)	1.01 (0.98-1.04)
Gävleborg	1,126	1,318	85.43 (83.53-87.34)	1.01 (0.98-1.04)	1.01 (0.99-1.04)
Kronoberg	592	685	86.42 (83.86-88.99)	1.02 (0.99-1.05)	1.02 (0.99-1.05)
Värmland	1,076	1,240	86.77 (84.89-88.66)	1.03 (1.00-1.05)	1.03 (1.00-1.05)
Jönköping	1,126	1,292	87.15 (85.33-88.98)	1.03 (1.01-1.06)	1.03 (1.00-1.05)
Västmanland	877	1,004	87.35 (85.29-89.41)	1.03 (1.01-1.06)	1.04 (1.01-1.06)
Area under the ROC curve (AUC)				0.537	0.618

Figure 3 and Table 2 illustrate the crude prevalence of statin dispensations for each sociodemographic stratum, with the highest prevalence (i.e., 89.5%) in the reference strata of 40–64-year-old native men with high income, followed by the strata of 40–64-year-old native men with middle income (i.e., 88.8%). We found the lowest prevalence of statin use in the stratum of 65–80-year-old immigrant women with high income (i.e., 70.3%), closely followed by 65–80-year-old immigrant women with low income (i.e., 74.1%). Only the reference sociodemographic stratum almost grasped the target value of

For peer review only - http://bmjopen.bmj.com/site/about/guidelines.xhtml

90% (i.e., 89.5%). None of the female strata were close to reaching the target. In

Table 2. Number of patients using statins (n), number of individuals (N), crude prevalence (P) and 95% confidence intervals (CI), unadjusted prevalence ratios (PR), and prevalence ratios adjusted for administrative regions (PRa) for use of statins 12-18 months after hospital discharge with an acute myocardial infarction (AMI) diagnosis in patients aged, 40-80 years old from 24 sociodemographic strata in the period 2011-2013

				Model 1B	Model 2
	n	N	P (95% CI) %	PR (95% CI)	PRa (95% CI)
Sweden	29,321	35,044	83.67 (83.28-84.06)	–	–
Women					
65-80 , High income, Immigrant	180	256	70.31 (64.72-75.91)	0.79 (0.72-0.85)	0.79 (0.73-0.85)
65-80 , Low income, Immigrant	300	405	74.07 (69.81-78.34)	0.83 (0.78-0.88)	0.83 (0.78-0.88)
65-80 , Middle income, Native	1,936	2,575	75.18 (73.52-76.85)	0.84 (0.82-0.86)	0.84 (0.82-0.86)
40-64 , Low income, Native	479	637	75.20 (71.84-78.55)	0.84 (0.80-0.88)	0.84 (0.80-0.88)
65-80 , Low income, Native	852	1,132	75.27 (72.75-77.78)	0.84 (0.81-0.87)	0.84 (0.81-0.87)
65-80 , Middle income, Immigrant	338	448	75.45 (71.46-79.43)	0.84 (0.80-0.89)	0.84 (0.80-0.89)
40-64 , Middle income, Immigrant	130	171	76.02 (69.62-82.42)	0.85 (0.78-0.92)	0.85 (0.78-0.93)
40-64 , Low income, Immigrant	197	257	76.65 (71.48-81.83)	0.86 (0.80-0.92)	0.86 (0.80-0.92)
40-64 , Middle income, Native	656	846	77.54 (74.73-80.35)	0.87 (0.83-0.90)	0.86 (0.83-0.90)
65-80 , High income, Native	1,838	2,369	77.59 (75.91-79.26)	0.87 (0.85-0.89)	0.87 (0.85-0.89)
40-64 , High income, Immigrant	92	118	77.97 (70.49-85.44)	0.87 (0.79-0.96)	0.87 (0.79-0.96)
40-64 , High income, Native	709	904	78.43 (75.75-81.11)	0.88 (0.85-0.91)	0.87 (0.84-0.91)
Men					
65-80 , Low income, Immigrant	548	679	80.71 (77.74-83.67)	0.90 (0.87-0.94)	0.90 (0.87-0.94)
65-80 , Low income, Native	1,409	1,735	81.21 (79.37-83.05)	0.91 (0.88-0.93)	0.91 (0.88-0.93)

contrast, the male strata were often much closer to reaching the target. Table 2 also presents the unadjusted PR (Model 1B), and the PR adjusted for the geographical variable (Model 2), with the strata of 40–64-year-old high-income native men as reference stratum. As many as 19 sociodemographic strata show conclusively smaller PRs than the reference. The adjusted PR were very similar to the unadjusted PR indicating that geographical factors did not confound the sociodemographic differences.

For peer review only

65-80 , Middle income, Immigrant	565	686	82.36 (79.51-85.21)	0.92 (0.89-0.95)	0.92 (0.89-0.95)
40-64 , Low income, Native	1,283	1,540	83.31 (81.45-85.17)	0.93 (0.91-0.95)	0.93 (0.91-0.95)
40-64 , Middle income, Immigrant	397	472	84.11 (80.81-87.41)	0.94 (0.90-0.98)	0.94 (0.90-0.98)
65-80 , Middle income, Native	3,791	4,458	85.04 (85.04-85.04)	0.95 (0.93-0.97)	0.95 (0.93-0.96)
40-64 , Low income, Immigrant	1,006	1,171	85.91 (83.92-87.90)	0.96 (0.94-0.98)	0.96 (0.94-0.99)
65-80 , High income, Immigrant	570	645	88.37 (88.37-88.37)	0.99 (0.96-1.02)	0.99 (0.96-1.02)
40-64 , High income, Immigrant	337	381	88.45 (85.24-91.66)	0.99 (0.95-1.03)	0.99 (0.95-1.03)
65-80 , High income, Native	6,303	7,101	88.76 (88.76-88.76)	0.99 (0.98-1.01)	0.99 (0.98-1.00)
40-64 , Middle income, Native	2,240	2,522	88.82 (87.59-90.05)	0.99 (0.97-1.01)	0.99 (0.97-1.01)
40-64 , High income, Native	3,165	3,536	89.51 (88.50-90.52)	Reference	Reference
Area under the ROC curve (AUC)				0.609	0.618

Model 1A, which includes information about administrative regions, presented an AUC value of 0.537. This AUC value indicates that the geographical differences were *absent/very small,* and that region provides very limited information about statin dispensations. Model 1B, which includes information on the sociodemographic strata, showed an AUC of 0.609 which indicates that the sociodemographic differences were *small*. The sociodemographic AUC was 0.072 units higher than the AUC of the geographical model 1A. Model 2, which includes both geographical and sociodemographic information, presented an AUC value of 0.618, just 0.008 higher than that presented by model 1B. In the context of our analysis, these findings indicates that sociodemographic information is relatively more relevant than geographic information. In summary, the target indicator value was *closely* reached in Sweden as a whole. The geographical analysis indicated *absent/very small* differences, which corresponds with scenario B in Box 1. The sociodemographic analysis indicated *small* differences, which corresponds with the scenario E in Box 1.

For peer review only - http://bmjopen.bmj.com/site/about/guidelines.xhtml

In relation to the sociodemographic differences, the complementary analysis (S1) showed that the AUC was very small when we fitted separate models which included only one variable at a time. The highest AUC was for the variable sex (AUC = 0.579) which was 0.029 units lower than the AUC for all variables together (AUC = 0.608). We also observed a very small interaction of effects in the model with the multicategorical variable as when comparing with model with all the single variables together, the AUC of the model with the multicategorical variable only increases 0.001 units.

Discussion

We evaluated regional and sociodemographic inequalities in statin treatment after AMI. When doing so, we also aimed to demonstrate how AIHDA provides enhanced information for evaluating healthcare inequalities.[9, 38] While traditional public comparisons focus on differences between group averages, AIHDA considers both differences between group averages and the individual heterogeneity around those averages as expressed by their discriminatory accuracy. AIHDA informs on the share of the total individual variation in patient outcomes that operates at the group level. That is, to which degree the group-level information is able to discriminate with accuracy patients with, from those without the indicator under study.[9] The idea of discriminatory accuracy is fundamental in many fields of epidemiology not only when evaluating statistical model

For peer review only - http://bmjopen.bmj.com/site/about/guidelines.xhtml

For peer review only

prediction,[39] but also in the evaluation of diagnostic and prognostic tests[35], as well as risk factors in public health[6] and in the study of geographical[38] and socioeconomic[19] determinates of health. It is also a cornerstone in current precision medicine.[40] Nevertheless, these concepts need wider adoption still and we refer elsewhere for a longer explanation.[6, 7, 9, 10]

The overall prevalence of statin treatment in our study population was 83.7% which while lower was judged high enough to have “closely” reached the target benchmark value of 90%. We observed regional and sociodemographic differences in the prevalence of statin treatment after AMI. However, the low AUC values indicated that the differences were “absent/very small” (AUC = 0.537) for the administrative regions and “small” (AUC = 0.609) for the sociodemographic groups. Combining both regional and sociodemographic information did not substantially increase the AUC of the sociodemographic information alone (AUC = 0.618). That is, the sociodemographic differences seem to play a modest but more relevant role than the regional differences for understanding inequalities in statin treatment after AMI.

Our analysis also shows that women in all strata have a lower prevalence of statin dispensations after AMI than men, which is in line with previous research indicating the existence of gender inequalities.[29] However, those inequalities need be interpreted in the light of the small DA of the sociodemographic strata. In fact, while the prevalence of using statins is higher among men, the fact that AMI patients are disproportionately male means that majority of non-statin users are nevertheless also men.

For peer review only - http://bmjopen.bmj.com/site/about/guidelines.xhtml

According to the framework presented in box 1, the sociodemographic differences would be placed in scenario E. That is, the target value was closely but not fully reached and there were small differences between the sociodemographic strata. Accordingly, any strategy aimed to improve the outcome of the quality indicator should be universal but proportionately more intense among sociodemographic groups with increased risk not using statins. Analogously, the differences between administrative regions would be placed in the scenario B. In this case we should recommend universal interventions without targeting any specific regions.

Strengths and limitations

In addition to an improved and more nuanced analysis, AIHDA conveys further strengths as compared to traditional studies exclusively based on differences between group averages. It is known that epidemiological studies comparing group averages may *stigmatize* the individuals belonging to groups with "bad" average values and create *false expectations* for the individuals belonging to groups with "good" average values. This situation is true for both traditional and AIHDA studies and it is an inevitable side effect in epidemiology. Nevertheless, the perils of stigmatization and false expectations are often ethically justifiable because the aim of the epidemiological analyses is to improve the health and heath care of the individuals. However, knowing the DA of the group comparisons allows us to decide whether pointing out specific population groups is justifiable or not. If the DA is high, pointing out specific population groups is justifiable.

For peer review only - http://bmjopen.bmj.com/site/about/guidelines.xhtml

However, if the DA is low, the traditional emphasis on differences between group averages should be avoided. For instance, our results show that, because the AUC value is low, differences between administrative regions are less relevant for understanding individual statin dispensations. Therefore, simplistic interpretation based on visual analyses of figures like the caterpillar plot in our study (Figure 2) should be sidestepped.

By overlooking the DA of the group-averages, traditional studies may simultaneously lead to both under and overtreatment if a possible intervention is based on group information only. If the DA is low, many individuals taking statins but belonging to a group with a “bad” average (i.e., false positives cases) could be exposed to the intervention while many individuals not taking statins but belonging to a group with a “good” average (i.e., false negative cases) would not receive any intervention. For instance, using the information in Table 1, we can see that the region of Gotland has the worst average value (i.e., 76.4%), meaning that 76 of the 322 patients were not dispensed statins. However, in the region of Västmanland with the best average value (i.e., 87.4 %), this figure was 127 of the 1004 patients since Västmanland has a larger population. That is, the “best” region has 1.7 (= 127/76) times more patients lacking statin treatment that the “worst” region. It is correct that the region of Västmanland has a higher proportion of treated patients but from a national perspective the burden of untreated patients is also higher in the region of Västmanland. Therefore, if the DA is low, any potential

For peer review only - http://bmjopen.bmj.com/site/about/guidelines.xhtml

intervention to increase statin treatment after AMI needs to be universal (i.e., on the whole country) and directed to all AMI patients no matter the region they belong to.

Similarly, in Table 2 we can estimate that the number of male AMI patients without statins (n = 3,312) was much higher than the number of female AMI patients without statins (n = 2,411) even though women consistently have a lower prevalence and a lower PR of statin dispensation than men. This reflects that while the prevalence of using statins is higher among men, the fact that AMI patients are disproportionately male means that majority of non-statin users are nevertheless also men.

One further strength of this study is the quality of the data since all information is based on national registers where the information is recorded in a highly standardized way for both ICD and ATC codes. The validity of the AMI diagnosis as recorded in the NPR is high, [41] and the sociodemographic information is recorded by Statistics Sweden with a longstanding experience in the field [https://www.scb.se/en/About-us/main-activity/quality-work/]. Moreover, we can directly compare our results to those presented by the NBHW since the definition of the quality indicator is the same.

One limitation of our study is that we excluded the 5,424 individuals who died, emigrated, or had missing information on country of birth which corresponds to approximately 13% of the final study population, and it cannot be ruled out that this exclusion introduces a selection bias. However, we do not think that this limitation alters our conclusions as the

For peer review only - http://bmjopen.bmj.com/site/about/guidelines.xhtml

selection criteria are in concordance with the those used by the Swedish authorities. Another limitation in this study could be the use of household income as a proxy for socioeconomic position, which does not capture other financial assets, nor necessarily higher education.

The definition of country of birth within this study could be elaborated with more refined categories, other than native and immigrant. Immigrant is a broad concept which includes, among other factors, cultural, economic, and educational heritages from another countries. However, more categories of country of birth would result in a larger number of multicategorical strata with a smaller number of within strata observations. Similarly, there is a possible underestimation of the impact of age since we only use two categories in our analyses. We chose two categories to keep the analysis as simple as possible and also because in the case of the quality indicator we analyze we think it is sufficient to include the age of retirement (i.e., 65 years for most patients at the time of the study) as the cut-off. It is know that this division is relevant for understanding socioeconomic differences in health care utilization.[42] However, for other quality indicators it may be better to provide more age classes.

We use the AUC as a measure of discriminatory accuracy and in some situations when e.g., false negatives are more likely to occur than false positives because of information bias, assuming the same weight for both false negatives and positive might give misleading AUC values. However, this is not a problem in biomedical applications in

For peer review only - http://bmjopen.bmj.com/site/about/guidelines.xhtml

general and in our study in particular as positive or negative events are easy to identify (a patient uses statins or not) and the information is, less probably, differentially biased. The AUC value may also depend on the size of the group being compared. For instance, a stratum may have a high predicted probability of using statins so all individuals in the stratum are classified as "positive" but if the stratum is very large the number of individuals in the stratum who do not use statins (i.e., false positive) may be large and the AUC reduced. This could be prevented by weighting the strata by the inverse of the number of individuals or using other measures of discriminatory accuracy like those based on components of variance.[9] However, in our case the unweighted results are relevant per se. A low unweighted AUC would suggest that any potential intervention to increase use of statins should not be targeted to specific strata with a low predicted probability of statin use but be universal and directed to all the strata since there are many patients in the strata with high predicted probabilities who do not use statins. For instance, in our study 37% (2,146/5,723) of the patients who did not use statins reside in the 10 counties with highest predicted probability of using statins, and this value was 58% (3,312/5,723) for the 12 sociodemographic strata with highest predicted probability of using statins.

Implications and Conclusions

Dispensations of statins after AMI in Sweden are, according to the national quality indicator, closely but not fully met in relation to the benchmark value of 90%. However,

For peer review only

neither regional nor sociodemographic differences in group averages seem to be major determinants of dispensation of statins. Therefore, any intervention aiming to reach the benchmark value should be universal. Sociodemographic differences nevertheless appeared more relevant than geographic differences. However, our results suggest that in spite the lower prevalence of statin prescription in some groups (e.g., women, especially to those with immigrant background and older than 65 years), the exclusive targeting of interventions to those groups would be unsuitable as many AMI patients lacking statin prescription are in the groups with higher prevalence of statin prescription. In other words, we cannot base decisions on stratum prevalence alone, we also need to consider the size of the strata.

In conclusion, as we illustrate in our study, AIHDA is not complicated to implement, and it provides more nuanced information than traditional comparisons of quality of healthcare performance based only on group averages. We therefore propose the application of the AIHDA framework for the quantitative assessment of healthcare quality by geographic and sociodemographic comparisons of quality indicators.

Footnotes

- **Contributors** JM had the initiative of the study and acquired the data. JM and JÖ wrote the original manuscript. RP-V and JÖ performed the analyses in coordination with JM, KK and GL. All the authors have contributed to the design of the study, JM and GL developed the methodology and directed the analyses. All

For peer review only - http://bmjopen.bmj.com/site/about/guidelines.xhtml

authors have contributed to the interpretation of the results and drafting of the manuscript. All authors have revised the last version of the manuscript.

- **Funding** The Swedish Research Council (Vetenskapsrådet) supported this work through the project 'Multilevel Analyses of Individual Heterogeneity: innovative concepts and methodological approaches in Public Health and Social Epidemiology': (#2017-01321, PI: Juan Merlo).

- **Competing interests** None declared.
- **Ethics approval** Regional ethics committee Lund: ID: 2014/856. Data safety committees from the National board of health and welfare: ID 9542/2015. Statistics Sweden: ID 231424/878144-5.
- **Patient consent for publication** Not required.
- **Provenance and peer review** Not commissioned, externally peer reviewed.
- **Data availability statement** Data are available upon reasonable request. Original data are available from the Swedish National Board of Health and Welfare and Statistics, after approval of a research project by an Ethical Committee and by the data safety committees of Swedish Authorities.

For peer review only - http://bmjopen.bmj.com/site/about/guidelines.xhtml

References

1. Socialstyrelsen. God vård – om ledningssystem för kvalitet och patientsäkerhet i hälso- och sjukvården. 2006. Contract No.: Artikelnummer: 2006-101-2.
2. Heurgren M, Åberg A, Köster M, al. e. Performance indicators in Swedish Health Care. BMC Health Serv Res. 2007;7(A10).
3. Öppna jämförelser av hälso- och sjukvårdens kvalitet och effektivitet Jämförelser mellan landsting . Sveriges Kommuner och Landsting wwwsklse/publikationer ISBN 978-91-7164-485-5. 2009.
4. Sorman H. [Open comparisons of health care: "One of the most important contributions to modernization of health care"]. Lakartidningen. 2010;107(32-33):1828-9.
5. Khalaf K, Axelsson Fisk S, Ekberg-Jansson A, Leckie G, Perez-Vicente R, Merlo J. Geographical and sociodemographic differences in discontinuation of medication for Chronic Obstructive Pulmonary Disease - A Cross-Classified Multilevel Analysis of Individual Heterogeneity and Discriminatory Accuracy (MAIHDA). Clin Epidemiol. 2020;12:783-96.
6. Merlo J, Mulinari S, Wemrell M, Subramanian S V, Hedblad B. The tyranny of the averages and the indiscriminate use of risk factors in Public Health: The case of coronary heart disease. SSM - Population Health. 2017;3:684-98.
7. Merlo J. Invited commentary: multilevel analysis of individual heterogeneity-a fundamental critique of the current probabilistic risk factor epidemiology. American journal of epidemiology. 2014;180(2):208-12; discussion 13-4.
8. Merlo J. Multilevel analytical approaches in social epidemiology: measures of health variation compared with traditional measures of association. J Epidemiol Community Health. 2003;57(8):550-2.
9. Merlo J, Wagner P, Leckie G. A simple multilevel approach for analysing geographical inequalities in public health reports: The case of municipality differences in obesity. Health Place. 2019;58:102145.
10. Merlo J. Multilevel analysis of individual heterogeneity and discriminatory accuracy (MAIHDA) within an intersectional framework. Soc Sci Med. 2018;203:74-80.
11. Merlo J. Flernivåanalyser behövs i öppna jämförelser inom hälso- och sjukvård. Läkartidningen 2019;116:FTEL. 2019.
12. Evans CR, Erickson N. Intersectionality and depression in adolescence and early adulthood: A MAIHDA analysis of the national longitudinal study of adolescent to adult health, 1995-2008. Soc Sci Med. 2019;220:1-11.
13. Mulinari S, Bredstrom A, Merlo J. Questioning the discriminatory accuracy of broad migrant categories in public health: self-rated health in Sweden. Eur J Public Health. 2015;25(6):911-7.
14. Ljungman H, Wemrell M, Khalaf K, Perez-Vicente R, Leckie G, Merlo J. Antidepressant use in Sweden: an intersectional multilevel analysis of individual heterogeneity and discriminatory accuracy (MAIHDA). Scand J Public Health. 2021:1403494821993723.
15. Axelsson Fisk S, Lindstrom M, Perez-Vicente R, Merlo J. Understanding the complexity of socioeconomic disparities in smoking prevalence in Sweden: a cross-sectional study applying intersectionality theory. BMJ Open. 2021;11(2):e042323.
16. Axelsson Fisk S, Mulinari S, Wemrell M, Leckie G, Perez Vicente R, Merlo J. Chronic Obstructive Pulmonary Disease in Sweden: An intersectional multilevel analysis of individual heterogeneity and discriminatory accuracy. SSM Popul Health. 2018;4:334-46.
17. Wemrell M, Bennet L, Merlo J. Understanding the complexity of socioeconomic disparities in type 2 diabetes risk: a study of 4.3 million people in Sweden. BMJ Open Diabetes Res Care. 2019;7(1):e000749.
18. Hernandez-Yumar A, Wemrell M, Abasolo Alesson I, Gonzalez Lopez-Valcarcel B, Leckie G, Merlo J. Socioeconomic differences in body mass index in Spain: An intersectional multilevel analysis of individual heterogeneity and discriminatory accuracy. PLoS One. 2018;13(12):e0208624.

For peer review only - http://bmjopen.bmj.com/site/about/guidelines.xhtml

19. Persmark A, Wemrell M, Zettermark S, Leckie G, Subramanian SV, Merlo J. Precision public health: Mapping socioeconomic disparities in opioid dispensations at Swedish pharmacies by Multilevel Analysis of Individual Heterogeneity and Discriminatory Accuracy (MAIHDA). PLoS One. 2019;14(8):e0220322.
20. Rodriguez-Lopez M, Merlo J, Perez-Vicente R, Austin P, Leckie G. Cross-classified Multilevel Analysis of Individual Heterogeneity and Discriminatory Accuracy (MAIHDA) to evaluate hospital performance: the case of hospital differences in patient survival after acute myocardial infarction. BMJ Open. 2020;10(10):e036130.
21. Ghith N, Frolich A, Merlo J. The role of the clinical departments for understanding patient heterogeneity in one-year mortality after a diagnosis of heart failure: A multilevel analysis of individual heterogeneity for profiling provider outcomes. PLoS One. 2017;12(12):e0189050.
22. Wemrell M, Karlsson N, Perez Vicente R, Merlo J. An intersectional analysis providing more precise information on inequities in self-rated health. Int J Equity Health. 2021;20(1):54.
23. Ljungman H, Wemrell M, Khalaf K, Perez-Vicente R, Leckie G, Merlo J. Antidepressant use in Sweden: an intersectional multilevel analysis of individual heterogeneity and discriminatory accuracy (MAIHDA). Scand J Public Health. 2022;50(3):395-403.
24. Zettermark S, Khalaf K, Perez-Vicente R, Leckie G, Mulinari D, Merlo J. Population heterogeneity in associations between hormonal contraception and antidepressant use in Sweden: a prospective cohort study applying intersectional multilevel analysis of individual heterogeneity and discriminatory accuracy (MAIHDA). BMJ Open. 2021;11(10):e049553.
25. Evans CR, Leckie G, Merlo J. Multilevel versus single-level regression for the analysis of multilevel information: The case of quantitative intersectional analysis. Soc Sci Med. 2020;245:112499.
26. Lizotte DJ, Mahendran M, Churchill SM, Bauer GR. Math versus meaning in MAIHDA: A commentary on multilevel statistical models for quantitative intersectionality. Soc Sci Med. 2020;245:112500.
27. Bauer GR, Churchill SM, Mahendran M, Walwyn C, Lizotte D, Villa-Rueda AA. Intersectionality in quantitative research: A systematic review of its emergence and applications of theory and methods. SSM Popul Health. 2021;14:100798.
28. Socialstyrelsen. Nationella riktlinjer för hjärtsjukvård Stöd för styrning och ledning. Stockholm, Sweden; 2018.
29. Ruiz-Cantero MT, Blasco-Blasco M, Chilet-Rosell E, Peiro AM. Gender bias in therapeutic effort: from research to health care. Farm Hosp. 2020;44(3):109-13.
30. Chang AY, Abou-Arraj NE, Rodriguez F. Interventions to Reduce Ethnic and Racial Disparities in Dyslipidemia Management. Current treatment options in cardiovascular medicine. 2019;21(5):24.
31. Wylie LE, Waterbrook AL, Dalen JE. Are Statins Indicated in Senior Citizens? A Review of Statin Therapy in the Elderly. Am J Med. 2021.
32. Hyun KK, Brieger D, Woodward M, Richtering S, Redfern J. The effect of socioeconomic disadvantage on prescription of guideline-recommended medications for patients with acute coronary syndrome: systematic review and meta-analysis. Int J Equity Health. 2017;16(1):162.
33. Barros AJ, Hirakata VN. Alternatives for logistic regression in cross-sectional studies: an empirical comparison of models that directly estimate the prevalence ratio. BMC Med Res Methodol. 2003;3:21.
34. Hosmer DW, Lemeshow S. Applied logistic regression. 2nd ed. New York: Wiley; 2000. xii, 373 p. p.
35. Pepe M. S., Janes H., Longton G., Leisenring W., Newcomb P. Limitations of the odds ratio in gauging the performance of a diagnostic, prognostic, or screening marker. American journal of epidemiology. 2004;159(9):882-90.
36. Marmot M, Bell R. Fair society, healthy lives. Public Health. 2012;126 Suppl 1:S4-S10.
37. Carey G, Crammond B, De Leeuw E. Towards health equity: a framework for the application of proportionate universalism. Int J Equity Health. 2015;14:81.

For peer review only - http://bmjopen.bmj.com/site/about/guidelines.xhtml

38. Merlo J, Wagner P, Ghith N, Leckie G. An Original Stepwise Multilevel Logistic Regression Analysis of Discriminatory Accuracy: The Case of Neighbourhoods and Health. PLoS One. 2016;11(4):e0153778. https://doi.org/10.1371/journal.pone.
39. Moons KG, de Groot JA, Bouwmeester W, Vergouwe Y, Mallett S, Altman DG, et al. Critical appraisal and data extraction for systematic reviews of prediction modelling studies: the CHARMS checklist. PLoS medicine. 2014;11(10):e1001744.
40. Ginsburg GS, Phillips KA. Precision Medicine: From Science To Value. Health Aff (Millwood). 2018;37(5):694-701.
41. Ludvigsson JF, Andersson E, Ekbom A, Feychting M, Kim JL, Reuterwall C, et al. External review and validation of the Swedish national inpatient register. BMC Public Health. 2011;11:450.
42. Merlo J, Gerdtham UG, Lynch J, Beckman A, Norlund A, Lithman T. Social inequalities in health – do they diminish with age? Revisiting the question in Sweden 1999. Int J Equity Health. 2003;2(1):2.

For peer review only

Figure 1: Study population flow-chart

Figure 2: Crude prevalence (P) and 95% confidence intervals (CI) for use of statins 12 -18 months after hospital discharge with an acute myocardial infarction (AMI) diagnosis in patients aged 40-80 years in the period 2011-2013, across the 21 administrative regions of Sweden.

Figure 3: Crude prevalence (P) and 95% confidence intervals (CI) for use of statins 12 -18 months after hospital discharge with an acute myocardial infarction (AMI) diagnosis in patients aged 40-80 years in the period 2011-2013, across the 24 sociodemographic groups.

For peer review only

For peer review only - http://bmjopen.bmj.com/site/about/guidelines.xhtml

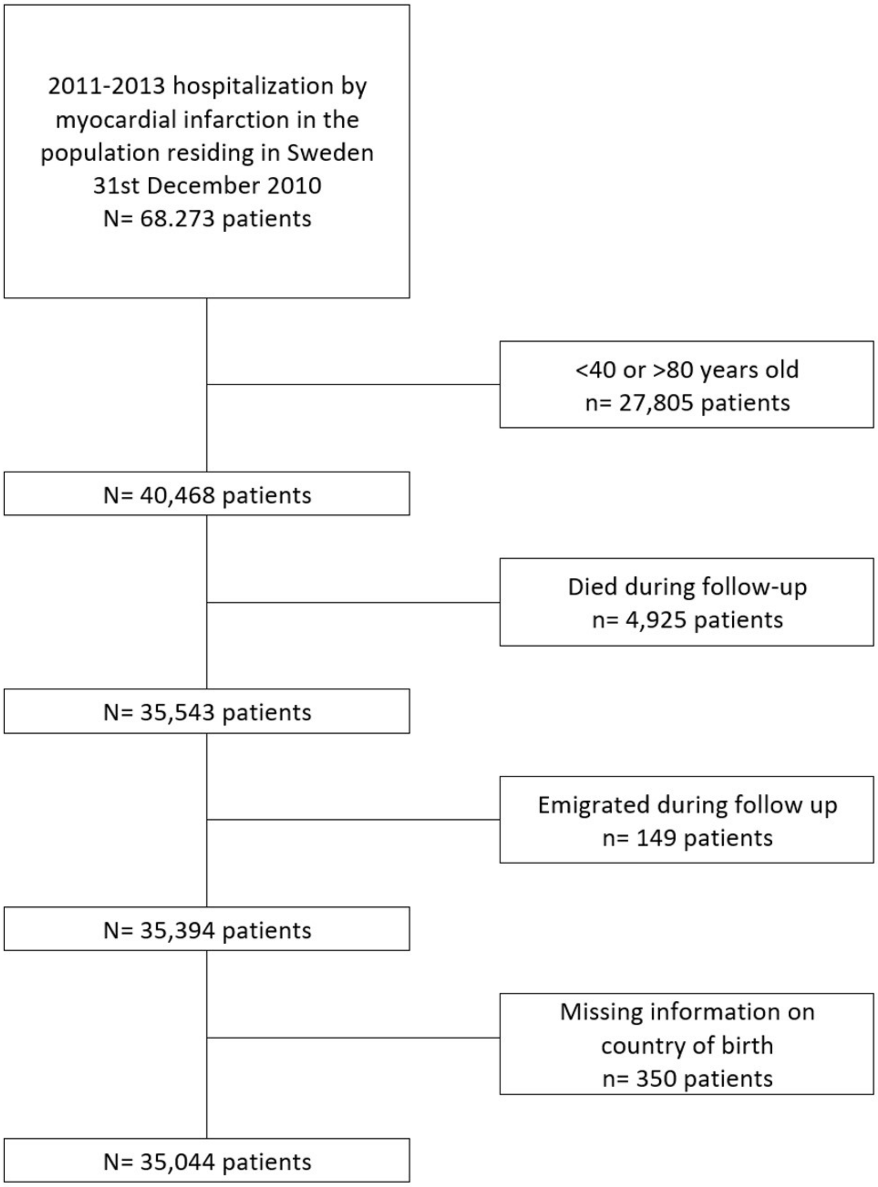

Figure 1: Study population flow-chart

261x348mm (96 x 96 DPI)

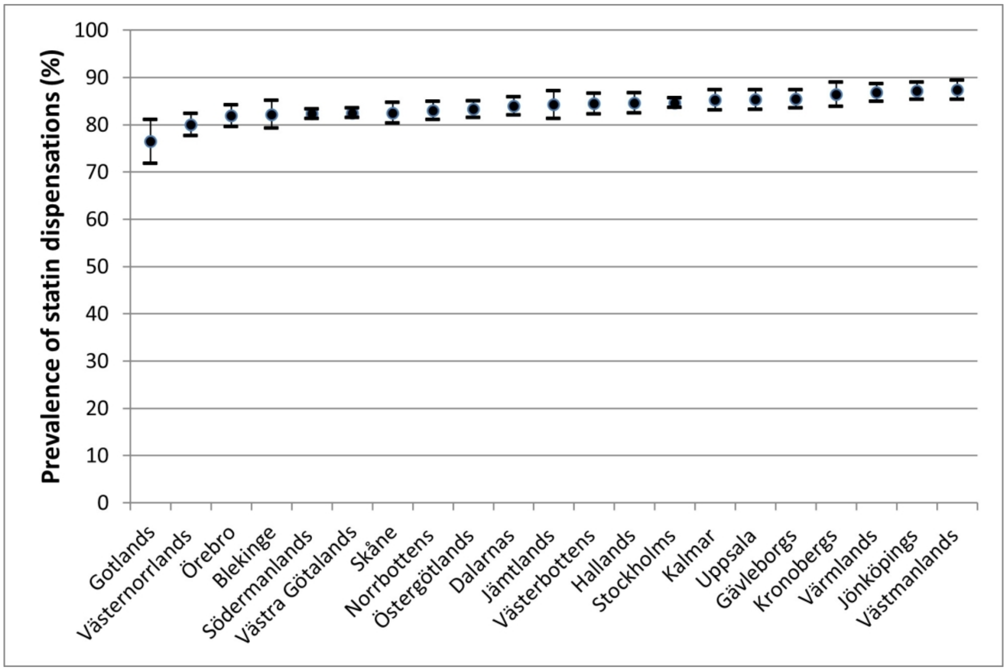

Figure 2: Crude prevalence (P) and 95% confidence intervals (CI) for use of statins 12 -18 months after hospital discharge with an acute myocardial infarction (AMI) diagnosis in patients aged 40-80 years in the period 2011-2013, across the 21 administrative regions of Sweden.

152x101mm (330 x 330 DPI)

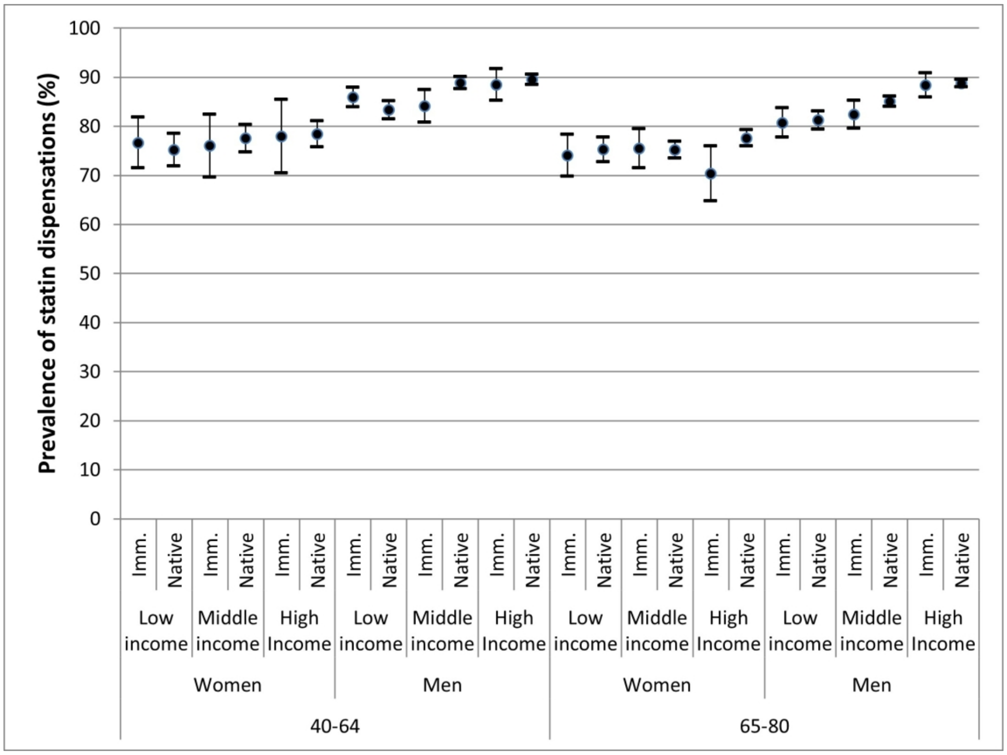

Figure 3: Crude prevalence (P) and 95% confidence intervals (CI) for use of statins 12 -18 months after hospital discharge with an acute myocardial infarction (AMI) diagnosis in patients aged 40-80 years in the period 2011-2013, across the 24 sociodemographic groups.

165x123mm (330 x 330 DPI)

SUPPLEMENTARY MATERIAL (S1)

S1: Stata codes

Main analysis

```
. use cohort_selected.dta, clear

. generate time = 1
. stset time, failure(statins) id(lpnr)

* Model 1a
. stcox ib11.region, vce(robust)
. predict p1a

* Model 1b
. stcox ib12.inter, vce(robust)
. predict p1b

* Model 2
. stcox ib11.region ib12.inter, vce(robust)
. predict p2

. roccomp statins p1a p1b p2
```

Prevalences

```
. use cohort_selected.dta, clear
. by region, sort: egen cases = sum(statins)
. by region: gen total = _N
. keep cases total region
. duplicates drop

. use cohort_selected.dta, clear
. by inter, sort: egen cases = sum(statins)
. by inter: generate total = _N
. keep cases total inter
. duplicates drop

. use cohort_selected.dta, clear
. egen cases = sum(statins)
. display _N
. tabulate cases
```


For peer review only - http://bmjopen.bmj.com/site/about/guidelines.xhtml

Supplementary analysis

```
. use cohort_selected.dta, clear

. generate time = 1
. stset time, failure(statins) id(lpnr)

* Model 1b_age
. stcox ib1.age_cat, vce(robust)
. predict p2a

* Model 1b_sex
. stcox i.male, vce(robust)
. predict p2b

* Model 1b_income
. stcox i.cum, vce(robust)
. predict p2c

* Model 1b_country
. stcox i.Sw, vce(robust)
. predict p2d

* Model 1b_all
. stcox ib1.age_cat i.male i.cum i.Sw, vce(robust)
. predict p2e

. roccomp statins p2*
```

For peer review only

For peer review only - http://bmjopen.bmj.com/site/about/guidelines.xhtml

SUPPLEMENTARY MATERIAL (S2)

S2: Supplementary analyses

Prevalence Ratios (PR) and (95% confidence intervals) for use of statins 12-18 months after hospital discharge with an acute myocardial infarction (AMI) diagnosis in patients aged, 40-80 years old from 24 sociodemographic strata in the period 2011-2013

	Age	Sex	Income	Country of birth	All	Multicategorical *
Age						
40-64	1.03 (1.02-1.04)				1.02 (1.01-1.04)	
65-80	Reference					
Sex						
Women		Reference				
Men		1.14 (1.12-1.15)			1.13 (1.12-1.14)	
Income						
Low			Reference			
Middle			1.03 (1.01-1.04)		1.03 (1.01-1.04)	
High			1.07 (1.06-1.09)		1.06 (1.05-1.08)	
Country of birth						
Immigrant				Reference		
Native				1.03 (1.01-1.04)	1.01 (1.00-1.03)	
AUC	0.512	0.579	0.546	0.511	0.608	0.609

*Model 1B in table 2. AUC: area under the ROC curve

For peer review only

STROBE Statement—Checklist of items that should be included in reports of ***cross-sectional studies***

	Item No	Recommendation	Page No
**Title and abstract**	1	(*a*) Indicate the study's design with a commonly used term in the title or the abstract	1-2
		(*b*) Provide in the abstract an informative and balanced summary of what was done and what was found	2
**Introduction**			
Background/rationale	2	Explain the scientific background and rationale for the investigation being reported	4-7
Objectives	3	State specific objectives, including any prespecified hypotheses	4-6
**Methods**			
Study design	4	Present key elements of study design early in the paper	7-8
Setting	5	Describe the setting, locations, and relevant dates, including periods of recruitment, exposure, follow-up, and data collection	4, 7-8
Participants	6	(*a*) Give the eligibility criteria, and the sources and methods of selection of participants	7-8
Variables	7	Clearly define all outcomes, exposures, predictors, potential confounders, and effect modifiers. Give diagnostic criteria, if applicable	8-9
Data sources/ measurement	8*	For each variable of interest, give sources of data and details of methods of assessment (measurement). Describe comparability of assessment methods if there is more than one group	7-9
Bias	9	Describe any efforts to address potential sources of bias	7-9
Study size	10	Explain how the study size was arrived at	7-8
Quantitative variables	11	Explain how quantitative variables were handled in the analyses. If applicable, describe which groupings were chosen and why	7-9
Statistical methods	12	(*a*) Describe all statistical methods, including those used to control for confounding	9-14
		(*b*) Describe any methods used to examine subgroups and interactions	9-14
		(*c*) Explain how missing data were addressed	7-8
		(*d*) If applicable, describe analytical methods taking account of sampling strategy	
		(*e*) Describe any sensitivity analyses	
**Results**			
Participants	13*	(a) Report numbers of individuals at each stage of study—eg numbers potentially eligible, examined for eligibility, confirmed eligible, included in the study, completing follow-up, and analysed	7-8
		(b) Give reasons for non-participation at each stage	7-8, 28
		(c) Consider use of a flow diagram	28
Descriptive data	14*	(a) Give characteristics of study participants (eg demographic, clinical, social) and information on exposures and potential confounders	15, 17, 28
		(b) Indicate number of participants with missing data for each variable of interest	28
Outcome data	15*	Report numbers of outcome events or summary measures	15, 17

For peer review only - http://bmjopen.bmj.com/site/about/guidelines.xhtml

Main results	16	(*a*) Give unadjusted estimates and, if applicable, confounder-adjusted estimates and their precision (eg, 95% confidence interval). Make clear which confounders were adjusted for and why they were included	15, 17
		(*b*) Report category boundaries when continuous variables were categorized	8-9, 15, 17
		(*c*) If relevant, consider translating estimates of relative risk into absolute risk for a meaningful time period	
Other analyses	17	Report other analyses done—eg analyses of subgroups and interactions, and sensitivity analyses	
**Discussion**			
Key results	18	Summarise key results with reference to study objectives	14-18
Limitations	19	Discuss limitations of the study, taking into account sources of potential bias or imprecision. Discuss both direction and magnitude of any potential bias	20-23
Interpretation	20	Give a cautious overall interpretation of results considering objectives, limitations, multiplicity of analyses, results from similar studies, and other relevant evidence	20-23
Generalisability	21	Discuss the generalisability (external validity) of the study results	20-23
**Other information**			
Funding	22	Give the source of funding and the role of the funders for the present study and, if applicable, for the original study on which the present article is based	24

*Give information separately for exposed and unexposed groups.

Note: An Explanation and Elaboration article discusses each checklist item and gives methodological background and published examples of transparent reporting. The STROBE checklist is best used in conjunction with this article (freely available on the Web sites of PLoS Medicine at http://www.plosmedicine.org/, Annals of Internal Medicine at http://www.annals.org/, and Epidemiology at http://www.epidem.com/). Information on the STROBE Initiative is available at www.strobe-statement.org.

For peer review only
